# Temperature-humidity synergistic effects on predominant intestinal infectious diseases in Shenzhen, China: A predictive modeling framework for epidemiological early warning systems

**DOI:** 10.1371/journal.pone.0337929

**Published:** 2025-12-05

**Authors:** Hongxin Lyu, Zhen Zhang, Haozheng Zhou, Yan Ren, Minhua Chen, Yu Zeng, Yanpeng Cheng, Huawei Xiong

**Affiliations:** 1 Department of Epidemiology and Infectious Disease Control, Shenzhen Longhua Center for Disease Control and Prevention, Shenzhen, Guangdong, China; 2 Department of Infectious Disease Prevention and Control, Shenzhen Center for Disease Control and Prevention, Shenzhen, Guangdong, China; 3 School of Public Health, Southern Medical University, Guangzhou, Guangdong, China; 4 Department of Epidemiology, Shenzhen Longgang Center for Disease Control and Prevention, Shenzhen, Guangdong, China; Tribhuvan University, NEPAL

## Abstract

The prevalence of intestinal infectious diseases (IIDs) in densely populated cities substantially escalates the burden of disease. An ecological study on the epidemiological trends of predominant IIDs—hand, foot, and mouth disease (HFMD), viral diarrhea (DV), and non-viral diarrhea (DN)—was conducted in Shenzhen, China (2012–2022), aiming to elucidate the associations of meteorological factors, including extreme temperatures, seasonal variations, and temperature-humidity interactions with IID transmission, and to establish a monitoring and early warning framework. We assessed temperature-related morbidity’s short-term lagged effects, long-term cumulative impacts, and seasonal patterns using distributed lag non-linear models, finding that high temperatures (30°C) significantly increase HFMD and DN risks, low temperatures (13.8°C) worsen DV infections, and high temperatures have specific pathogenic effects on individuals aged 0 and over 20 years. Humidity-mediated seasonal variations in the incidence of IIDs were identified within equivalent temperature ranges. Generalized additive models (GAMs) further decoded the incidence patterns of IIDs across population subgroups under temperature-humidity interactions. An early warning model based on temperature-humidity indicators successfully predicted HFMD and DV outbreaks, establishing a novel framework for future epidemiological analyses grounded in temperature-humidity evidence. This study provides actionable insights for optimizing region-specific public health interventions and strengthening early risk mitigation strategies.

## Introduction

Globally, climate change poses significant threats to human health. Studies have shown that non-optimal temperatures are closely associated with human mortality [1]. In addition to cold conditions elevating mortality risks [2], factors such as wind speed and precipitation levels also influence the incidence of intestinal infectious diseases (IIDs) [3]. According to the International Classification of Diseases, 11th Revision (ICD-11), IIDs refer to conditions involving gastrointestinal tract infections (from the stomach to the colon) caused by bacteria, viruses, parasites, and other pathogens. These diseases are characterized by symptoms such as diarrhea, vomiting, and abdominal pain, with an onset duration of less than two weeks, manifesting as acute gastroenteritis. In recent years, IIDs have been a significant global public health challenge, especially in developing regions like sub-Saharan Africa and South Asia, where they are among the leading causes of morbidity and mortality [4]. In China, weather changes have exacerbated the burden of acute infectious diseases such as IIDs, hampering national disease prevention and control efforts [5–7].

In the realm of IIDs, hand, foot, and mouth disease (HFMD) and infectious diarrhea pose significant public health challenges on a global scale. Etiologically, enterovirus 71 (EV71) and coxsackievirus A16 (CVA16) are the predominant pathogens causing HFMD, particularly in Asia. For instance, EV71 is associated with severe complications such as neurogenic pulmonary edema and encephalitis, while CVA16 typically causes milder symptoms [8]. Similarly, infectious diarrhea remains a leading cause of childhood morbidity, with viral pathogens like rotavirus and norovirus responsible for approximately 70% of cases in children under 5 years [9]. In contrast to pediatric cases, bacterial pathogens including Salmonella and Shigella are more frequently implicated in infectious diarrhea among adults, though viral agents (e.g., norovirus) also contribute significantly [10].

Notably, HFMD and infectious diarrhea in China are the top two Class C notifiable infectious diseases [5]. They mainly affect children, with millions of annual cases nationwide. For example, China reported an annual HFMD incidence of 134.59 per 100,000 population from 2008 to 2017, peaking in southern regions [6]. Shenzhen, a populous metropolis in southern China, has persistently high incidence rates of HFMD, viral diarrhea (DV), and non-viral diarrhea (DN), ranging from 265 to 723 per 100,000 population from 2006 to 2021 [7,11]. Collectively, these three diseases account for over 80% of Shenzhen’s IIDs burden, surpassing rates observed in other cities of comparable scale [12]. Nowadays, the absence of a universal vaccine, coupled with the rampant spread of CVA16, continues to pose an ongoing threat of HFMD [13]. Concurrently, viral diarrhea, driven primarily by rotavirus and norovirus, threatens children under 3 years, while non-viral diarrhea primarily affects Shenzhen’s migrant worker population [14,15].

The global burden of IIDs demands robust surveillance systems. Modern strategies emphasize early detection, multi-source data integration, and real-time reporting. The WHO coordinates global efforts via networks like GOARN, standardizing data sharing and response protocols [16]. Innovations such as big data analytics, portable biosensors, and AI enhance surveillance by predicting outbreak risks and antibiotic resistance trends. Furthermore, climate variables, such as temperature and precipitation, are increasingly integrated into infectious disease surveillance to predict outbreak risks. For instance, temperature increases correlate with expanded ranges of Borrelia burgdorferi (Lyme disease) and Cryptosporidium incidence, while heavy rainfall amplifies waterborne pathogen transmission [17]. Surveillance systems employ geospatial tracking of climate-disease linkages, leveraging historical data to model future risks under climate change scenarios [18]. These approaches emphasize adaptive frameworks to address climate-driven pathogen dynamics and cascading public health threats.

Current surveillance systems in China, reliant on sentinel hospital data, suffer from temporal limitations, hindering early epidemic detection and risk assessment. Seasonal patterns further complicate control measures. In particular, Shenzhen’s unique subtropical monsoon climate—characterized by year-round warmth, high humidity, and minimal seasonal temperature variation—creates an ecological niche favoring IIDs transmission. Coupled with a population density of 8,806 individuals/km^2^, these conditions necessitate tailored investigations of the relationship between epidemiology and meteorological factors.

Recent studies suggest that meteorological factors have a significant effect on HFMD and infectious diarrhea. Temperature emerges as a key meteorological driver, with each 1°C increase elevating HFMD risk by 5% [19]. Extreme temperatures also amplify diarrheal disease susceptibility, though urban-specific variations remain underexplored [20]. While existing studies emphasize temperature’s role, critical gaps persist regarding its differential impacts across age and gender groups, as well as interactions with humidity—a factor positively correlated with diarrheal outbreaks [21]. For instance, drought conditions in Peru were linked to heightened diarrheal risk among children, and a study in Zhejiang found the probability of IIDs infection positively correlated with humidity brought about by rainfall, highlighting the need for multifactorial analyses [22,23].

Along with methodological advances, studies have increasingly focused on the relationship between meteorological factors and the incidence of IIDs. Traditional methods have been supplemented by more sophisticated approaches like distributed lag nonlinear models (DLNMs) and generalized additive models (GAMs) to capture the complex, nonlinear interactions between climate variables and disease dynamics [24,25]. However, current research primarily focuses on individual meteorological factors and often fails to comprehensively account for the complex interactions among multiple variables. Identifying interactive effects between two strongly correlated meteorological factors on disease incidence could prove more meaningful for establishing early warning systems [26]. The impact of meteorological factors on disease incidence exhibits significant regional variations, necessitating location-specific studies to capture these differences [27]. From early warning perspective, existing methodologies can be leveraged to develop highly specific and sensitive early warning indicator systems, thereby enhancing the precision of public health interventions.

Despite evidence linking meteorological factors to IIDs, intercity heterogeneity underscores the need for localized, long-term analyses [21]. Existing research predominantly focuses on extreme weather or pediatric populations, neglecting comprehensive risk stratification across demographics. This study aims to address these gaps by analyzing 11-year epidemiological data from Shenzhen, integrating advanced statistical models to elucidate age- and gender-specific risks. Such insights will inform targeted prevention strategies, ultimately mitigating the public health impact of IIDs in high-density urban settings.

## Materials and methods

**Fig 1:**
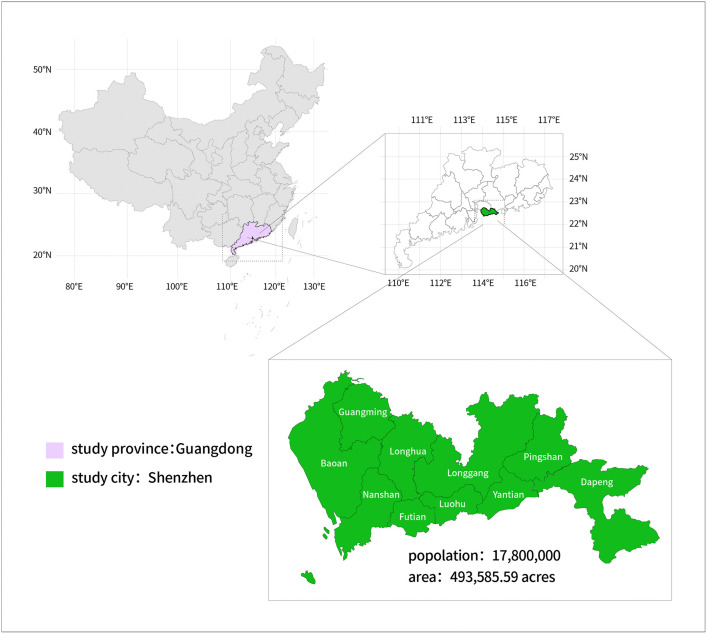
Map of the study area with high population concentration within Guangdong, China. Base map from Resource and Environment Science and Data Center (https://doi.org/10.12078/2023120601). Created by author, licensed under CC BY 4.0.

All figures presented in this manuscript were created by the authors and are licensed under a Creative Commons Attribution (CC BY) 4.0 license. The area of this study is Shenzhen City, Guangdong Province, China, with a population of approximately 36 people per acre. It is a densely populated city, as shown in Fig 1.

### Data collection

The daily incidence data were collected from January 2012 to December 2022 for the three predominant intestinal infectious diseases in Shenzhen - hand, foot and mouth disease (HFMD), viral diarrhea (DV), and non-viral diarrhea (DN) – which were selected based on their consistently highest disease burden, etiological representativeness (covering major viral and non-viral pathogens), and data reliability within the local surveillance system. The data access was performed between March 1 and March 5, 2025, through the National Notifiable Infectious Disease Reporting System of the China Information System for Disease Control and Prevention (CISDCP). The dataset included demographic characteristics (age and gender), standardized residential addresses, date of symptom onset, and diagnostic classification. For HFMD case inclusion, we considered both clinically diagnosed and laboratory-confirmed cases according to national surveillance criteria. DV cases were restricted to laboratory-confirmed enterovirus-positive infections, while DN cases comprised those with confirmed non-enterovirus pathogens (bacterial or parasitic etiologies). This rigorous case definition protocol ensured data reliability and etiological specificity for subsequent epidemiological analyses. This study has been reviewed and approved by the Shenzhen Longhua District Center for Disease Control and Prevention (Shenzhen Longhua District Health Supervision Institute) Ethics Committee (NO.2025001) and has been granted a waiver of informed consent.

Daily meteorological parameters for the same period as the collected cases were obtained from Shenzhen Meteorological Bureau (Station ID: 59493) and categorized into two distinct strata: core parameters comprising ambient temperature (Tm) and relative humidity (Um), and ancillary parameters including barometric pressure (Pm), wind speed (FFm), and precipitation (RRa) – a classification framework guided by their biometeorological relevance to disease transmission dynamics. These meteorological data were accessed from March 1 to March 5, 2025. Total precipitation was calculated as the sum of hourly precipitation measurements, whereas other meteorological parameters were aggregated through averaging measurements recorded at four standardized observation periods (2:00, 8:00, 14:00, and 20:00 local time) – a methodological distinction designed to enhance temporal consistency. Unlike typically airborne-transmitted respiratory infections, IIDs predominantly propagate through contact-mediated pathways, a methodological consideration that necessitated the exclusion of air quality parameters from our predictive models to align with their distinct transmission dynamics. The meteorological data exhibited high completeness (<2% missing values), with non-numeric features systematically processed and missing values comprehensively imputed using R’s Hmisc package.

### Risk analysis of temperature conditions

Thermodynamic exposure emerged as the principal determinant of disease incidence in this ecological study [28]. Following temporal alignment of daily case reports with meteorological parameters, we constructed structured epidemiological datasets using RStudio (v4.3.1). Case demographics were systematically categorized by gender (male/female) and stratified into four age cohorts: neonates (0 years), preschool (1–5 years), school-age (6–20 years), and adults (>20 years). Extreme cold/heat thresholds were operationalized as the 5th and 95th percentiles of temperature distribution. Extreme temperature analyses focused on characterizing lag-response profiles, whereas non-extreme thermal exposure assessments quantified cumulative risks per 1°C increment within moderate climate ranges. Furthermore, leveraging Shenzhen’s subtropical monsoon climate patterns, we conducted comparative risk analyses between the austral winter season (August-January, SS) and austral warm season (February-July, AW) under equivalent temperature exposures. The analysis was conducted using a Distributed Lag Non-linear Model (DLNM).

Multicollinearity was evaluated using variance inflation factors (VIFs). Although all other VIFs were acceptable (< 5), barometric pressure had the highest value (5.05), indicating moderate multicollinearity. Considering this and its strong correlation with temperature (r = −0.85), pressure was excluded from the final model to enhance robustness, prioritizing temperature as the primary meteorological variable [29]. To construct the model’s link function, a quasi-Poisson regression was employed to account for potential overdispersion in daily case counts observed within densely populated urban settings. Based on a comprehensive assessment of the lagged effects of climatic factors on the three main infectious intestinal diseases, the study adopted a maximum lag of 21 days [30,31]. The mathematical formulation of the model was structured as follows:


$$\begin{array}{c} Log[E(Y_{t})]=\alpha \;+\;cb({Tempt}_{t},lag)+\sum ns({Weather}_{t},df\;=\;\text{3})+ns({Time}_{t},df\;=\;\text{7}/year)+as.factor({Dow}_{t})\\ +as.factor({Holiday}_{t})+{\beta res}_{t-\text{1}} \end{array}$$

In the formula, *Y*_*t*_ represents the number of cases on day t; *α* represents the intercept term; *cb* represents the cross basis function, *ns* represents the natural cubic spline function; *Temp*_*t*_, *lag,* and *Weather*_t_ respectively represent average temperature, maximum lag time, and other meteorological factors on day t; *Time*_t_ represents the time series of day t; *df* is the degree of freedom, and initial degrees of freedom for non-temperature meteorological factors and time are set to 3 and 7/year respectively. *Dow*_t_ is a multiclass variable indicating whether day t falls on Monday to Sunday; Holiday is a binary variable representing Chinese national statutory holidays and vacations for school-age children; *res*_*t-1*_ represents the first-order lag term of model residuals with corresponding coefficient β. The degrees of freedom for meteorological factors and lag days are determined based on the minimum principle of the Quasi Akaike information criterion (QAIC).

### Prediction of incidence in different demographic population with temperature and humidity interaction

The thermoregulatory effects of ambient temperature demonstrate humidity-dependent modulations. To address this bioclimatic interaction, we implemented a generalized additive modeling framework (GAM) stratified across population subgroups with distinct demographic profiles, systematically incorporating bidirectional temperature-humidity interaction terms to probabilistically forecast disease incidence through meteorologically-informed epidemiological simulations. Given the rarity of cases, we assumed a Poisson distribution with the logarithm function selected as the link function. The formulated model is presented below:


$$Log[E(Y_{t})]=\beta +s(k_{1},k_{2})+ti(k_{1},k_{2},df)+strata$$

E(Y_t_) is the expected value of cases in day t. *s* represents the spline function that captures the non-linear relationship between temperature and humidity. *ti* is a tensor product smoother used to capture interactions, with *k1* and *k2* representing Tm and Um respectively. Strata refers to stratification variables based on age and gender.

### Establishment of early warning model

To eliminate the potential bias introduced by urban lockdowns during the COVID-19 pandemic, we constructed a random forest early warning model using incidence data prior to 2019 [32]. Specifically, incidence data from 2012 to 2016 were used as the training dataset to predict the incidence in 2017 and 2018 and issue corresponding warning signals. After considering the combined effects of temperature and humidity, we included temperature, humidity, humidex index, and the incidence of the previous week as feature variables. The humidity index can reflect the combined effect of air temperature and humidity, defined as:


$$Humidex=tem+\frac{\text{5}}{\text{9}}\times \left\{6.11\times 10^{\left(\frac{7.5\times tem}{237}+tem\right)}\times \frac{RH}{100}-10\right\}$$

The *tem* and *RH* represented temperature and humidity respectively. And we evaluate the predictive performance using the Root Mean Square Error (RMSE) and the coefficient of determination (R²) as follows:


$$RMSE=\sqrt{\frac{\text{1}}{N}\sum_{t=1}^{N}{\left(y_{t}-{\hat{y}}_{t}\right)}^{2}}$$

$$R^{2}=1-\frac{\sum {\mathrm{\backslash nolimits}}_{t=1}^{N}{\left(y_{t}-{\hat{y}}_{t}\right)}^{2}}{\sum {\mathrm{\backslash nolimits}}_{t=1}^{N}(y_{t}-\overset{―}{y}{)}^{2}}$$

The $y_{t}$ is the actual number of cases and $\overset{―}{y}$ is the average of the $y_{t}$. The ${\hat{y}}_{t}$ denotes the simulation results.

We defined the 70th, 80th, and 90th percentiles of historical incidence as the prespecified thresholds based on previous study. Incidence values less than or equal to the threshold were classified as normal (0), while those exceeding the threshold were classified as abnormal (1). The Receiver Operating Characteristic (ROC) curve was used to determine the optimal predictive threshold range, with the value corresponding to the highest Youden’s index (YI) selected as the predictive threshold. The effectiveness of the model under different prespecified thresholds was assessed using sensitivity and specificity. The relevant formulas are as follows:


$$Sensitivity=\frac{True\;Positives}{True\;Positives+False\;Negatives}\times 100\%$$

$$Specificity=\frac{True\;Negatives}{True\;Negatives+False\;Positives}\times 100\%$$

### Statistical analysis

The study utilizes the median to describe central tendency and provides minimum, maximum, and percentile values (P5, P50, and P95) for the research variables (Table 1). Non-parametric tests are employed for data that do not follow a normal distribution at a significance level of α = 0.05. Model stability is assessed through sensitivity analysis by varying lag parameters and controlling for autocorrelation to test result robustness. Data analysis is conducted using R software version 4.4.

**Table 1:** Description of meteorological factors and daily number of cases of three intestinal infectious diseases in Shenzhen, China.

	MIN	P5	P50	P95	MAX
Daily cases of HFMD	0	3	73	392	861
Daily cases of DV	0	7	27	161	813
Daily cases of DN	3	11	37	115	226
Temperature(°C)	3.48	13.80	24.74	30.00	32.95
Relative humidity (%)	18.50	50.25	77.75	92.25	100.00
Barometric pressure (kPa)	99.06	100.25	101.29	102.32	103.53
Rainfall (mm/d)	0	0	0	44.00	485.00
Wind velocity (m/s)	0.25	1.00	1.75	3.50	6.50

MIN indicates the minimum value and MAX indicates the maximum value

## Results

During the 11-year surveillance period (2012−2022), Shenzhen’s municipal notifiable disease surveillance system documented a population-normalized incidence rate of 602.75 cases per 10^5^ population for this three infections. Stratified analysis revealed distinct epidemiological patterns: hand foot and mouth disease (HFMD) demonstrated the highest burden (337.59/10^5^), followed by non-viral diarrhea (DN, 132.7/10^5^) and diarrheal viruses (DV, 132.4/10^5^). Spearman correlation analysis (S1 Table in S1 File) revealed associations between temperature and daily new cases of HFMD (rs = 0.621), DV (rs = −0.345), and DN (rs = 0.231) with statistical significance at P = 0.01 level, indicating that temperature exerted the most pronounced influence on disease incidence.

**Fig 2:**
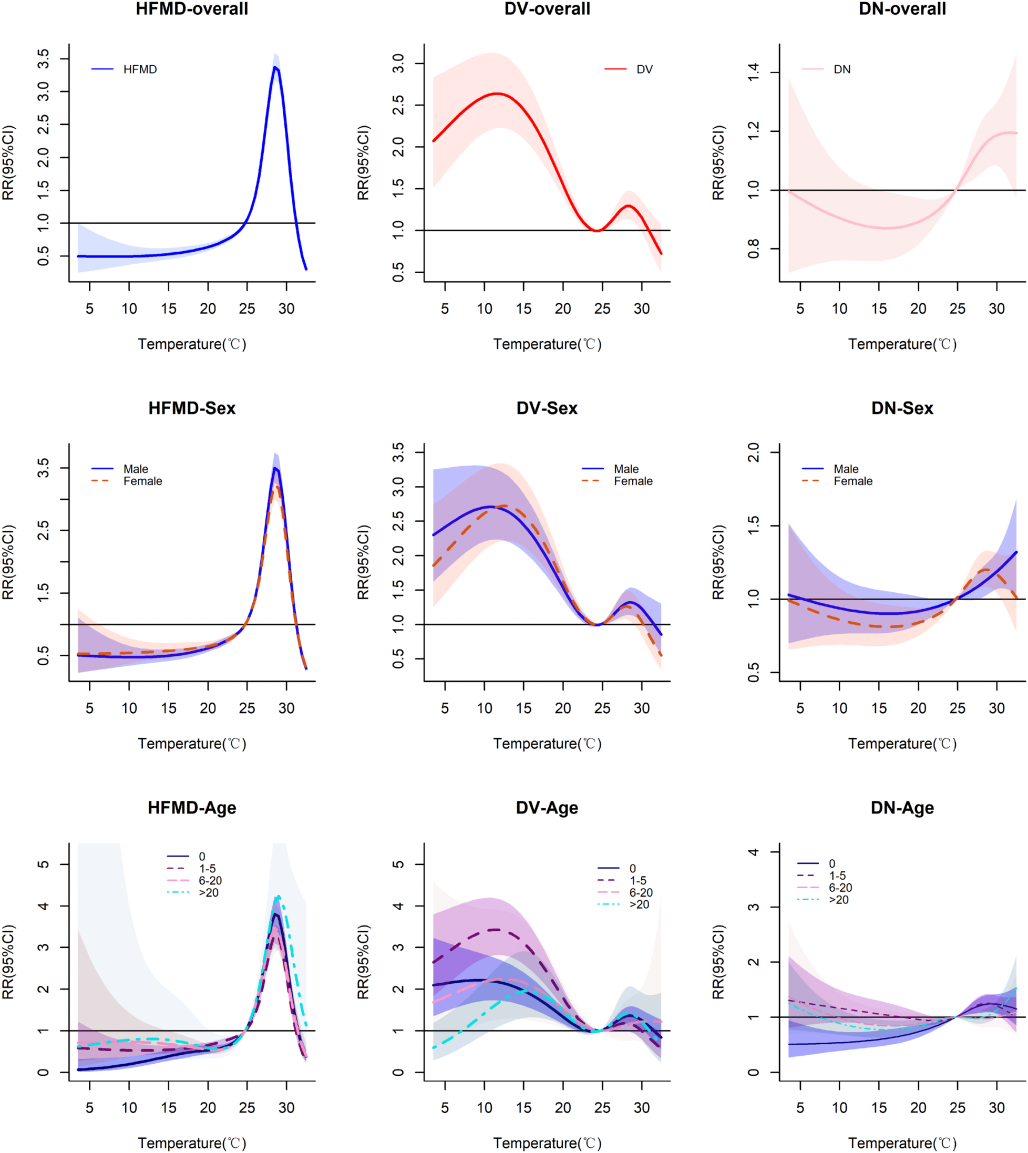
Temperature-dependent risk profiles of predominant intestinal infectious diseases across population subgroups. Each panel displays temperature-response relationships, with relative risk (RR) on the Y-axes. Solid curves represent mean risk estimates, bounded by 95% confidence intervals (shaded regions).

Fig 2 demonstrates a nonlinear association between temperature and disease risks: HFMD incidence rises at 25°C before declining beyond 30°C, while DV predominantly occurs below 25°C with transient susceptibility at 25–30°C. DN manifests detectable risks only above 25°C. Females mirrored male susceptibility patterns for HFMD and DV but exhibited attenuated DN risks at elevated temperatures. Age-stratified analyses revealed thermal thresholds of maximum vulnerability: adults (>20 years) demonstrated peak HFMD vulnerability (RR 4.24, 95%CI 2.95–6.09) at 29°C; preschoolers (1–5 years) showed highest DV risk (RR 3.43, 95%CI 2.82–4.19) at 11°C; exclusively adults (>20 years) sustained elevated DN risk (RR 1.61, 95%CI 1.11–2.34) when temperature exposures exceeded 30°C.

**Fig 3:**
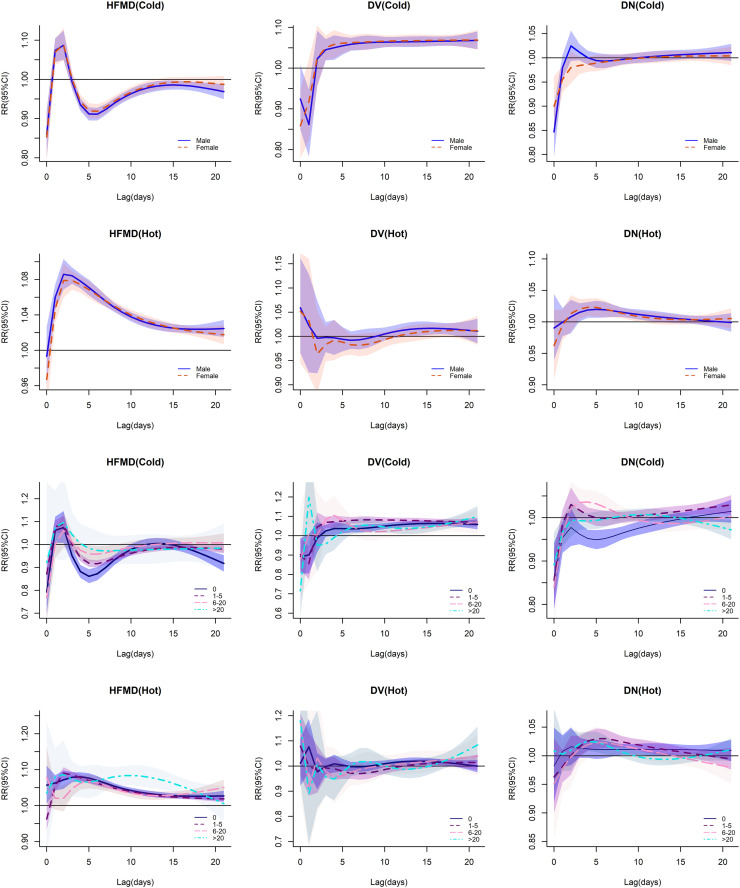
Exposure-dependent risk profiles of predominant intestinal infectious diseases across population subgroups. Each panel displays exposure-response relationships, with relative risk (RR) on the Y-axes. Solid curves represent mean risk estimates, bounded by 95% confidence intervals (shaded regions).

Extreme temperature lag analyses revealed distinct temporal susceptibility patterns: HFMD exhibited acute vulnerability to heat exposure (lag day 1) contrasted with cold-mediated protective effects emerging at lag day 4 (S2 Table in S1 File). DV demonstrated cold-induced pathogenesis initiating at lag day 3 versus delayed heat-associated risks (days 14–18). DN showed maximal heat-related pathogenicity concentrated between lag days 3–7 (Fig 3), with heat-associated relative risks demonstrating attenuated magnitudes across different disease phenotypes.

Gender-stratified analyses revealed differential thermal susceptibility: males demonstrated marginally greater cold-mediated protection (13.8°C) against HFMD than females during lag days 13–21, contrasting with non-significant heat-related risks. Male vulnerability to DV following sustained heat exposure (13–18 days) exceeded female counterparts. Both genders exhibited comparable heat-induced DN risks, though with distinct temporal windows (males: lag 3-12d; females: lag 3-10d), with no observable cold-protective effects (Fig 3 and S2 Table in S1 File).

Age-stratified analysis indicates that elevated temperatures exert a more significant influence on HFMD incidence in children aged ≤5 years, though these temperature-dependent variations attenuate after 3 consecutive days of heat exposure. While cooler temperatures initially confer protective benefits against DV, extended exposure consistently manifests adverse effects, particularly in the under-5 population. Notably, thermal effects exhibit age-specific temporal patterns: elevated temperatures enhance DV risk in neonates (0-year group) during days 11–17 post-exposure, whereas cooler temperatures reduce their DN incidence from lag days 3–11. Conversely, in children aged 1–5 years, sustained exposure to low temperatures for up to 14 days elevates DN risk. (Fig 3 and S2 Table in S1 File).

This study systematically evaluates population-wide health risks by analyzing the cumulative impact of 1°C temperature increments within non-extreme ranges. The analysis reveals immediate HFMD risk (0-day lag) at moderate temperatures (25–27°C). Notably, the high-temperature effect on HFMD incidence (25–29°C range) in adults (>20 years) demonstrates a 1–2 day delayed response. Population-level DV risk emerges consistently below 23°C, with the most rapid manifestation occurring at 17–21°C following 7-day exposure. Temperature elevation above 22°C reduces DV risk in individuals aged >5 years. Extended exposure (>4 consecutive days) to temperatures exceeding 25°C increases DN infection risk, with no gender-specific variations observed. Interestingly, children under 5 years demonstrate relative resistance to DN infection under extreme high-temperature conditions (S2 Table in S1 File).

**Fig 4:**
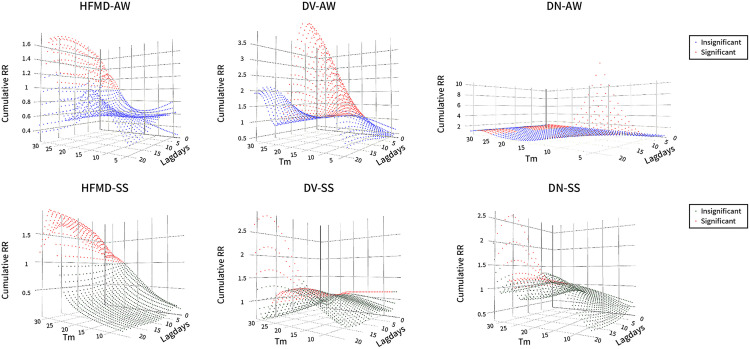
Cumulative risk of predominant intestinal infectious diseases in warm and cold seasons. SS represents spring and summer, AW represents autumn and winter; Tm stands for temperature (°C) and the Z-axis represents cumulative risk.

Fig 4 demonstrates distinct seasonal patterns in temperature-dependent disease risk profiles. For HFMD, incidence risk maintains consistent trends across 25–30°C in all seasons, yet shows significant seasonal variation above 30°C, with autumn and winter exhibiting reduced risk compared to spring and summer. DV outbreaks display seasonally constrained temperature thresholds, with spring and summer showing a narrower high-risk window (18–24.5°C) than autumn and winter. Notably, sustained temperatures exceeding 30°C continue to pose DV risks during warm seasons, unlike in colder periods. DN infection reveals bimodal risk intervals (25–27°C and >30°C) during warm seasons, while cold seasons demonstrate additional low-temperature-induced risks. Importantly, the cumulative risk escalation rate for cold-season DN infections accelerates with prolonged exposure duration.

**Fig 5:**
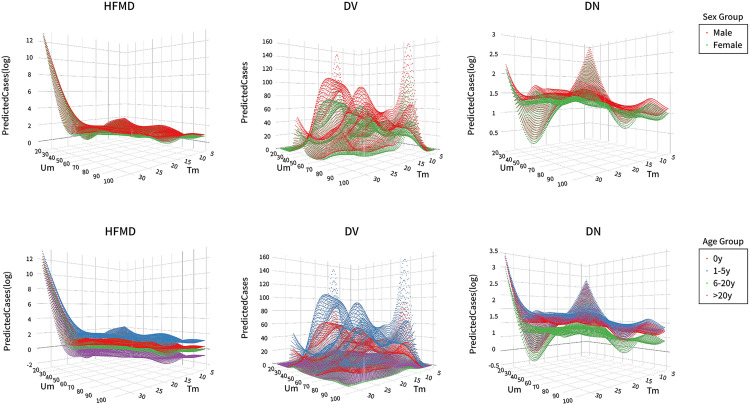
Prediction of case numbers across different age and gender groups under varying temperature and humidity conditions. Um represents humidity (%), Tm represents temperature (°C), and log represents the base ten logarithm of the number of cases.

Fig 5 reveals significant disease incidence changes in dependent temperature-humidity patterns. Male subjects demonstrate consistently higher susceptibility than females under equivalent environmental conditions. Among age groups, children aged 1–5 years show the greatest vulnerability to intestinal infections. Specific environmental thresholds emerge: HFMD incidence escalates markedly when temperatures surpass 23°C coupled with humidity levels below 50%. Conversely, DV cases increase substantially at high humidity (80%) with temperatures below 10°C. The interaction analysis indicates that low humidity (30%) begins to exert pronounced effects on DV transmission as temperatures rise from 15°C to 20°C. DN incidence peaks at both temperature extremes (high and low) under near-zero humidity conditions, while showing minimal humidity sensitivity within the optimal temperature range of 20–25°C.

Table 2 presents the performance metrics of the random forest-based early warning system for three infectious diseases, employing optimized ntree and mtry parameters identified through comprehensive sensitivity analysis (S2 Table in S1 File). The system demonstrated robust predictive capability for 2017–2018 incidence patterns, with notable variations observed across different diseases. Notably, for HFMD and DV infections, the P90 probability threshold achieved optimal performance, attaining perfect sensitivity (100%) while maintaining high specificity (>88%), reflected in superior Youden’s index values of 0.887 and 0.915, respectively. DV predictions showed particularly strong correlation with observed incidence patterns (R² = 0.888). In contrast, DN predictions demonstrated comparatively lower performance (R² = 0.702), with the P80 threshold providing the most balanced performance profile.

**Table 2:** Evaluation of the random forest model early warning system in 2012-2018.

	ntree	mtry	RMSE	*R* ^2^	P70			P80			P90		
					Sensitivity (%)	Specificity (%)	Youden’s index	Sensitivity (%)	Specificity (%)	Youden’s index	Sensitivity (%)	Specificity (%)	Youden’s index
HFMD	1000	3	525.344	0.821	87.50	96.92	0.844	88.89	96.15	0.851	100.00	88.76	0.887
DV	500	4	244.774	0.888	90.32	94.59	0.849	100.00	90.48	0.905	100.00	91.49	0.915
DN	500	4	59.059	0.702	79.27	73.91	0.532	70.69	89.36	0.601	76.67	80.00	0.567

P70, P80, and P90 represent the preset percentile threshold limits for historical cases.

**Fig 6:**
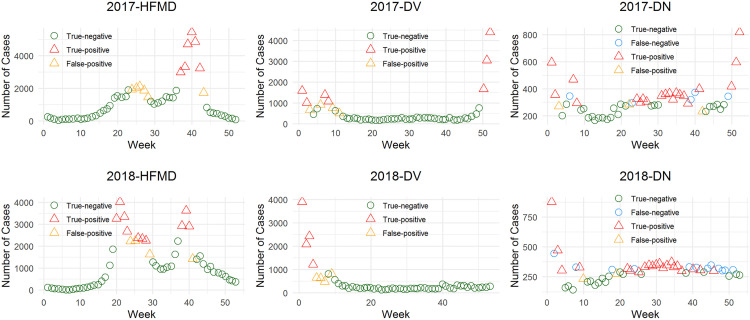
Prediction results of the early warning system.

Fig 6 demonstrates the early warning system’s prediction accuracy across three diseases during 2017–2018, with correct prediction rates of 90.48% (95/105) for HFMD, 92.38% (97/105) for DV, and 79.05% (83/105) for DN. HFMD alerts clustered predominantly in weeks 37–40, corresponding to late summer transmission peaks, while DV warnings concentrated in weeks 1–5, aligning with early-year outbreak patterns. In contrast, DN alerts showed year-round distribution with primary concentration between weeks 24–37, suggesting more persistent transmission dynamics.

## Discussion

Our research findings are consistent with previous studies [33], validating that among meteorological variables, temperature wields the most substantial influence on infection incidence, and this influence is regulated by humidity. Specifically, temperatures exceeding 25°C can heighten the prevalence of HFMD across the entire population. Conversely, extremely low temperatures exhibit a protective effect, suggesting a single – peak relationship between temperature and disease incidence. This is in contrast to the situation in Ningbo, China [34], where disease incidence peaks at both high and low temperatures, highlighting the regional disparities in the temperature-sensitivity of infectious diseases.

Regarding diarrheal diseases caused by different pathogens, there are distinct differences in temperature sensitivity between DV and DN. Similar to the research in Taiwan [25], DV is more prone to occur at lower temperatures, while DN poses a greater risk at higher temperatures. Differently from temperate regions, our study also uncovers a transient peak for DV within the temperature range of 25–30°C [33].

Temperature dynamically modulates infection transmission by altering pathogen survival and dissemination in the environment [35]. Studies reveal that low-temperature conditions markedly suppress the incidence of infectious intestinal diseases (IIDs) with shorter risk durations, whereas high-temperature-induced risks exhibit faster onset and prolonged persistence. For instance, the thermal effect of HFMD reveals immediate responsiveness, triggering transmission risks on the day of exposure and persisting for up to 21 days [30]. This temperature sensitivity may correlate with behavioral adaptations: reduced outdoor activities among children in cold environments lowers cross-infection risks [36]. Notably, temperature regulation exhibits pathogen-specific variability. Norovirus detection peaks on day 3 post low-temperature exposure, corresponding to a 3 day lag in DV risk manifestation [31].

Prolonged temperature exposure exerts nonlinear effects on pathogen transmission. Shenzhen data indicate that 30°C conditions rapidly amplify DN-induced infectious diarrhea risks within 7 days, though cumulative harm diminishes under sustained heating [37]. These findings suggest pathogen-specific thermal adaptation thresholds: transient heat shocks may enhance certain pathogens’ viability, while prolonged high temperatures disrupt their survival niches. Such divergent response mechanisms underscore the necessity for region-specific strategies in infectious disease control, emphasizing adaptive thresholds in pathogen-environment interactions.

Males in Guangdong exhibited higher susceptibility to infectious diarrhea, with cold exposure conferring HFMD protection while heatwaves amplified risks [38]. Children under 5 years from Zhejiang, China demonstrated minimal HFMD seropositivity [39], heightening infection vulnerability during temperature-favored viral replication. Interestingly, infants aged 0–2 years showed pronounced heat-associated diarrhea risks: Jiangsu’s <2-year cohort faced elevated diarrhea incidence during extreme heat [40], while Guangdong’s 0-year group developed diarrheal virus susceptibility on day 11 of thermal exposure as well. We found prolonged low-temperature exposure potentially induced DN-related risks in 1–5-year-olds, suggesting pathogen-specific thermal adaptation thresholds. These findings underscore the necessity for age-stratified interventions targeting regionally prevalent pathogens under distinct thermal stressors.

Identifying temperature ranges of risk is crucial for conducting comprehensive risk assessments and issuing timely warnings. Critical temperature thresholds govern infection risks, with HFMD exhibiting peak cumulative risk within 25–27°C, suggesting moderate heat requires prioritized surveillance over extremes [30]. Diarrheal viruses (DV), predominantly rotavirus and norovirus, demonstrate maximal viability at 20°C across multiple regions [41,42]. Gender disparities emerge as women show heightened diarrhea susceptibility during ≥7-day risk temperature stress periods. Moreover, elderly populations exhibit greater DN vulnerability compared to under-5 children under warming conditions, potentially linked to age-related physiological adaptations [43]. It advocates for demographic-specific risk stratification, emphasizing mid-range temperatures (20–27°C) as pivotal windows for targeted interventions against diarrhea.

Human activity seasonality in China modulates contact-driven IID transmission [44]. Shenzhen exhibits marked disparities in extreme temperature-associated incidence of predominant infectious intestinal diseases despite annual thermal stability. HFMD demonstrates greater high-temperature resilience in spring/summer versus autumn/winter, aligning with California coastal patterns linked to cold-season pathogen persistence [45]. DV outbreaks in spring/summer show narrowed cold-induced risk risk ranges, potentially mediated by humidity-enhanced viral inactivation [14]. Notably, seasonal stratification reveals divergent risk thresholds: spring/summer heat delays DV risks, while autumn/winter cold predisposes to DN infections. Crucially, diarrheal cases deviate from universal protective temperature range when seasonally stratified, evidencing humidity’s synergistic role with temperature in regulating intestinal infections [46]. Our results underscore the necessity for dual-parameter (temperature-humidity) modeling in climate-health risk assessments.

Our prediction models confirm demographic-specific vulnerability. HFMD exhibits sustained transmission risks at 28% relative humidity, reflecting viral adaptation to arid thermal environments (Fig 5) [47]. Seasonal variations may increase male susceptibility to HFMD under moderate-to-high temperatures and low humidity due to higher social activity frequency and prolonged exposure [30]. Shenzhen, characterized by a subtropical monsoon climate, experiences more pronounced humidity-related effects—exemplified by diarrhea surges in Surabaya during extreme aridity [48]. Reduced relative humidity enhances rotavirus survival and prolongs viral persistence, potentially elevating the risk of viral gastroenteritis among children under five across broader temperature ranges [38]. Children are more frequently infected by adults, and colder conditions promote indoor gatherings, thereby increasing diarrhea risk [42]. In contrast, non-viral gastroenteritis demonstrates minimal risk below 20–25°C regardless of humidity levels, providing a meteorological basis for differentiating diarrhea subtypes.

The weekly-scale models incorporating meteorological variables (temperature/humidity) and historical case data demonstrated robust predictive capacity for HFMD and DV, achieving explained variance R^2^ ≥ 0.82 and predictive accuracy exceeding 90% across validation set. For infections with distinct seasonal periodicity, the random forest algorithm outperformed comparative machine learning approaches including artificial neural networks (ANN) and support vector machines (SVM) when integrated with temporal feature engineering that accounted for autocorrelation effects [49]. However, the suboptimal model performance for DN (R^2^ = 0.702) revealed differential thermo-hydrological dependencies between bacterial and parasitic etiologies, with distinct climatic sensitivity profiles across pathogen types. Importantly, receiver operating characteristic analysis established that threshold optimization for early warning systems requires dual consideration of meteorological determinant precision and region-specific transmission baselines. These findings advocate for a dual-threshold decision framework enabling either sensitivity-prioritized early detection versus specificity-optimized resource allocation, contingent on distinct public health objectives.

This study has methodological constraints that warrant consideration. While population-level trends remain robust, our single-center design and 5-year forecast window necessitate validation through multicentric cohorts with extended surveillance to ensure epidemiological generalizability. Meanwhile, the demographic skew toward student populations in Shenzhen may confound occupational risk stratification – particularly for outdoor workers versus climate-controlled occupations. Subsequent study should incorporate standardized socio-occupational variables to disentangle profession-specific susceptibilities. These refinements could optimize climate-health early warning systems through heterogeneous population risk mapping.

## Conclusion

Our study provides important insights into the differential vulnerability of various demographic groups to extreme temperatures. Epidemiologically, Shenzhen’s predominant infectious intestinal diseases exhibited accelerated onset kinetics and prolonged epidemic durations during heat exposure compared to cold conditions. Notably, our research identified humidity-modulated seasonal variations in three predominant IID subtypes across equivalent thermal ranges, with predictive modeling indicating humidity-mediated modulation of these epidemiological patterns. These findings establish an evidence-based framework for developing meteorologically-informed alert systems, with predictive validation showing high accuracy in forecasting outbreak thresholds through temperature-humidity coupling algorithms.

## Supporting information

S1 File S1 Table. Descriptive result of correlation analysis between three kinds of intestinal infectious diseases and meteorological factors. S2 Table. Result of model analysis. S3 Fig. Epidemic profiles and model fitting.
(ZIP)

## Abbreviations:

IIDs: Intestinal infectious diseases
HFMD: Hand, foot, and mouth disease
DV: Viral infectious diarrhea
DN: Non-viral infectious diarrhea
AW: Autumn and Winter
Tm: Temperature
Um: Humidity
