# Supplementary material for: Temperature-humidity synergistic effects on predominant intestinal infectious diseases in Shenzhen, China: A predictive modeling framework for epidemiological early warning systems
Source: PLoS One. 2025 Dec 5;20(12):e0337929. doi: 10.1371/journal.pone.0337929 (PMC12680148; doi:10.1371/journal.pone.0337929)
Supplement: S1 File — S1 Table. Descriptive result of correlation analysis between three kinds of intestinal infectious diseases and meteorological factors. S2 Table. Result of model analysis. S3 Fig. Epidemic profiles and model fitting. (ZIP) [file pone.0337929.s001.zip › S2 Table.docx]

**Supplementary material**

**Model result**

**1.Independence lag effect**

| Table S2 Independent Delayed Day Effect of Gastrointestinal Infections in Extreme Temperatures | | | | | | |
| --- | --- | --- | --- | --- | --- | --- |
| Lag(days） | HFMD | | DV | | DN | |
|  | Low temperature(13.8℃) | High temperature(30.0℃) | Low temperature(13.8℃) | High temperature(30.0℃) | Low temperature(13.8℃) | High temperature(30.0℃) |
| lag0 | 0.859(0.809~0.913) | 0.977(0.948~1.007) | 0.917(0.849-0.991) | 1.053(0.968-1.146) | 0.892(0.823-0.966) | 1.008(0.939-1.083) |
| lag1 | 1.071(1.044~1.098) | 1.055(1.043~1.068) | 0.867(0.796-0.945) | 1.027(0.938-1.124) | 0.967(0.935-1) | 1.003(0.973-1.033) |
| lag2 | 1.085(1.052~1.119) | 1.085(1.07~1.101) | 1.027(0.968-1.089) | 0.984(0.919-1.054) | 0.995(0.956-1.035) | 1.01(0.974-1.046) |
| lag3 | 0.995(0.978~1.012) | 1.083(1.075~1.092) | 1.046(1.023-1.07) | 0.992(0.965-1.021) | 0.993(0.972-1.015) | 1.021(1.001-1.04) |
| lag4 | 0.938(0.924~0.952) | 1.077(1.069~1.084) | 1.05(1.023-1.078) | 0.995(0.963-1.029) | 0.992(0.974-1.011) | 1.025(1.009-1.042) |
| lag5 | 0.915(0.9~0.93) | 1.069(1.062~1.077) | 1.055(1.035-1.074) | 0.992(0.97-1.015) | 0.994(0.974-1.014) | 1.024(1.006-1.042) |
| lag6 | 0.914(0.9~0.928) | 1.062(1.055~1.069) | 1.058(1.044-1.072) | 0.989(0.973-1.006) | 0.997(0.978-1.016) | 1.019(1.003-1.036) |
| lag7 | 0.925(0.913~0.938) | 1.055(1.05~1.061) | 1.061(1.045-1.076) | 0.99(0.972-1.008) | 1.001(0.985-1.017) | 1.013(1-1.026) |
| lag8 | 0.941(0.93~0.952) | 1.049(1.044~1.053) | 1.062(1.047-1.078) | 0.992(0.975-1.011) | 1.004(0.991-1.018) | 1.007(0.997-1.017) |
| lag9 | 0.954(0.943~0.965) | 1.043(1.039~1.048) | 1.063(1.049-1.076) | 0.997(0.982-1.012) | 1.006(0.993-1.019) | 1.002(0.992-1.012) |
| lag10 | 0.964(0.953~0.976) | 1.039(1.034~1.043) | 1.063(1.052-1.074) | 1.001(0.989-1.013) | 1.007(0.994-1.021) | 0.998(0.988-1.009) |
| lag11 | 0.973(0.962~0.984) | 1.035(1.03~1.04) | 1.063(1.052-1.074) | 1.006(0.994-1.017) | 1.007(0.993-1.021) | 0.996(0.985-1.007) |
| lag12 | 0.979(0.968~0.991) | 1.032(1.027~1.037) | 1.063(1.052-1.075) | 1.009(0.996-1.022) | 1.006(0.992-1.021) | 0.994(0.983-1.005) |
| lag13 | 0.984(0.973~0.995) | 1.029(1.024~1.034) | 1.064(1.052-1.075) | 1.011(0.998-1.025) | 1.004(0.991-1.018) | 0.994(0.983-1.005) |
| lag14 | 0.987(0.976~0.998) | 1.027(1.022~1.031) | 1.064(1.052-1.076) | 1.013(1-1.027) | 1.002(0.989-1.015) | 0.994(0.984-1.004) |
| lag15 | 0.988(0.978~0.999) | 1.025(1.021~1.029) | 1.065(1.053-1.076) | 1.014(1.002-1.027) | 0.999(0.986-1.011) | 0.995(0.986-1.004) |
| lag16 | 0.988(0.978~0.998) | 1.024(1.02~1.028) | 1.065(1.055-1.076) | 1.014(1.003-1.026) | 0.995(0.983-1.007) | 0.997(0.988-1.005) |
| lag17 | 0.987(0.977~0.997) | 1.023(1.019~1.027) | 1.066(1.056-1.076) | 1.014(1.004-1.025) | 0.991(0.979-1.004) | 0.999(0.99-1.008) |
| lag18 | 0.985(0.975~0.996) | 1.023(1.018~1.027) | 1.066(1.056-1.077) | 1.014(1.002-1.025) | 0.987(0.973-1) | 1.002(0.991-1.012) |
| lag19 | 0.983(0.97~0.996) | 1.022(1.017~1.028) | 1.067(1.055-1.08) | 1.013(0.999-1.027) | 0.982(0.966-0.998) | 1.005(0.992-1.018) |
| lag20 | 0.98(0.965~0.995) | 1.022(1.015~1.029) | 1.068(1.052-1.084) | 1.012(0.994-1.03) | 0.977(0.959-0.996) | 1.008(0.992-1.024) |
| lag21 | 0.977(0.96~0.995) | 1.022(1.013~1.031) | 1.069(1.049-1.088) | 1.01(0.988-1.034) | 0.972(0.95-0.994) | 1.011(0.991-1.031) |

| Table S3 Gender-specific risk of HFMD in extreme temperature | | | | |
| --- | --- | --- | --- | --- |
| Lag(days） | Low temperature(13.8℃) | | High temperature(30.0℃) | |
|  | Male, RR(95%CI) | Female, RR(95%CI) | Male, RR(95%CI) | Female, RR(95%CI) |
| lag0 | 0.792(0.699-0.898) | 0.871(0.818-0.929) | 1.057(1.006-1.111) | 0.963(0.932-0.994) |
| lag1 | 1.063(1.007-1.122) | 1.078(1.049-1.106) | 1.063(1.042-1.084) | 1.056(1.042-1.07) |
| lag2 | 1.074(1.007-1.146) | 1.09(1.055-1.125) | 1.072(1.047-1.097) | 1.09(1.074-1.107) |
| lag3 | 0.951(0.917-0.985) | 0.999(0.982-1.017) | 1.079(1.065-1.093) | 1.086(1.077-1.095) |
| lag4 | 0.882(0.854-0.91) | 0.942(0.928-0.957) | 1.08(1.068-1.093) | 1.078(1.07-1.086) |
| lag5 | 0.861(0.831-0.892) | 0.918(0.902-0.934) | 1.077(1.064-1.09) | 1.07(1.061-1.078) |
| lag6 | 0.872(0.844-0.902) | 0.916(0.901-0.931) | 1.07(1.058-1.082) | 1.062(1.054-1.07) |
| lag7 | 0.9(0.875-0.926) | 0.926(0.913-0.939) | 1.062(1.053-1.072) | 1.055(1.049-1.061) |
| lag8 | 0.932(0.91-0.955) | 0.94(0.929-0.952) | 1.054(1.047-1.062) | 1.049(1.044-1.053) |
| lag9 | 0.958(0.936-0.981) | 0.952(0.941-0.964) | 1.048(1.04-1.055) | 1.043(1.038-1.048) |
| lag10 | 0.979(0.955-1.002) | 0.962(0.951-0.974) | 1.042(1.034-1.05) | 1.038(1.033-1.043) |
| lag11 | 0.993(0.969-1.018) | 0.971(0.959-0.983) | 1.038(1.029-1.046) | 1.034(1.029-1.04) |
| lag12 | 1.002(0.977-1.027) | 0.977(0.965-0.989) | 1.034(1.026-1.042) | 1.031(1.026-1.036) |
| lag13 | 1.005(0.981-1.03) | 0.982(0.97-0.993) | 1.031(1.023-1.039) | 1.028(1.023-1.033) |
| lag14 | 1.004(0.981-1.027) | 0.985(0.973-0.996) | 1.029(1.022-1.037) | 1.026(1.021-1.031) |
| lag15 | 0.998(0.977-1.02) | 0.987(0.976-0.997) | 1.028(1.021-1.035) | 1.024(1.02-1.029) |
| lag16 | 0.99(0.969-1.011) | 0.987(0.977-0.998) | 1.027(1.02-1.033) | 1.023(1.018-1.027) |
| lag17 | 0.978(0.957-1) | 0.987(0.977-0.998) | 1.026(1.019-1.033) | 1.021(1.017-1.026) |
| lag18 | 0.964(0.941-0.988) | 0.986(0.975-0.998) | 1.026(1.018-1.034) | 1.021(1.016-1.026) |
| lag19 | 0.949(0.923-0.976) | 0.985(0.971-0.998) | 1.026(1.017-1.036) | 1.02(1.014-1.026) |
| lag20 | 0.933(0.903-0.965) | 0.983(0.967-0.998) | 1.026(1.015-1.038) | 1.019(1.012-1.027) |
| lag21 | 0.917(0.882-0.954) | 0.98(0.962-0.999) | 1.027(1.013-1.041) | 1.019(1.01-1.028) |

| Table S4 Age-specific risk of HFMD in extreme temperature | | | | | | | | |
| --- | --- | --- | --- | --- | --- | --- | --- | --- |
| Lag(days） | Low temperature(13.8℃) | | | | High temperature(30.0℃) | | | |
|  | 0y, RR(95%CI) | 1-5y, RR(95%CI) | 6-20y, RR(95%CI) | >20y, RR(95%CI) | 0y, RR(95%CI) | 1-5y, RR(95%CI) | 6-20y, RR(95%CI) | >20y, RR(95%CI) |
| lag0 | 0.792(0.699-0.898) | 0.871(0.818-0.929) | 0.768(0.665-0.887) | 0.921(0.668-1.271) | 1.057(1.006-1.111) | 0.963(0.932-0.994) | 1.079(1-1.163) | 1.035(0.87-1.231) |
| lag1 | 1.063(1.007-1.122) | 1.078(1.049-1.106) | 1(0.942-1.063) | 1.076(0.937-1.235) | 1.063(1.042-1.084) | 1.056(1.042-1.07) | 1.02(0.989-1.052) | 1.08(1.006-1.161) |
| lag2 | 1.074(1.007-1.146) | 1.09(1.055-1.125) | 1.062(0.987-1.142) | 1.097(0.93-1.294) | 1.072(1.047-1.097) | 1.09(1.074-1.107) | 1.02(0.984-1.058) | 1.086(0.999-1.181) |
| lag3 | 0.951(0.917-0.985) | 0.999(0.982-1.017) | 1.011(0.972-1.052) | 1.044(0.953-1.143) | 1.079(1.065-1.093) | 1.086(1.077-1.095) | 1.047(1.027-1.068) | 1.073(1.025-1.123) |
| lag4 | 0.882(0.854-0.91) | 0.942(0.928-0.957) | 0.975(0.942-1.009) | 1.005(0.927-1.089) | 1.08(1.068-1.093) | 1.078(1.07-1.086) | 1.063(1.045-1.081) | 1.066(1.024-1.11) |
| lag5 | 0.861(0.831-0.892) | 0.918(0.902-0.934) | 0.959(0.923-0.996) | 0.983(0.899-1.075) | 1.077(1.064-1.09) | 1.07(1.061-1.078) | 1.067(1.048-1.087) | 1.065(1.02-1.113) |
| lag6 | 0.872(0.844-0.902) | 0.916(0.901-0.931) | 0.958(0.923-0.993) | 0.974(0.895-1.06) | 1.07(1.058-1.082) | 1.062(1.054-1.07) | 1.063(1.046-1.081) | 1.069(1.028-1.113) |
| lag7 | 0.9(0.875-0.926) | 0.926(0.913-0.939) | 0.964(0.935-0.994) | 0.972(0.904-1.046) | 1.062(1.053-1.072) | 1.055(1.049-1.061) | 1.055(1.041-1.069) | 1.075(1.042-1.109) |
| lag8 | 0.932(0.91-0.955) | 0.94(0.929-0.952) | 0.973(0.947-0.999) | 0.974(0.913-1.038) | 1.054(1.047-1.062) | 1.049(1.044-1.053) | 1.046(1.035-1.058) | 1.08(1.054-1.108) |
| lag9 | 0.958(0.936-0.981) | 0.952(0.941-0.964) | 0.981(0.956-1.007) | 0.975(0.915-1.038) | 1.048(1.04-1.055) | 1.043(1.038-1.048) | 1.04(1.029-1.05) | 1.083(1.057-1.109) |
| lag10 | 0.979(0.955-1.002) | 0.962(0.951-0.974) | 0.987(0.962-1.014) | 0.976(0.915-1.041) | 1.042(1.034-1.05) | 1.038(1.033-1.043) | 1.034(1.023-1.046) | 1.084(1.057-1.112) |
| lag11 | 0.993(0.969-1.018) | 0.971(0.959-0.983) | 0.993(0.966-1.02) | 0.977(0.914-1.044) | 1.038(1.029-1.046) | 1.034(1.029-1.04) | 1.031(1.019-1.043) | 1.083(1.054-1.112) |
| lag12 | 1.002(0.977-1.027) | 0.977(0.965-0.989) | 0.997(0.971-1.025) | 0.978(0.915-1.045) | 1.034(1.026-1.042) | 1.031(1.026-1.036) | 1.029(1.017-1.041) | 1.08(1.05-1.11) |
| lag13 | 1.005(0.981-1.03) | 0.982(0.97-0.993) | 1.001(0.975-1.028) | 0.979(0.917-1.045) | 1.031(1.023-1.039) | 1.028(1.023-1.033) | 1.028(1.016-1.04) | 1.075(1.047-1.104) |
| lag14 | 1.004(0.981-1.027) | 0.985(0.973-0.996) | 1.004(0.979-1.029) | 0.98(0.921-1.042) | 1.029(1.022-1.037) | 1.026(1.021-1.031) | 1.028(1.017-1.039) | 1.069(1.043-1.097) |
| lag15 | 0.998(0.977-1.02) | 0.987(0.976-0.997) | 1.005(0.982-1.03) | 0.98(0.924-1.04) | 1.028(1.021-1.035) | 1.024(1.02-1.029) | 1.029(1.019-1.04) | 1.062(1.038-1.087) |
| lag16 | 0.99(0.969-1.011) | 0.987(0.977-0.998) | 1.007(0.984-1.03) | 0.981(0.927-1.039) | 1.027(1.02-1.033) | 1.023(1.018-1.027) | 1.032(1.022-1.041) | 1.054(1.031-1.077) |
| lag17 | 0.978(0.957-1) | 0.987(0.977-0.998) | 1.007(0.984-1.031) | 0.982(0.926-1.041) | 1.026(1.019-1.033) | 1.021(1.017-1.026) | 1.034(1.024-1.044) | 1.045(1.022-1.069) |
| lag18 | 0.964(0.941-0.988) | 0.986(0.975-0.998) | 1.008(0.982-1.034) | 0.983(0.921-1.048) | 1.026(1.018-1.034) | 1.021(1.016-1.026) | 1.038(1.026-1.05) | 1.036(1.009-1.063) |
| lag19 | 0.949(0.923-0.976) | 0.985(0.971-0.998) | 1.008(0.979-1.038) | 0.983(0.913-1.059) | 1.026(1.017-1.036) | 1.02(1.014-1.026) | 1.042(1.027-1.056) | 1.026(0.993-1.059) |
| lag20 | 0.933(0.903-0.965) | 0.983(0.967-0.998) | 1.007(0.973-1.043) | 0.984(0.901-1.074) | 1.026(1.015-1.038) | 1.019(1.012-1.027) | 1.046(1.028-1.064) | 1.016(0.976-1.057) |
| lag21 | 0.917(0.882-0.954) | 0.98(0.962-0.999) | 1.007(0.967-1.049) | 0.985(0.887-1.092) | 1.027(1.013-1.041) | 1.019(1.01-1.028) | 1.05(1.028-1.073) | 1.005(0.957-1.056) |

| Table S5 Gender-specific risk of DV in extreme temperature | | | | |
| --- | --- | --- | --- | --- |
| Lag(days） | Low temperature(13.8℃) | | High temperature(30.0℃) | |
|  | Male, RR(95%CI) | Female, RR(95%CI) | Male, RR(95%CI) | Female, RR(95%CI) |
| lag0 | 0.924(0.849-1.007) | 0.859(0.779-0.946) | 1.059(0.966-1.162) | 1.052(0.945-1.172) |
| lag1 | 0.862(0.783-0.948) | 0.917(0.823-1.022) | 1.022(0.926-1.129) | 1.033(0.919-1.16) |
| lag2 | 1.023(0.958-1.092) | 1.025(0.952-1.105) | 0.996(0.924-1.074) | 0.964(0.883-1.052) |
| lag3 | 1.045(1.019-1.071) | 1.05(1.021-1.08) | 0.998(0.967-1.029) | 0.984(0.948-1.02) |
| lag4 | 1.05(1.02-1.081) | 1.058(1.024-1.093) | 0.997(0.962-1.034) | 0.992(0.951-1.035) |
| lag5 | 1.055(1.033-1.077) | 1.061(1.037-1.086) | 0.994(0.969-1.019) | 0.988(0.959-1.017) |
| lag6 | 1.059(1.043-1.074) | 1.063(1.045-1.08) | 0.992(0.974-1.01) | 0.983(0.962-1.004) |
| lag7 | 1.061(1.044-1.078) | 1.064(1.044-1.083) | 0.993(0.973-1.013) | 0.982(0.959-1.005) |
| lag8 | 1.063(1.046-1.08) | 1.065(1.045-1.084) | 0.996(0.976-1.016) | 0.984(0.961-1.007) |
| lag9 | 1.064(1.049-1.079) | 1.065(1.049-1.082) | 1(0.984-1.017) | 0.988(0.969-1.008) |
| lag10 | 1.064(1.051-1.077) | 1.066(1.052-1.08) | 1.005(0.992-1.018) | 0.993(0.978-1.009) |
| lag11 | 1.064(1.052-1.076) | 1.067(1.053-1.08) | 1.009(0.997-1.022) | 0.998(0.984-1.013) |
| lag12 | 1.065(1.052-1.077) | 1.067(1.053-1.081) | 1.012(0.999-1.026) | 1.003(0.987-1.019) |
| lag13 | 1.065(1.052-1.078) | 1.067(1.053-1.082) | 1.015(1-1.03) | 1.006(0.989-1.023) |
| lag14 | 1.065(1.052-1.078) | 1.068(1.053-1.083) | 1.016(1.001-1.031) | 1.008(0.991-1.026) |
| lag15 | 1.066(1.053-1.078) | 1.068(1.054-1.082) | 1.017(1.003-1.031) | 1.01(0.994-1.026) |
| lag16 | 1.066(1.054-1.078) | 1.068(1.055-1.082) | 1.017(1.004-1.029) | 1.011(0.997-1.026) |
| lag17 | 1.066(1.055-1.078) | 1.068(1.056-1.081) | 1.016(1.005-1.028) | 1.012(0.999-1.026) |
| lag18 | 1.067(1.055-1.079) | 1.069(1.055-1.082) | 1.015(1.003-1.028) | 1.012(0.998-1.027) |
| lag19 | 1.067(1.053-1.081) | 1.069(1.053-1.085) | 1.014(0.999-1.029) | 1.012(0.995-1.03) |
| lag20 | 1.068(1.05-1.085) | 1.069(1.049-1.089) | 1.012(0.993-1.033) | 1.012(0.989-1.036) |
| lag21 | 1.068(1.047-1.09) | 1.069(1.045-1.094) | 1.011(0.986-1.037) | 1.012(0.983-1.042) |

| Table S6 Age-specific risk of DV in extreme temperature | | | | | | | | |
| --- | --- | --- | --- | --- | --- | --- | --- | --- |
| Lag(days） | Low temperature(13.8℃) | | | | High temperature(30.0℃) | | | |
|  | 0y, RR(95%CI) | 1-5y, RR(95%CI) | 6-20y, RR(95%CI) | >20y, RR(95%CI) | 0y, RR(95%CI) | 1-5y, RR(95%CI) | 6-20y, RR(95%CI) | >20y, RR(95%CI) |
| lag0 | 0.892(0.807-0.987) | 0.903(0.824-0.988) | 0.878(0.677-1.137) | 0.716(0.588-0.872) | 1.012(0.922-1.11) | 1.079(0.962-1.21) | 1.176(0.883-1.568) | 1.18(0.933-1.493) |
| lag1 | 0.901(0.804-1.009) | 0.856(0.774-0.947) | 1.008(0.756-1.345) | 1.199(0.963-1.493) | 1.076(0.976-1.187) | 1(0.883-1.132) | 0.918(0.671-1.256) | 0.891(0.69-1.15) |
| lag2 | 0.988(0.914-1.069) | 1.046(0.976-1.121) | 1.005(0.823-1.226) | 0.951(0.817-1.108) | 0.977(0.908-1.052) | 0.983(0.894-1.082) | 1.031(0.814-1.308) | 1.007(0.831-1.22) |
| lag3 | 1.025(0.995-1.056) | 1.069(1.041-1.098) | 1.084(1.005-1.17) | 0.957(0.904-1.014) | 0.998(0.969-1.029) | 0.993(0.954-1.033) | 0.979(0.888-1.079) | 0.956(0.883-1.035) |
| lag4 | 1.037(1.002-1.074) | 1.072(1.041-1.105) | 1.105(1.012-1.206) | 0.991(0.927-1.06) | 1.009(0.974-1.045) | 0.99(0.945-1.036) | 0.955(0.852-1.072) | 0.948(0.864-1.041) |
| lag5 | 1.037(1.012-1.063) | 1.076(1.053-1.1) | 1.081(1.015-1.151) | 1.022(0.973-1.072) | 1.004(0.98-1.029) | 0.98(0.949-1.011) | 0.962(0.89-1.041) | 0.976(0.916-1.041) |
| lag6 | 1.036(1.017-1.054) | 1.079(1.063-1.096) | 1.051(1.005-1.098) | 1.042(1.007-1.079) | 0.998(0.98-1.016) | 0.972(0.95-0.994) | 0.976(0.923-1.032) | 1.005(0.959-1.053) |
| lag7 | 1.037(1.017-1.057) | 1.081(1.063-1.1) | 1.032(0.983-1.084) | 1.053(1.014-1.093) | 0.997(0.977-1.017) | 0.97(0.945-0.995) | 0.986(0.926-1.05) | 1.017(0.966-1.072) |
| lag8 | 1.04(1.02-1.061) | 1.082(1.064-1.101) | 1.023(0.974-1.074) | 1.054(1.015-1.095) | 0.999(0.98-1.019) | 0.973(0.949-0.998) | 0.994(0.934-1.057) | 1.018(0.967-1.071) |
| lag9 | 1.045(1.027-1.063) | 1.082(1.066-1.098) | 1.021(0.978-1.065) | 1.051(1.017-1.086) | 1.004(0.988-1.021) | 0.979(0.958-1) | 0.999(0.949-1.051) | 1.011(0.969-1.055) |
| lag10 | 1.05(1.035-1.065) | 1.082(1.068-1.095) | 1.022(0.986-1.06) | 1.045(1.017-1.075) | 1.009(0.996-1.023) | 0.986(0.97-1.003) | 1.002(0.963-1.044) | 1.001(0.967-1.035) |
| lag11 | 1.055(1.04-1.069) | 1.081(1.068-1.094) | 1.026(0.991-1.061) | 1.041(1.013-1.069) | 1.014(1.002-1.027) | 0.993(0.978-1.009) | 1.005(0.968-1.044) | 0.992(0.961-1.024) |
| lag12 | 1.058(1.043-1.073) | 1.08(1.067-1.093) | 1.03(0.994-1.067) | 1.038(1.01-1.068) | 1.017(1.004-1.031) | 0.999(0.982-1.016) | 1.008(0.967-1.051) | 0.988(0.954-1.023) |
| lag13 | 1.061(1.045-1.077) | 1.079(1.065-1.093) | 1.034(0.996-1.073) | 1.039(1.009-1.069) | 1.019(1.005-1.034) | 1.004(0.986-1.022) | 1.012(0.968-1.058) | 0.988(0.952-1.025) |
| lag14 | 1.063(1.047-1.078) | 1.078(1.064-1.092) | 1.038(1-1.077) | 1.041(1.012-1.072) | 1.02(1.005-1.034) | 1.008(0.989-1.026) | 1.015(0.971-1.061) | 0.991(0.955-1.029) |
| lag15 | 1.063(1.049-1.078) | 1.077(1.064-1.09) | 1.042(1.006-1.08) | 1.046(1.017-1.075) | 1.019(1.005-1.033) | 1.01(0.993-1.028) | 1.018(0.977-1.061) | 0.998(0.964-1.033) |
| lag16 | 1.064(1.05-1.078) | 1.076(1.064-1.088) | 1.047(1.013-1.082) | 1.052(1.025-1.079) | 1.017(1.005-1.03) | 1.012(0.997-1.028) | 1.022(0.984-1.06) | 1.008(0.977-1.04) |
| lag17 | 1.063(1.05-1.077) | 1.075(1.063-1.087) | 1.052(1.018-1.086) | 1.059(1.033-1.086) | 1.015(1.003-1.026) | 1.013(0.999-1.028) | 1.025(0.991-1.061) | 1.02(0.99-1.05) |
| lag18 | 1.063(1.048-1.077) | 1.074(1.061-1.086) | 1.056(1.021-1.093) | 1.068(1.04-1.097) | 1.011(0.999-1.024) | 1.014(0.999-1.03) | 1.029(0.991-1.067) | 1.034(1.002-1.067) |
| lag19 | 1.062(1.045-1.079) | 1.073(1.058-1.087) | 1.061(1.02-1.104) | 1.078(1.045-1.113) | 1.008(0.993-1.023) | 1.014(0.995-1.034) | 1.032(0.985-1.081) | 1.049(1.009-1.091) |
| lag20 | 1.06(1.04-1.081) | 1.071(1.053-1.09) | 1.066(1.015-1.12) | 1.089(1.047-1.132) | 1.004(0.984-1.024) | 1.014(0.99-1.039) | 1.036(0.974-1.101) | 1.066(1.013-1.122) |
| lag21 | 1.059(1.033-1.085) | 1.07(1.048-1.093) | 1.071(1.008-1.138) | 1.1(1.048-1.154) | 1(0.975-1.025) | 1.014(0.983-1.046) | 1.039(0.961-1.124) | 1.084(1.015-1.157) |

| Table S7 Gender-specific risk of DN in extreme temperature | | | | |
| --- | --- | --- | --- | --- |
| Lag(days） | Low temperature(13.8℃) | | High temperature(30.0℃) | |
|  | Male, RR(95%CI) | Female, RR(95%CI) | Male, RR(95%CI) | Female, RR(95%CI) |
| lag0 | 0.847(0.796-0.901) | 0.9(0.842-0.962) | 0.99(0.94-1.044) | 0.962(0.909-1.019) |
| lag1 | 0.98(0.955-1.005) | 0.956(0.929-0.983) | 0.999(0.977-1.022) | 0.995(0.971-1.019) |
| lag2 | 1.025(0.994-1.056) | 0.98(0.948-1.013) | 1.008(0.982-1.035) | 1.013(0.985-1.042) |
| lag3 | 1.012(0.995-1.029) | 0.984(0.966-1.002) | 1.015(1.001-1.03) | 1.021(1.005-1.036) |
| lag4 | 1(0.986-1.015) | 0.987(0.971-1.002) | 1.019(1.006-1.031) | 1.023(1.01-1.037) |
| lag5 | 0.995(0.979-1.01) | 0.989(0.972-1.006) | 1.02(1.006-1.034) | 1.023(1.008-1.038) |
| lag6 | 0.993(0.979-1.008) | 0.991(0.976-1.007) | 1.019(1.007-1.032) | 1.021(1.007-1.034) |
| lag7 | 0.994(0.982-1.007) | 0.994(0.981-1.007) | 1.018(1.008-1.027) | 1.017(1.007-1.028) |
| lag8 | 0.996(0.986-1.007) | 0.996(0.985-1.007) | 1.016(1.008-1.023) | 1.014(1.006-1.022) |
| lag9 | 0.998(0.988-1.009) | 0.998(0.987-1.009) | 1.014(1.006-1.021) | 1.011(1.003-1.019) |
| lag10 | 1(0.99-1.011) | 0.999(0.988-1.011) | 1.012(1.004-1.02) | 1.009(1-1.017) |
| lag11 | 1.002(0.991-1.013) | 1(0.989-1.012) | 1.01(1.002-1.019) | 1.007(0.998-1.016) |
| lag12 | 1.003(0.992-1.014) | 1.002(0.99-1.013) | 1.009(1-1.017) | 1.006(0.997-1.015) |
| lag13 | 1.004(0.994-1.015) | 1.002(0.991-1.014) | 1.007(0.999-1.016) | 1.005(0.996-1.013) |
| lag14 | 1.005(0.995-1.016) | 1.003(0.992-1.014) | 1.006(0.998-1.014) | 1.004(0.996-1.012) |
| lag15 | 1.006(0.997-1.016) | 1.003(0.993-1.014) | 1.005(0.998-1.012) | 1.004(0.996-1.011) |
| lag16 | 1.007(0.998-1.017) | 1.004(0.993-1.014) | 1.004(0.997-1.01) | 1.003(0.997-1.01) |
| lag17 | 1.008(0.998-1.018) | 1.004(0.993-1.015) | 1.003(0.996-1.009) | 1.004(0.997-1.011) |
| lag18 | 1.009(0.998-1.02) | 1.004(0.992-1.016) | 1.002(0.994-1.01) | 1.004(0.996-1.012) |
| lag19 | 1.01(0.997-1.022) | 1.004(0.99-1.018) | 1.001(0.991-1.011) | 1.004(0.994-1.015) |
| lag20 | 1.01(0.995-1.025) | 1.004(0.988-1.02) | 1(0.988-1.012) | 1.005(0.992-1.018) |
| lag21 | 1.011(0.993-1.029) | 1.004(0.985-1.023) | 0.999(0.984-1.014) | 1.005(0.989-1.022) |

| Table S8 Age-specific risk of DN in extreme temperature | | | | | | | | |
| --- | --- | --- | --- | --- | --- | --- | --- | --- |
| Lag(days） | Low temperature(13.8℃) | | | | High temperature(30.0℃) | | | |
|  | 0y, RR(95%CI) | 1-5y, RR(95%CI) | 6-20y, RR(95%CI) | >20y, RR(95%CI) | 0y, RR(95%CI) | 1-5y, RR(95%CI) | 6-20y, RR(95%CI) | >20y, RR(95%CI) |
| lag0 | 0.863(0.79-0.943) | 0.856(0.793-0.924) | 0.839(0.749-0.94) | 0.892(0.823-0.966) | 0.983(0.92-1.049) | 0.963(0.9-1.031) | 0.951(0.855-1.057) | 1.008(0.939-1.083) |
| lag1 | 0.954(0.919-0.99) | 0.987(0.956-1.018) | 0.962(0.917-1.008) | 0.967(0.935-1) | 1.008(0.981-1.036) | 0.981(0.954-1.01) | 0.977(0.935-1.022) | 1.003(0.973-1.033) |
| lag2 | 0.978(0.935-1.022) | 1.03(0.992-1.07) | 1.022(0.967-1.081) | 0.995(0.956-1.035) | 1.016(0.984-1.049) | 1(0.967-1.035) | 0.998(0.946-1.052) | 1.01(0.974-1.046) |
| lag3 | 0.962(0.939-0.986) | 1.018(0.997-1.039) | 1.035(1.004-1.067) | 0.993(0.972-1.015) | 1.014(0.996-1.032) | 1.016(0.998-1.035) | 1.012(0.983-1.041) | 1.021(1.001-1.04) |
| lag4 | 0.952(0.932-0.972) | 1.006(0.989-1.024) | 1.036(1.01-1.064) | 0.992(0.974-1.011) | 1.012(0.997-1.028) | 1.026(1.009-1.042) | 1.02(0.995-1.045) | 1.025(1.009-1.042) |
| lag5 | 0.949(0.928-0.972) | 1(0.981-1.019) | 1.032(1.003-1.062) | 0.994(0.974-1.014) | 1.011(0.994-1.028) | 1.03(1.012-1.048) | 1.023(0.996-1.05) | 1.024(1.006-1.042) |
| lag6 | 0.952(0.931-0.973) | 0.998(0.98-1.016) | 1.024(0.997-1.052) | 0.997(0.978-1.016) | 1.011(0.996-1.026) | 1.03(1.014-1.046) | 1.023(0.999-1.048) | 1.019(1.003-1.036) |
| lag7 | 0.958(0.94-0.975) | 0.999(0.984-1.014) | 1.015(0.993-1.038) | 1.001(0.985-1.017) | 1.011(0.999-1.023) | 1.028(1.015-1.04) | 1.021(1.002-1.04) | 1.013(1-1.026) |
| lag8 | 0.964(0.949-0.979) | 1.001(0.988-1.014) | 1.007(0.987-1.026) | 1.004(0.991-1.018) | 1.011(1.001-1.021) | 1.025(1.015-1.035) | 1.018(1.003-1.033) | 1.007(0.997-1.017) |
| lag9 | 0.97(0.956-0.985) | 1.002(0.99-1.015) | 1(0.981-1.019) | 1.006(0.993-1.019) | 1.011(1.002-1.021) | 1.022(1.012-1.031) | 1.015(1.001-1.03) | 1.002(0.992-1.012) |
| lag10 | 0.976(0.961-0.991) | 1.004(0.991-1.017) | 0.995(0.976-1.014) | 1.007(0.994-1.021) | 1.011(1.001-1.021) | 1.019(1.009-1.029) | 1.012(0.997-1.028) | 0.998(0.988-1.009) |
| lag11 | 0.981(0.965-0.997) | 1.006(0.993-1.02) | 0.991(0.971-1.011) | 1.007(0.993-1.021) | 1.011(1.001-1.022) | 1.016(1.005-1.027) | 1.009(0.993-1.026) | 0.996(0.985-1.007) |
| lag12 | 0.986(0.97-1.002) | 1.008(0.995-1.022) | 0.988(0.968-1.008) | 1.006(0.992-1.021) | 1.011(1.001-1.022) | 1.014(1.003-1.025) | 1.006(0.99-1.023) | 0.994(0.983-1.005) |
| lag13 | 0.99(0.974-1.006) | 1.011(0.997-1.024) | 0.987(0.968-1.007) | 1.004(0.991-1.018) | 1.011(1.001-1.022) | 1.011(1.001-1.022) | 1.003(0.987-1.019) | 0.994(0.983-1.005) |
| lag14 | 0.994(0.979-1.009) | 1.013(1-1.026) | 0.987(0.968-1.006) | 1.002(0.989-1.015) | 1.011(1.001-1.021) | 1.009(0.999-1.019) | 1(0.985-1.015) | 0.994(0.984-1.004) |
| lag15 | 0.997(0.983-1.012) | 1.015(1.003-1.027) | 0.987(0.97-1.005) | 0.999(0.986-1.011) | 1.011(1.002-1.02) | 1.007(0.998-1.016) | 0.997(0.984-1.011) | 0.995(0.986-1.004) |
| lag16 | 1.001(0.987-1.015) | 1.017(1.006-1.029) | 0.989(0.971-1.006) | 0.995(0.983-1.007) | 1.011(1.002-1.019) | 1.005(0.996-1.013) | 0.994(0.982-1.007) | 0.997(0.988-1.005) |
| lag17 | 1.004(0.989-1.018) | 1.02(1.008-1.032) | 0.991(0.973-1.009) | 0.991(0.979-1.004) | 1.01(1.002-1.019) | 1.003(0.994-1.011) | 0.991(0.979-1.004) | 0.999(0.99-1.008) |
| lag18 | 1.007(0.991-1.023) | 1.022(1.009-1.036) | 0.993(0.974-1.013) | 0.987(0.973-1) | 1.01(1-1.02) | 1.001(0.991-1.011) | 0.988(0.973-1.003) | 1.002(0.991-1.012) |
| lag19 | 1.009(0.991-1.028) | 1.025(1.009-1.04) | 0.996(0.974-1.019) | 0.982(0.966-0.998) | 1.01(0.998-1.022) | 0.999(0.986-1.011) | 0.985(0.967-1.004) | 1.005(0.992-1.018) |
| lag20 | 1.012(0.99-1.035) | 1.027(1.008-1.046) | 0.999(0.973-1.027) | 0.977(0.959-0.996) | 1.009(0.994-1.025) | 0.997(0.981-1.012) | 0.982(0.959-1.006) | 1.008(0.992-1.024) |
| lag21 | 1.015(0.989-1.041) | 1.029(1.008-1.052) | 1.003(0.972-1.035) | 0.972(0.95-0.994) | 1.009(0.991-1.028) | 0.995(0.976-1.014) | 0.979(0.951-1.009) | 1.011(0.991-1.031) |

**2.Cumulative lag effect**

|  | Table S9 Risk of HFMD with each 1 °C change in lag time from 14 °C to 30 °C | | | | | | | | | | | | | | | | |
| --- | --- | --- | --- | --- | --- | --- | --- | --- | --- | --- | --- | --- | --- | --- | --- | --- | --- |
| Lag(days) | Temperature(℃), RR(95%CI) | | | | | | | | | | | | | | | | |
|  | 14 | 15 | 16 | 17 | 18 | 19 | 20 | 21 | 22 | 23 | 24 | 25 | 26 | 27 | 28 | 29 | 30 |
| lag0 | 0.862(0.812-0.915) | 0.876(0.829-0.925) | 0.889(0.845-0.935) | 0.902(0.861-0.945) | 0.914(0.877-0.953) | 0.926(0.893-0.96) | 0.938(0.91-0.966) | 0.949(0.925-0.974) | 0.961(0.941-0.982) | 0.975(0.96-0.991) | 0.99(0.983-0.997) | 1.003(1.001-1.006)* | 1.014(1.003-1.024)* | 1.019(1.002-1.035)* | 1.015(0.993-1.039) | 1.002(0.975-1.029) | 0.977(0.948-1.007) |
| lag1 | 0.921(0.867-0.979) | 0.926(0.876-0.98) | 0.931(0.884-0.981) | 0.936(0.892-0.982) | 0.94(0.9-0.981) | 0.943(0.909-0.979) | 0.947(0.918-0.976) | 0.951(0.926-0.977) | 0.959(0.937-0.981) | 0.97(0.953-0.987) | 0.985(0.978-0.993) | 1.006(1.003-1.008) | 1.03(1.019-1.042) | 1.058(1.04-1.076) | 1.078(1.052-1.104)* | 1.071(1.041-1.102)* | 1.031(1-1.064)* |
| lag2 | 0.997(0.939-1.058) | 0.989(0.935-1.046) | 0.981(0.931-1.034) | 0.974(0.928-1.023) | 0.967(0.925-1.01) | 0.96(0.924-0.996) | 0.954(0.924-0.985) | 0.951(0.925-0.978) | 0.953(0.93-0.977) | 0.962(0.944-0.98) | 0.98(0.971-0.988) | 1.009(1.006-1.012) | 1.053(1.041-1.066) | 1.115(1.095-1.134) | 1.17(1.142-1.199) | 1.177(1.144-1.212) | 1.119(1.086-1.154) |
| lag3 | 0.99(0.927-1.057) | 0.977(0.919-1.039) | 0.965(0.911-1.022) | 0.954(0.904-1.006) | 0.943(0.899-0.989) | 0.934(0.896-0.973) | 0.927(0.895-0.959) | 0.924(0.897-0.952) | 0.928(0.904-0.954) | 0.942(0.923-0.962) | 0.969(0.96-0.978) | 1.014(1.011-1.017) | 1.083(1.07-1.097) | 1.183(1.162-1.206) | 1.279(1.246-1.312) | 1.299(1.261-1.338) | 1.212(1.175-1.251) |
| lag4 | 0.929(0.865-0.997) | 0.917(0.858-0.979) | 0.906(0.852-0.964) | 0.897(0.847-0.95) | 0.889(0.845-0.936) | 0.883(0.845-0.922) | 0.88(0.848-0.913) | 0.882(0.855-0.911) | 0.894(0.868-0.919) | 0.917(0.897-0.937) | 0.956(0.947-0.966) | 1.019(1.016-1.023) | 1.117(1.102-1.132) | 1.259(1.235-1.284) | 1.398(1.362-1.436) | 1.43(1.386-1.474) | 1.305(1.264-1.348) |
| lag5 | 0.85(0.786-0.919) | 0.842(0.783-0.905) | 0.835(0.78-0.894) | 0.83(0.78-0.884) | 0.827(0.782-0.875) | 0.826(0.788-0.866) | 0.829(0.796-0.862) | 0.838(0.809-0.867) | 0.857(0.831-0.884) | 0.89(0.869-0.911) | 0.943(0.933-0.953) | 1.025(1.021-1.029) | 1.152(1.135-1.168) | 1.338(1.311-1.366) | 1.523(1.481-1.567) | 1.564(1.515-1.616) | 1.396(1.35-1.444) |
| lag6 | 0.778(0.713-0.848) | 0.773(0.714-0.838) | 0.771(0.715-0.831) | 0.77(0.718-0.825) | 0.771(0.725-0.82) | 0.775(0.735-0.816) | 0.782(0.749-0.817) | 0.797(0.768-0.828) | 0.824(0.796-0.852) | 0.865(0.843-0.888) | 0.93(0.919-0.941) | 1.03(1.026-1.034) | 1.185(1.166-1.203) | 1.415(1.384-1.447) | 1.649(1.6-1.699) | 1.699(1.641-1.759) | 1.483(1.43-1.537) |
| lag7 | 0.72(0.655-0.792) | 0.719(0.658-0.785) | 0.72(0.663-0.781) | 0.722(0.669-0.779) | 0.727(0.679-0.778) | 0.734(0.693-0.777) | 0.745(0.711-0.781) | 0.765(0.734-0.797) | 0.796(0.768-0.826) | 0.845(0.822-0.869) | 0.92(0.908-0.932) | 1.035(1.031-1.039) | 1.215(1.194-1.235) | 1.488(1.453-1.524) | 1.771(1.714-1.829) | 1.831(1.764-1.9) | 1.564(1.505-1.626) |
| lag8 | 0.678(0.612-0.751) | 0.679(0.617-0.747) | 0.682(0.624-0.745) | 0.686(0.632-0.745) | 0.693(0.645-0.746) | 0.703(0.661-0.747) | 0.717(0.681-0.754) | 0.739(0.708-0.773) | 0.775(0.745-0.806) | 0.829(0.804-0.854) | 0.911(0.899-0.924) | 1.039(1.034-1.044) | 1.241(1.219-1.264) | 1.556(1.517-1.596) | 1.888(1.824-1.953) | 1.957(1.882-2.035) | 1.641(1.576-1.708) |
| lag9 | 0.647(0.58-0.721) | 0.649(0.587-0.719) | 0.654(0.595-0.719) | 0.66(0.605-0.72) | 0.669(0.619-0.722) | 0.68(0.637-0.726) | 0.696(0.659-0.734) | 0.72(0.687-0.754) | 0.758(0.727-0.79) | 0.816(0.79-0.842) | 0.904(0.891-0.917) | 1.042(1.037-1.047) | 1.265(1.241-1.289) | 1.618(1.576-1.662) | 1.999(1.928-2.072) | 2.078(1.996-2.165) | 1.712(1.641-1.785) |
| lag10 | 0.624(0.556-0.7) | 0.628(0.564-0.699) | 0.633(0.573-0.7) | 0.641(0.584-0.702) | 0.65(0.599-0.706) | 0.663(0.619-0.71) | 0.68(0.642-0.72) | 0.706(0.672-0.741) | 0.746(0.714-0.779) | 0.806(0.779-0.833) | 0.898(0.885-0.912) | 1.045(1.04-1.05) | 1.286(1.26-1.311) | 1.676(1.63-1.723) | 2.104(2.027-2.184) | 2.194(2.104-2.289) | 1.778(1.702-1.857) |
| lag11 | 0.607(0.538-0.686) | 0.612(0.546-0.685) | 0.618(0.556-0.687) | 0.627(0.569-0.69) | 0.637(0.585-0.694) | 0.65(0.605-0.699) | 0.668(0.629-0.71) | 0.695(0.66-0.732) | 0.736(0.703-0.771) | 0.798(0.771-0.827) | 0.894(0.88-0.908) | 1.048(1.042-1.053) | 1.304(1.277-1.331) | 1.729(1.679-1.779) | 2.205(2.121-2.292) | 2.305(2.206-2.408) | 1.84(1.759-1.924) |
| lag12 | 0.595(0.523-0.676) | 0.6(0.533-0.676) | 0.607(0.544-0.679) | 0.616(0.557-0.682) | 0.628(0.574-0.687) | 0.641(0.594-0.692) | 0.66(0.62-0.703) | 0.688(0.651-0.726) | 0.73(0.695-0.766) | 0.793(0.764-0.823) | 0.89(0.876-0.905) | 1.05(1.044-1.055) | 1.32(1.291-1.349) | 1.778(1.725-1.832) | 2.301(2.209-2.396) | 2.412(2.305-2.524) | 1.898(1.811-1.989) |
| lag13 | 0.586(0.512-0.67) | 0.592(0.522-0.67) | 0.599(0.534-0.673) | 0.609(0.547-0.677) | 0.621(0.565-0.682) | 0.635(0.586-0.688) | 0.654(0.612-0.699) | 0.682(0.644-0.723) | 0.725(0.689-0.763) | 0.789(0.759-0.82) | 0.888(0.872-0.903) | 1.051(1.045-1.057) | 1.334(1.304-1.364) | 1.823(1.767-1.881) | 2.393(2.294-2.496) | 2.516(2.399-2.638) | 1.953(1.86-2.05) |
| lag14 | 0.578(0.502-0.665) | 0.585(0.513-0.666) | 0.593(0.525-0.669) | 0.603(0.54-0.674) | 0.615(0.558-0.679) | 0.631(0.58-0.686) | 0.65(0.607-0.697) | 0.679(0.639-0.721) | 0.722(0.685-0.762) | 0.786(0.755-0.819) | 0.886(0.87-0.902) | 1.052(1.046-1.059) | 1.346(1.314-1.378) | 1.866(1.805-1.928) | 2.482(2.375-2.594) | 2.617(2.49-2.749) | 2.006(1.907-2.11) |
| lag15 | 0.571(0.494-0.661) | 0.578(0.505-0.662) | 0.587(0.518-0.666) | 0.598(0.533-0.672) | 0.611(0.551-0.678) | 0.627(0.575-0.684) | 0.648(0.602-0.697) | 0.677(0.636-0.721) | 0.721(0.681-0.762) | 0.785(0.752-0.819) | 0.884(0.867-0.902) | 1.053(1.047-1.06) | 1.357(1.324-1.391) | 1.906(1.842-1.972) | 2.569(2.454-2.689) | 2.716(2.58-2.859) | 2.056(1.951-2.167) |
| lag16 | 0.565(0.485-0.657) | 0.572(0.497-0.659) | 0.582(0.511-0.663) | 0.593(0.526-0.669) | 0.607(0.546-0.676) | 0.624(0.57-0.684) | 0.646(0.598-0.697) | 0.676(0.633-0.722) | 0.72(0.679-0.763) | 0.784(0.75-0.82) | 0.883(0.866-0.901) | 1.054(1.047-1.061) | 1.367(1.332-1.402) | 1.944(1.876-2.014) | 2.655(2.531-2.784) | 2.815(2.669-2.969) | 2.106(1.994-2.224) |
| lag17 | 0.558(0.477-0.652) | 0.566(0.489-0.654) | 0.576(0.503-0.659) | 0.589(0.52-0.666) | 0.604(0.54-0.674) | 0.621(0.566-0.683) | 0.644(0.595-0.697) | 0.676(0.631-0.724) | 0.72(0.678-0.765) | 0.784(0.749-0.822) | 0.883(0.865-0.902) | 1.055(1.048-1.062) | 1.376(1.339-1.413) | 1.981(1.909-2.055) | 2.739(2.608-2.877) | 2.914(2.758-3.078) | 2.155(2.037-2.28) |
| lag18 | 0.55(0.468-0.646) | 0.559(0.481-0.649) | 0.57(0.496-0.654) | 0.583(0.513-0.662) | 0.599(0.535-0.671) | 0.618(0.561-0.681) | 0.643(0.592-0.698) | 0.676(0.629-0.726) | 0.721(0.677-0.768) | 0.785(0.748-0.824) | 0.883(0.864-0.902) | 1.056(1.048-1.063) | 1.384(1.346-1.422) | 2.016(1.942-2.094) | 2.824(2.686-2.971) | 3.014(2.848-3.189) | 2.204(2.079-2.336) |
| lag19 | 0.54(0.458-0.638) | 0.55(0.472-0.641) | 0.562(0.487-0.648) | 0.576(0.506-0.657) | 0.594(0.528-0.668) | 0.615(0.556-0.68) | 0.641(0.589-0.698) | 0.676(0.627-0.728) | 0.722(0.676-0.771) | 0.786(0.748-0.827) | 0.883(0.863-0.903) | 1.056(1.048-1.064) | 1.391(1.352-1.431) | 2.051(1.973-2.133) | 2.91(2.763-3.065) | 3.115(2.939-3.302) | 2.253(2.121-2.393) |
| lag20 | 0.53(0.447-0.629) | 0.54(0.461-0.633) | 0.553(0.478-0.641) | 0.569(0.497-0.651) | 0.588(0.521-0.663) | 0.611(0.551-0.677) | 0.639(0.586-0.698) | 0.676(0.626-0.73) | 0.724(0.676-0.775) | 0.788(0.748-0.83) | 0.883(0.863-0.904) | 1.056(1.048-1.064) | 1.398(1.357-1.44) | 2.085(2.003-2.171) | 2.996(2.84-3.162) | 3.219(3.03-3.419) | 2.303(2.163-2.452) |
| lag21 | 0.518(0.434-0.618) | 0.529(0.449-0.623) | 0.543(0.466-0.632) | 0.56(0.487-0.644) | 0.581(0.513-0.658) | 0.606(0.544-0.675) | 0.637(0.581-0.698) | 0.676(0.623-0.733) | 0.726(0.675-0.78) | 0.79(0.748-0.834) | 0.884(0.862-0.906) | 1.056(1.048-1.065) | 1.404(1.361-1.448) | 2.119(2.031-2.21) | 3.084(2.915-3.262) | 3.324(3.12-3.542) | 2.353(2.203-2.514) |

* Indicates a lag day that continues to turn into a high risk

|  | Table S10 Risk of DV with each 1 °C change in lag time from 14 °C to 30 °C | | | | | | | | | | | | | | | | |
| --- | --- | --- | --- | --- | --- | --- | --- | --- | --- | --- | --- | --- | --- | --- | --- | --- | --- |
| Lag(days) | Temperature(℃), RR(95%CI) | | | | | | | | | | | | | | | | |
|  | 14 | 15 | 16 | 17 | 18 | 19 | 20 | 21 | 22 | 23 | 24 | 25 | 26 | 27 | 28 | 29 | 30 |
| lag0 | 0.923(0.855-0.996) | 0.947(0.88-1.019) | 0.968(0.903-1.038) | 0.985(0.923-1.051) | 0.997(0.939-1.059) | 1.004(0.952-1.06) | 1.006(0.96-1.054) | 1.003(0.965-1.042) | 0.998(0.968-1.029) | 0.995(0.973-1.017) | 0.995(0.985-1.006) | 1.003(0.998-1.007) | 1.017(0.992-1.043) | 1.034(0.989-1.082) | 1.049(0.987-1.114) | 1.055(0.983-1.132) | 1.053(0.968-1.146) |
| lag1 | 0.801(0.749-0.857) | 0.83(0.778-0.886) | 0.857(0.805-0.913) | 0.882(0.831-0.936) | 0.904(0.856-0.956) | 0.924(0.879-0.971) | 0.939(0.9-0.981) | 0.952(0.918-0.987) | 0.963(0.936-0.992) | 0.975(0.954-0.996) | 0.988(0.977-0.999) | 1.005(1-1.01) | 1.026(1-1.052) | 1.047(1-1.096) | 1.066(1.002-1.133) | 1.077(1.004-1.155) | 1.081(0.997-1.173) |
| lag2 | 0.824(0.774-0.877) | 0.857(0.807-0.911) | 0.888(0.838-0.942) | 0.916(0.867-0.968) | 0.94(0.893-0.99) | 0.959(0.916-1.004) | 0.973(0.934-1.012) | 0.981(0.949-1.015) | 0.987(0.96-1.014) | 0.991(0.971-1.011) | 0.995(0.985-1.005) | 1.002(0.998-1.007) | 1.012(0.988-1.037) | 1.025(0.981-1.071) | 1.038(0.98-1.1) | 1.051(0.985-1.122) | 1.064(0.986-1.148) |
| lag3 | 0.862(0.806-0.922) | 0.896(0.839-0.957) | 0.927(0.87-0.987) | 0.953(0.898-1.012) | 0.974(0.921-1.03) | 0.989(0.94-1.04) | 0.997(0.955-1.041) | 0.999(0.963-1.036) | 0.998(0.969-1.028) | 0.996(0.975-1.018) | 0.997(0.986-1.008) | 1.002(0.997-1.007) | 1.013(0.987-1.04) | 1.027(0.979-1.076) | 1.04(0.978-1.106) | 1.05(0.98-1.125) | 1.056(0.976-1.143) |
| lag4 | 0.904(0.844-0.969) | 0.936(0.874-1.001) | 0.963(0.902-1.028) | 0.985(0.926-1.048) | 1.001(0.945-1.061) | 1.011(0.959-1.065) | 1.013(0.968-1.06) | 1.009(0.971-1.048) | 1.002(0.972-1.034) | 0.997(0.974-1.02) | 0.996(0.984-1.008) | 1.003(0.998-1.008) | 1.018(0.99-1.047) | 1.036(0.985-1.089) | 1.05(0.984-1.121) | 1.055(0.982-1.134) | 1.051(0.969-1.14) |
| lag5 | 0.952(0.883-1.027) | 0.979(0.91-1.054) | 1.002(0.933-1.076) | 1.019(0.952-1.09) | 1.029(0.966-1.096) | 1.033(0.976-1.094) | 1.029(0.98-1.082) | 1.02(0.978-1.063) | 1.009(0.975-1.044) | 1(0.975-1.025) | 0.996(0.984-1.009) | 1.003(0.997-1.008) | 1.019(0.988-1.05) | 1.038(0.983-1.096) | 1.052(0.98-1.129) | 1.053(0.975-1.138) | 1.043(0.955-1.138) |
| lag6 | 1.007(0.928-1.091) | 1.028(0.95-1.113) | 1.045(0.969-1.128) | 1.056(0.983-1.136) | 1.061(0.992-1.135) | 1.059(0.997-1.126) | 1.05(0.996-1.107) | 1.035(0.991-1.082) | 1.019(0.984-1.056) | 1.006(0.98-1.033) | 0.999(0.985-1.013) | 1.002(0.996-1.008) | 1.015(0.983-1.048) | 1.032(0.974-1.092) | 1.043(0.969-1.124) | 1.043(0.962-1.131) | 1.031(0.942-1.13) |
| lag7 | 1.066(0.979-1.162) | 1.083(0.996-1.177) | 1.094(1.009-1.186)* | 1.099(1.018-1.187)* | 1.098(1.023-1.179)* | 1.09(1.023-1.162)* | 1.075(1.017-1.136)* | 1.054(1.007-1.104)* | 1.033(0.995-1.072) | 1.014(0.987-1.042) | 1.002(0.988-1.017) | 1.001(0.995-1.007) | 1.01(0.977-1.044) | 1.022(0.963-1.085) | 1.032(0.956-1.114) | 1.031(0.949-1.121) | 1.021(0.929-1.122) |
| lag8 | 1.131(1.033-1.239)* | 1.143(1.046-1.249)* | 1.149(1.054-1.252) | 1.148(1.058-1.246) | 1.141(1.058-1.23) | 1.126(1.052-1.205) | 1.103(1.041-1.17) | 1.076(1.025-1.13) | 1.048(1.008-1.09)* | 1.023(0.994-1.053) | 1.006(0.991-1.021) | 1(0.993-1.006) | 1.004(0.97-1.04) | 1.014(0.953-1.08) | 1.022(0.943-1.108) | 1.022(0.936-1.115) | 1.013(0.917-1.118) |
| lag9 | 1.201(1.09-1.324) | 1.209(1.099-1.329) | 1.209(1.104-1.325) | 1.203(1.103-1.312) | 1.189(1.097-1.288) | 1.166(1.085-1.253) | 1.136(1.067-1.209) | 1.1(1.044-1.158) | 1.064(1.02-1.109) | 1.032(1.001-1.064)* | 1.009(0.993-1.025) | 0.999(0.992-1.006) | 1.001(0.965-1.039) | 1.009(0.945-1.078) | 1.017(0.934-1.107) | 1.017(0.928-1.115) | 1.009(0.909-1.121) |
| lag10 | 1.276(1.152-1.414) | 1.28(1.158-1.415) | 1.276(1.159-1.406) | 1.264(1.153-1.385) | 1.242(1.141-1.352) | 1.211(1.122-1.307) | 1.171(1.096-1.25) | 1.125(1.065-1.188) | 1.08(1.033-1.128) | 1.04(1.007-1.074) | 1.012(0.995-1.029) | 0.998(0.991-1.005) | 1(0.962-1.039) | 1.009(0.942-1.082) | 1.018(0.932-1.113) | 1.019(0.926-1.121) | 1.011(0.906-1.128) |
| lag11 | 1.356(1.218-1.51) | 1.358(1.223-1.508) | 1.35(1.22-1.493) | 1.331(1.209-1.464) | 1.301(1.191-1.421) | 1.26(1.164-1.364) | 1.208(1.128-1.294) | 1.151(1.087-1.218) | 1.095(1.046-1.146) | 1.047(1.013-1.083) | 1.013(0.996-1.031) | 0.998(0.991-1.006) | 1.001(0.962-1.043) | 1.014(0.944-1.089) | 1.026(0.935-1.124) | 1.026(0.93-1.134) | 1.016(0.907-1.138) |
| lag12 | 1.442(1.289-1.613) | 1.441(1.292-1.607) | 1.429(1.286-1.587) | 1.403(1.27-1.55) | 1.364(1.244-1.496) | 1.312(1.208-1.425) | 1.248(1.162-1.34) | 1.177(1.11-1.249) | 1.11(1.059-1.164) | 1.053(1.018-1.091) | 1.014(0.996-1.033) | 0.998(0.991-1.006) | 1.005(0.964-1.048) | 1.023(0.95-1.102) | 1.038(0.944-1.142) | 1.039(0.938-1.151) | 1.025(0.912-1.153) |
| lag13 | 1.534(1.365-1.723) | 1.531(1.366-1.714) | 1.513(1.356-1.688) | 1.48(1.334-1.641) | 1.43(1.3-1.574) | 1.366(1.254-1.489) | 1.288(1.196-1.387) | 1.203(1.131-1.28) | 1.124(1.07-1.18) | 1.058(1.021-1.097) | 1.015(0.996-1.034) | 0.999(0.991-1.007) | 1.011(0.968-1.055) | 1.035(0.959-1.118) | 1.056(0.957-1.165) | 1.057(0.95-1.175) | 1.037(0.919-1.171) |
| lag14 | 1.631(1.445-1.842) | 1.626(1.445-1.829) | 1.602(1.43-1.795) | 1.56(1.401-1.738) | 1.5(1.357-1.657) | 1.422(1.3-1.555) | 1.328(1.229-1.435) | 1.228(1.152-1.31) | 1.136(1.079-1.196) | 1.062(1.023-1.103) | 1.014(0.995-1.034) | 1(0.992-1.008) | 1.018(0.973-1.065) | 1.051(0.97-1.138) | 1.077(0.972-1.193) | 1.077(0.965-1.202) | 1.051(0.927-1.191) |
| lag15 | 1.736(1.53-1.97) | 1.726(1.527-1.952) | 1.696(1.506-1.909) | 1.644(1.469-1.839) | 1.57(1.415-1.743) | 1.477(1.346-1.622) | 1.367(1.262-1.482) | 1.252(1.171-1.338) | 1.147(1.087-1.21) | 1.065(1.024-1.107) | 1.013(0.993-1.034) | 1.001(0.992-1.01) | 1.027(0.98-1.076) | 1.069(0.983-1.161) | 1.102(0.99-1.225) | 1.101(0.982-1.234) | 1.066(0.935-1.214) |
| lag16 | 1.849(1.621-2.108) | 1.833(1.613-2.083) | 1.793(1.585-2.029) | 1.729(1.538-1.944) | 1.642(1.473-1.829) | 1.532(1.39-1.689) | 1.405(1.292-1.528) | 1.274(1.188-1.366) | 1.156(1.094-1.222) | 1.066(1.024-1.11) | 1.012(0.991-1.033) | 1.002(0.993-1.011) | 1.036(0.987-1.088) | 1.089(0.999-1.187) | 1.129(1.011-1.261)* | 1.126(1-1.268)* | 1.081(0.943-1.238) |
| lag17 | 1.969(1.718-2.256) | 1.945(1.703-2.221) | 1.894(1.667-2.153) | 1.816(1.608-2.05) | 1.712(1.53-1.916) | 1.586(1.433-1.754) | 1.441(1.32-1.572) | 1.293(1.203-1.39) | 1.164(1.098-1.233) | 1.066(1.022-1.112) | 1.01(0.988-1.032) | 1.004(0.995-1.013) | 1.047(0.996-1.101) | 1.111(1.016-1.214)* | 1.158(1.034-1.298) | 1.153(1.02-1.304) | 1.096(0.952-1.262) |
| lag18 | 2.098(1.822-2.416) | 2.063(1.798-2.366) | 1.997(1.75-2.28) | 1.903(1.678-2.158) | 1.781(1.585-2.001) | 1.636(1.474-1.817) | 1.473(1.346-1.612) | 1.31(1.215-1.411) | 1.169(1.101-1.241) | 1.065(1.02-1.112) | 1.007(0.985-1.03) | 1.006(0.996-1.015) | 1.059(1.005-1.115)* | 1.134(1.035-1.243) | 1.19(1.058-1.338) | 1.181(1.041-1.341) | 1.111(0.96-1.285) |
| lag19 | 2.236(1.933-2.587) | 2.185(1.896-2.519) | 2.103(1.834-2.412) | 1.989(1.747-2.266) | 1.848(1.638-2.085) | 1.683(1.511-1.876) | 1.502(1.368-1.649) | 1.323(1.225-1.43) | 1.172(1.102-1.246) | 1.062(1.016-1.111) | 1.004(0.982-1.027) | 1.008(0.998-1.017) | 1.071(1.016-1.129) | 1.159(1.056-1.273) | 1.223(1.085-1.379) | 1.21(1.063-1.378) | 1.125(0.968-1.308) |
| lag20 | 2.385(2.05-2.774) | 2.314(1.997-2.681) | 2.21(1.917-2.547) | 2.074(1.812-2.374) | 1.911(1.686-2.166) | 1.726(1.543-1.932) | 1.527(1.385-1.682) | 1.334(1.231-1.445) | 1.173(1.1-1.25) | 1.059(1.011-1.109) | 1.001(0.978-1.024) | 1.01(0.999-1.02) | 1.084(1.027-1.145) | 1.186(1.077-1.305) | 1.257(1.112-1.422) | 1.24(1.085-1.417) | 1.138(0.974-1.33) |
| lag21 | 2.544(2.172-2.98) | 2.447(2.098-2.855) | 2.317(1.997-2.689) | 2.157(1.873-2.484) | 1.97(1.728-2.247) | 1.764(1.568-1.985) | 1.547(1.397-1.713) | 1.341(1.232-1.458) | 1.171(1.096-1.252) | 1.054(1.005-1.106) | 0.997(0.973-1.021) | 1.012(1.001-1.022)* | 1.098(1.038-1.162) | 1.213(1.098-1.341) | 1.293(1.138-1.47) | 1.27(1.104-1.46) | 1.15(0.976-1.355) |

* Indicates a lag day that continues to turn into a high risk

|  | Table S11 Risk of DN with each 1 °C change in lag time from 14 °C to 30 °C | | | | | | | | | | | | | | | | |
| --- | --- | --- | --- | --- | --- | --- | --- | --- | --- | --- | --- | --- | --- | --- | --- | --- | --- |
| Lag(days) | Temperature(℃), RR(95%CI) | | | | | | | | | | | | | | | | |
|  | 14 | 15 | 16 | 17 | 18 | 19 | 20 | 21 | 22 | 23 | 24 | 25 | 26 | 27 | 28 | 29 | 30 |
| lag0 | 0.885(0.84-0.932) | 0.902(0.859-0.948) | 0.919(0.877-0.964) | 0.936(0.895-0.978) | 0.951(0.913-0.991) | 0.966(0.931-1.001) | 0.979(0.95-1.01) | 0.991(0.967-1.016) | 1(0.982-1.019) | 1.005(0.992-1.018) | 1.004(0.998-1.011) | 0.998(0.995-1) | 0.985(0.972-0.999) | 0.973(0.949-0.998) | 0.966(0.933-0.999) | 0.967(0.93-1.006) | 0.978(0.935-1.022) |
| lag1 | 0.857(0.813-0.905) | 0.878(0.835-0.925) | 0.899(0.856-0.944) | 0.918(0.877-0.961) | 0.937(0.898-0.977) | 0.954(0.919-0.99) | 0.969(0.939-1.001) | 0.983(0.958-1.009) | 0.994(0.975-1.013) | 1.001(0.988-1.014) | 1.002(0.996-1.009) | 0.998(0.996-1.001) | 0.989(0.975-1.004) | 0.979(0.953-1.005) | 0.972(0.937-1.007) | 0.971(0.932-1.011) | 0.977(0.933-1.023) |
| lag2 | 0.857(0.813-0.904) | 0.877(0.834-0.923) | 0.897(0.854-0.941) | 0.915(0.874-0.958) | 0.932(0.893-0.972) | 0.948(0.912-0.984) | 0.962(0.931-0.993) | 0.974(0.95-1) | 0.985(0.966-1.004) | 0.993(0.98-1.006) | 0.998(0.992-1.005) | 1(0.997-1.003) | 1(0.984-1.015) | 0.997(0.97-1.025) | 0.994(0.958-1.031) | 0.991(0.951-1.032) | 0.988(0.944-1.033) |
| lag3 | 0.855(0.808-0.905) | 0.874(0.827-0.923) | 0.892(0.846-0.94) | 0.909(0.864-0.955) | 0.924(0.883-0.968) | 0.939(0.901-0.978) | 0.952(0.92-0.986) | 0.964(0.938-0.991) | 0.975(0.955-0.995) | 0.984(0.971-0.999) | 0.994(0.987-1.001) | 1.002(0.999-1.005) | 1.01(0.994-1.027) | 1.016(0.986-1.047) | 1.018(0.978-1.059) | 1.014(0.971-1.059) | 1.005(0.96-1.054) |
| lag4 | 0.85(0.8-0.903) | 0.867(0.818-0.92) | 0.884(0.836-0.935) | 0.9(0.853-0.949) | 0.915(0.871-0.961) | 0.929(0.889-0.97) | 0.941(0.907-0.977) | 0.953(0.926-0.982) | 0.965(0.944-0.986) | 0.976(0.962-0.991) | 0.989(0.982-0.997) | 1.004(1.001-1.007)* | 1.02(1.002-1.038)* | 1.033(1.002-1.066)* | 1.041(0.999-1.084) | 1.038(0.993-1.085) | 1.026(0.978-1.076) |
| lag5 | 0.844(0.791-0.901) | 0.86(0.808-0.916) | 0.876(0.824-0.93) | 0.89(0.841-0.943) | 0.905(0.858-0.954) | 0.918(0.876-0.962) | 0.931(0.895-0.968) | 0.943(0.914-0.973) | 0.955(0.933-0.978) | 0.969(0.954-0.984) | 0.985(0.978-0.993) | 1.006(1.002-1.009) | 1.029(1.01-1.048) | 1.05(1.016-1.085) | 1.062(1.018-1.109)* | 1.061(1.013-1.111)* | 1.048(0.997-1.101) |
| lag6 | 0.838(0.781-0.9) | 0.853(0.797-0.913) | 0.868(0.813-0.926) | 0.882(0.829-0.938) | 0.895(0.846-0.948) | 0.908(0.863-0.956) | 0.921(0.882-0.961) | 0.933(0.902-0.965) | 0.946(0.923-0.97) | 0.962(0.946-0.979) | 0.982(0.974-0.99) | 1.007(1.004-1.011) | 1.037(1.017-1.057) | 1.064(1.027-1.102) | 1.082(1.033-1.132) | 1.083(1.031-1.137) | 1.068(1.013-1.126)* |
| lag7 | 0.834(0.773-0.9) | 0.847(0.787-0.912) | 0.861(0.802-0.924) | 0.874(0.817-0.935) | 0.887(0.834-0.943) | 0.9(0.852-0.95) | 0.912(0.871-0.955) | 0.925(0.891-0.959) | 0.939(0.914-0.965) | 0.956(0.939-0.974) | 0.979(0.97-0.988) | 1.008(1.005-1.012) | 1.043(1.022-1.065) | 1.077(1.037-1.118) | 1.099(1.047-1.154) | 1.102(1.046-1.161) | 1.087(1.028-1.149) |
| lag8 | 0.831(0.766-0.902) | 0.843(0.779-0.912) | 0.855(0.793-0.923) | 0.868(0.808-0.932) | 0.88(0.824-0.94) | 0.892(0.841-0.946) | 0.904(0.861-0.95) | 0.917(0.882-0.954) | 0.932(0.906-0.96) | 0.952(0.933-0.97) | 0.977(0.967-0.986) | 1.009(1.005-1.013) | 1.049(1.027-1.072) | 1.087(1.045-1.131) | 1.114(1.058-1.172) | 1.118(1.059-1.181) | 1.103(1.04-1.169) |
| lag9 | 0.829(0.76-0.904) | 0.84(0.773-0.913) | 0.851(0.786-0.922) | 0.863(0.8-0.931) | 0.874(0.815-0.938) | 0.886(0.833-0.943) | 0.898(0.853-0.946) | 0.911(0.875-0.95) | 0.927(0.9-0.956) | 0.947(0.929-0.967) | 0.974(0.965-0.984) | 1.01(1.006-1.015) | 1.054(1.03-1.078) | 1.096(1.053-1.142) | 1.126(1.068-1.187) | 1.132(1.071-1.198) | 1.117(1.052-1.185) |
| lag10 | 0.828(0.756-0.907) | 0.838(0.768-0.915) | 0.848(0.78-0.923) | 0.859(0.793-0.93) | 0.87(0.808-0.936) | 0.881(0.826-0.941) | 0.893(0.846-0.943) | 0.906(0.868-0.946) | 0.923(0.894-0.952) | 0.944(0.924-0.964) | 0.973(0.963-0.983) | 1.011(1.007-1.015) | 1.058(1.033-1.083) | 1.104(1.058-1.151) | 1.136(1.076-1.2) | 1.144(1.08-1.212) | 1.129(1.061-1.2) |
| lag11 | 0.829(0.753-0.912) | 0.837(0.764-0.918) | 0.847(0.775-0.925) | 0.856(0.788-0.931) | 0.866(0.802-0.936) | 0.877(0.819-0.94) | 0.889(0.839-0.942) | 0.902(0.863-0.944) | 0.919(0.889-0.95) | 0.941(0.921-0.962) | 0.971(0.961-0.982) | 1.012(1.007-1.016) | 1.061(1.036-1.087) | 1.11(1.062-1.159) | 1.145(1.082-1.211) | 1.154(1.088-1.224) | 1.139(1.069-1.213) |
| lag12 | 0.83(0.751-0.917) | 0.837(0.76-0.922) | 0.846(0.771-0.928) | 0.854(0.783-0.933) | 0.864(0.797-0.937) | 0.874(0.814-0.94) | 0.886(0.834-0.941) | 0.899(0.858-0.943) | 0.917(0.885-0.949) | 0.939(0.918-0.961) | 0.97(0.96-0.981) | 1.012(1.007-1.017) | 1.063(1.037-1.09) | 1.114(1.065-1.165) | 1.151(1.086-1.22) | 1.162(1.093-1.235) | 1.148(1.075-1.225) |
| lag13 | 0.832(0.749-0.924) | 0.838(0.758-0.928) | 0.845(0.767-0.932) | 0.853(0.778-0.936) | 0.862(0.792-0.939) | 0.872(0.809-0.941) | 0.884(0.83-0.941) | 0.897(0.854-0.943) | 0.915(0.882-0.948) | 0.938(0.916-0.961) | 0.97(0.959-0.981) | 1.012(1.007-1.017) | 1.065(1.037-1.093) | 1.117(1.066-1.17) | 1.155(1.088-1.227) | 1.168(1.096-1.244) | 1.155(1.08-1.236) |
| lag14 | 0.835(0.748-0.932) | 0.84(0.756-0.934) | 0.846(0.764-0.937) | 0.853(0.775-0.94) | 0.862(0.788-0.942) | 0.871(0.805-0.943) | 0.882(0.826-0.943) | 0.896(0.85-0.944) | 0.913(0.879-0.949) | 0.937(0.914-0.961) | 0.969(0.958-0.981) | 1.012(1.007-1.018) | 1.066(1.037-1.095) | 1.119(1.066-1.174) | 1.158(1.089-1.233) | 1.173(1.098-1.252) | 1.161(1.083-1.246) |
| lag15 | 0.839(0.748-0.94) | 0.843(0.754-0.941) | 0.848(0.762-0.943) | 0.854(0.772-0.944) | 0.862(0.785-0.945) | 0.871(0.802-0.946) | 0.882(0.823-0.945) | 0.895(0.848-0.945) | 0.913(0.877-0.95) | 0.937(0.913-0.961) | 0.969(0.957-0.981) | 1.012(1.007-1.018) | 1.066(1.036-1.096) | 1.119(1.064-1.177) | 1.16(1.088-1.237) | 1.176(1.099-1.258) | 1.167(1.085-1.255) |
| lag16 | 0.843(0.749-0.95) | 0.846(0.754-0.949) | 0.85(0.761-0.949) | 0.855(0.77-0.95) | 0.862(0.783-0.95) | 0.871(0.799-0.949) | 0.882(0.82-0.948) | 0.895(0.846-0.947) | 0.913(0.876-0.951) | 0.937(0.912-0.963) | 0.969(0.957-0.982) | 1.012(1.007-1.018) | 1.065(1.035-1.097) | 1.119(1.062-1.178) | 1.16(1.086-1.239) | 1.178(1.098-1.263) | 1.172(1.087-1.263) |
| lag17 | 0.848(0.75-0.959) | 0.85(0.754-0.957) | 0.853(0.76-0.956) | 0.857(0.769-0.956) | 0.864(0.781-0.955) | 0.872(0.797-0.953) | 0.882(0.819-0.951) | 0.896(0.844-0.95) | 0.913(0.875-0.953) | 0.937(0.912-0.964) | 0.97(0.957-0.982) | 1.012(1.007-1.018) | 1.064(1.033-1.096) | 1.117(1.059-1.178) | 1.159(1.083-1.24) | 1.178(1.097-1.266) | 1.176(1.088-1.27) |
| lag18 | 0.854(0.752-0.97) | 0.854(0.755-0.966) | 0.856(0.76-0.964) | 0.86(0.768-0.962) | 0.865(0.78-0.96) | 0.873(0.796-0.958) | 0.884(0.817-0.955) | 0.897(0.844-0.953) | 0.915(0.875-0.956) | 0.938(0.912-0.966) | 0.97(0.957-0.983) | 1.012(1.006-1.018) | 1.063(1.031-1.095) | 1.114(1.056-1.176) | 1.156(1.079-1.239) | 1.178(1.095-1.267) | 1.179(1.089-1.277) |
| lag19 | 0.86(0.754-0.981) | 0.859(0.756-0.976) | 0.86(0.761-0.973) | 0.863(0.768-0.97) | 0.868(0.779-0.967) | 0.875(0.796-0.963) | 0.885(0.817-0.96) | 0.899(0.844-0.957) | 0.916(0.875-0.959) | 0.94(0.912-0.968) | 0.971(0.958-0.985) | 1.012(1.006-1.017) | 1.061(1.028-1.094) | 1.111(1.051-1.174) | 1.152(1.073-1.237) | 1.176(1.091-1.268) | 1.182(1.089-1.283) |
| lag20 | 0.867(0.756-0.994) | 0.865(0.757-0.987) | 0.865(0.761-0.982) | 0.867(0.768-0.978) | 0.871(0.779-0.974) | 0.878(0.795-0.97) | 0.888(0.817-0.966) | 0.901(0.844-0.962) | 0.919(0.876-0.964) | 0.942(0.913-0.972) | 0.972(0.958-0.986) | 1.011(1.005-1.017) | 1.058(1.024-1.092) | 1.106(1.045-1.171) | 1.148(1.067-1.235) | 1.174(1.086-1.269) | 1.185(1.088-1.29) |
| lag21 | 0.874(0.758-1.008) | 0.871(0.758-1) | 0.87(0.761-0.994) | 0.871(0.768-0.989) | 0.875(0.779-0.984) | 0.882(0.794-0.978) | 0.891(0.816-0.973) | 0.904(0.844-0.969) | 0.921(0.876-0.969) | 0.944(0.914-0.975) | 0.973(0.959-0.988) | 1.01(1.004-1.017) | 1.055(1.02-1.091) | 1.101(1.037-1.169) | 1.142(1.058-1.233) | 1.171(1.079-1.27) | 1.187(1.085-1.299) |

* Indicates a lag day that continues to turn into a high risk

| Table S12 Risk of male DV with each 1 °C change in lag time from 13.8 °C to 30 °C | | | | | | | | | | | | | | | | | | |
| --- | --- | --- | --- | --- | --- | --- | --- | --- | --- | --- | --- | --- | --- | --- | --- | --- | --- | --- |
| Lag(days) | Temperature(℃), RR(95%CI) | | | | | | | | | | | | | | | | | |
|  | 13.8 | 14 | 15 | 16 | 17 | 18 | 19 | 20 | 21 | 22 | 23 | 24 | 25 | 26 | 27 | 28 | 29 | 30 |
| lag0 | 0.924(0.849-1.007) | 0.93(0.854-1.012) | 0.953(0.879-1.034) | 0.973(0.901-1.051) | 0.989(0.92-1.064) | 1.001(0.936-1.069) | 1.007(0.948-1.068) | 1.007(0.957-1.06) | 1.003(0.961-1.046) | 0.997(0.964-1.031) | 0.993(0.97-1.017) | 0.994(0.983-1.006) | 1.003(0.998-1.008) | 1.02(0.993-1.048) | 1.04(0.99-1.093) | 1.056(0.988-1.129) | 1.062(0.983-1.148) | 1.059(0.966-1.162) |
| lag1 | 0.797(0.739-0.859) | 0.802(0.744-0.864) | 0.827(0.769-0.889) | 0.851(0.794-0.912) | 0.874(0.818-0.933) | 0.895(0.842-0.951) | 0.914(0.865-0.966) | 0.931(0.888-0.977) | 0.947(0.909-0.985) | 0.961(0.93-0.992) | 0.974(0.952-0.997) | 0.989(0.977-1) | 1.004(0.999-1.009) | 1.022(0.994-1.051) | 1.04(0.988-1.093) | 1.056(0.988-1.13) | 1.071(0.992-1.156) | 1.083(0.99-1.184) |
| lag2 | 0.815(0.76-0.873) | 0.821(0.766-0.88) | 0.852(0.796-0.911) | 0.88(0.825-0.939) | 0.906(0.852-0.964) | 0.929(0.877-0.984) | 0.948(0.901-0.998) | 0.963(0.921-1.007) | 0.974(0.938-1.011) | 0.982(0.953-1.012) | 0.988(0.967-1.01) | 0.995(0.984-1.006) | 1.002(0.997-1.007) | 1.012(0.985-1.039) | 1.024(0.976-1.074) | 1.039(0.975-1.107) | 1.057(0.984-1.136) | 1.078(0.992-1.172) |
| lag3 | 0.851(0.79-0.918) | 0.858(0.796-0.924) | 0.889(0.827-0.956) | 0.917(0.855-0.984) | 0.942(0.881-1.006) | 0.962(0.904-1.023) | 0.976(0.924-1.032) | 0.986(0.939-1.034) | 0.99(0.951-1.03) | 0.991(0.959-1.024) | 0.992(0.969-1.016) | 0.995(0.983-1.007) | 1.002(0.997-1.008) | 1.015(0.986-1.045) | 1.031(0.979-1.086) | 1.048(0.979-1.122) | 1.063(0.986-1.147) | 1.076(0.987-1.174) |
| lag4 | 0.894(0.827-0.966) | 0.9(0.833-0.972) | 0.928(0.861-1) | 0.952(0.885-1.024) | 0.972(0.907-1.042) | 0.987(0.926-1.053) | 0.996(0.94-1.056) | 0.999(0.95-1.051) | 0.997(0.956-1.04) | 0.994(0.96-1.028) | 0.991(0.966-1.017) | 0.993(0.981-1.007) | 1.004(0.998-1.009) | 1.022(0.991-1.054) | 1.044(0.987-1.103) | 1.062(0.988-1.141) | 1.072(0.991-1.16) | 1.073(0.982-1.174) |
| lag5 | 0.943(0.867-1.026) | 0.948(0.872-1.031) | 0.971(0.895-1.054) | 0.99(0.915-1.072) | 1.004(0.931-1.083) | 1.014(0.945-1.087) | 1.017(0.955-1.083) | 1.014(0.96-1.072) | 1.007(0.962-1.055) | 0.999(0.962-1.037) | 0.993(0.966-1.021) | 0.994(0.98-1.008) | 1.004(0.998-1.01) | 1.024(0.99-1.059) | 1.047(0.986-1.112) | 1.066(0.986-1.153) | 1.073(0.985-1.168) | 1.067(0.969-1.175) |
| lag6 | 0.998(0.912-1.092) | 1.002(0.916-1.096) | 1.019(0.934-1.113) | 1.033(0.949-1.124) | 1.041(0.961-1.128) | 1.044(0.969-1.125) | 1.042(0.974-1.115) | 1.034(0.975-1.096) | 1.021(0.973-1.072) | 1.009(0.97-1.049) | 0.999(0.97-1.028) | 0.996(0.981-1.011) | 1.003(0.997-1.01) | 1.021(0.985-1.057) | 1.042(0.978-1.11) | 1.059(0.976-1.149) | 1.065(0.974-1.163) | 1.058(0.958-1.17) |
| lag7 | 1.059(0.963-1.166) | 1.062(0.966-1.168) | 1.074(0.978-1.178) | 1.081(0.988-1.182) | 1.083(0.995-1.179) | 1.08(0.998-1.169) | 1.072(0.998-1.151) | 1.058(0.995-1.125) | 1.04(0.988-1.094) | 1.021(0.98-1.064) | 1.007(0.977-1.038) | 0.999(0.984-1.015) | 1.002(0.995-1.009) | 1.015(0.979-1.053) | 1.033(0.968-1.103) | 1.049(0.964-1.141) | 1.055(0.962-1.156) | 1.051(0.947-1.166) |
| lag8 | 1.126(1.017-1.247) | 1.128(1.019-1.248) | 1.134(1.027-1.251) | 1.135(1.032-1.248) | 1.131(1.033-1.238) | 1.122(1.031-1.22) | 1.107(1.026-1.193) | 1.086(1.017-1.158) | 1.061(1.005-1.12) | 1.036(0.992-1.082) | 1.016(0.984-1.049) | 1.003(0.986-1.019) | 1.001(0.994-1.008) | 1.01(0.972-1.05) | 1.025(0.957-1.098) | 1.039(0.951-1.135) | 1.047(0.951-1.152) | 1.047(0.939-1.168) |
| lag9 | 1.197(1.074-1.335) | 1.198(1.075-1.335) | 1.199(1.08-1.333) | 1.195(1.08-1.323) | 1.185(1.076-1.305) | 1.169(1.069-1.278) | 1.146(1.058-1.242) | 1.117(1.043-1.197) | 1.084(1.024-1.148) | 1.052(1.005-1.102) | 1.025(0.991-1.06) | 1.006(0.989-1.024) | 1(0.992-1.007) | 1.006(0.966-1.048) | 1.02(0.948-1.097) | 1.034(0.942-1.135) | 1.044(0.943-1.155) | 1.047(0.933-1.175) |
| lag10 | 1.274(1.136-1.429) | 1.274(1.137-1.428) | 1.271(1.138-1.421) | 1.262(1.134-1.405) | 1.245(1.125-1.379) | 1.222(1.112-1.342) | 1.19(1.094-1.295) | 1.152(1.071-1.239) | 1.109(1.044-1.178) | 1.068(1.018-1.121) | 1.033(0.997-1.07) | 1.009(0.991-1.027) | 0.999(0.991-1.007) | 1.005(0.963-1.049) | 1.019(0.944-1.1) | 1.035(0.939-1.142) | 1.047(0.942-1.163) | 1.053(0.933-1.187) |
| lag11 | 1.356(1.202-1.529) | 1.356(1.203-1.528) | 1.349(1.201-1.516) | 1.335(1.193-1.494) | 1.312(1.18-1.459) | 1.28(1.16-1.412) | 1.239(1.134-1.353) | 1.189(1.102-1.283) | 1.135(1.066-1.209) | 1.084(1.031-1.14) | 1.041(1.003-1.08) | 1.011(0.992-1.03) | 0.999(0.991-1.007) | 1.006(0.962-1.051) | 1.023(0.945-1.107) | 1.043(0.942-1.154) | 1.056(0.947-1.178) | 1.062(0.938-1.203) |
| lag12 | 1.443(1.274-1.636) | 1.443(1.274-1.634) | 1.434(1.27-1.618) | 1.414(1.258-1.589) | 1.384(1.239-1.546) | 1.342(1.212-1.487) | 1.291(1.177-1.415) | 1.229(1.135-1.33) | 1.162(1.088-1.241) | 1.099(1.043-1.158) | 1.047(1.008-1.088) | 1.012(0.992-1.032) | 0.999(0.991-1.008) | 1.009(0.963-1.056) | 1.031(0.95-1.119) | 1.055(0.95-1.172) | 1.071(0.956-1.198) | 1.075(0.946-1.223) |
| lag13 | 1.537(1.349-1.751) | 1.536(1.349-1.749) | 1.524(1.343-1.729) | 1.499(1.327-1.692) | 1.46(1.301-1.638) | 1.409(1.266-1.567) | 1.344(1.222-1.479) | 1.269(1.168-1.377) | 1.188(1.11-1.272) | 1.113(1.054-1.175) | 1.052(1.011-1.095) | 1.012(0.992-1.033) | 1(0.991-1.008) | 1.014(0.967-1.063) | 1.043(0.958-1.135) | 1.073(0.962-1.196) | 1.089(0.969-1.224) | 1.091(0.955-1.247) |
| lag14 | 1.637(1.429-1.875) | 1.636(1.429-1.872) | 1.62(1.42-1.847) | 1.588(1.399-1.802) | 1.541(1.366-1.737) | 1.478(1.322-1.651) | 1.4(1.267-1.546) | 1.309(1.201-1.426) | 1.213(1.13-1.303) | 1.126(1.064-1.192) | 1.057(1.014-1.101) | 1.012(0.991-1.034) | 1(0.991-1.01) | 1.021(0.971-1.072) | 1.058(0.968-1.155) | 1.094(0.977-1.224) | 1.112(0.985-1.255) | 1.109(0.966-1.273) |
| lag15 | 1.745(1.515-2.009) | 1.742(1.514-2.006) | 1.721(1.501-1.974) | 1.682(1.474-1.919) | 1.624(1.433-1.841) | 1.548(1.379-1.738) | 1.455(1.312-1.615) | 1.349(1.233-1.475) | 1.238(1.149-1.333) | 1.138(1.072-1.207) | 1.06(1.015-1.106) | 1.012(0.99-1.034) | 1.001(0.992-1.011) | 1.029(0.977-1.083) | 1.075(0.981-1.178) | 1.118(0.995-1.258) | 1.137(1.002-1.289) | 1.127(0.976-1.302) |
| lag16 | 1.86(1.605-2.154) | 1.857(1.604-2.149) | 1.829(1.586-2.109) | 1.78(1.552-2.042) | 1.71(1.501-1.948) | 1.619(1.436-1.827) | 1.511(1.356-1.683) | 1.387(1.263-1.522) | 1.26(1.166-1.361) | 1.147(1.079-1.22) | 1.061(1.015-1.11) | 1.01(0.987-1.034) | 1.003(0.993-1.013) | 1.038(0.984-1.095) | 1.094(0.995-1.204) | 1.146(1.014-1.294) | 1.164(1.021-1.326) | 1.146(0.987-1.331) |
| lag17 | 1.983(1.703-2.31) | 1.978(1.7-2.303) | 1.942(1.675-2.251) | 1.881(1.632-2.169) | 1.797(1.57-2.057) | 1.69(1.492-1.916) | 1.564(1.398-1.75) | 1.423(1.291-1.567) | 1.28(1.181-1.387) | 1.155(1.084-1.231) | 1.062(1.014-1.112) | 1.009(0.985-1.033) | 1.004(0.994-1.014) | 1.048(0.992-1.108) | 1.116(1.011-1.231) | 1.175(1.036-1.332) | 1.192(1.041-1.365) | 1.165(0.997-1.36) |
| lag18 | 2.115(1.807-2.477) | 2.109(1.802-2.467) | 2.06(1.768-2.4) | 1.985(1.713-2.3) | 1.884(1.638-2.167) | 1.76(1.546-2.003) | 1.615(1.438-1.814) | 1.456(1.317-1.609) | 1.297(1.194-1.409) | 1.161(1.087-1.24) | 1.061(1.012-1.113) | 1.006(0.982-1.031) | 1.006(0.995-1.016) | 1.059(1.001-1.121) | 1.139(1.03-1.26) | 1.207(1.061-1.373) | 1.222(1.063-1.404) | 1.182(1.007-1.388) |
| lag19 | 2.258(1.918-2.658) | 2.248(1.911-2.644) | 2.184(1.864-2.558) | 2.091(1.795-2.435) | 1.971(1.706-2.278) | 1.827(1.598-2.089) | 1.663(1.474-1.875) | 1.485(1.339-1.647) | 1.312(1.204-1.429) | 1.165(1.088-1.247) | 1.059(1.008-1.113) | 1.003(0.979-1.029) | 1.008(0.997-1.019) | 1.071(1.011-1.136) | 1.163(1.049-1.29) | 1.24(1.086-1.415) | 1.252(1.086-1.444) | 1.199(1.016-1.415) |
| lag20 | 2.41(2.035-2.855) | 2.397(2.026-2.837) | 2.312(1.963-2.724) | 2.198(1.876-2.575) | 2.056(1.77-2.389) | 1.891(1.645-2.173) | 1.707(1.506-1.934) | 1.511(1.356-1.683) | 1.323(1.21-1.446) | 1.166(1.087-1.252) | 1.056(1.004-1.111) | 1(0.975-1.026) | 1.01(0.998-1.021) | 1.084(1.021-1.151) | 1.189(1.07-1.322) | 1.275(1.113-1.46) | 1.283(1.108-1.486) | 1.214(1.023-1.441) |
| lag21 | 2.575(2.157-3.073) | 2.556(2.144-3.048) | 2.446(2.06-2.903) | 2.306(1.954-2.721) | 2.139(1.828-2.503) | 1.95(1.686-2.257) | 1.746(1.531-1.99) | 1.532(1.368-1.715) | 1.33(1.212-1.46) | 1.166(1.083-1.255) | 1.052(0.997-1.109) | 0.996(0.97-1.023) | 1.012(1-1.023) | 1.098(1.032-1.168) | 1.217(1.09-1.358) | 1.311(1.138-1.509) | 1.314(1.127-1.532) | 1.227(1.025-1.469) |

| Table S13 Risk of female DV with each 1 °C change in lag time from 13.8 °C to 30 °C | | | | | | | | | | | | | | | | | | |
| --- | --- | --- | --- | --- | --- | --- | --- | --- | --- | --- | --- | --- | --- | --- | --- | --- | --- | --- |
| Lag(days) | Temperature(℃), RR(95%CI) | | | | | | | | | | | | | | | | | |
|  | 13.8 | 14 | 15 | 16 | 17 | 18 | 19 | 20 | 21 | 22 | 23 | 24 | 25 | 26 | 27 | 28 | 29 | 30 |
| lag0 | 0.859(0.779-0.946) | 0.864(0.785-0.952) | 0.892(0.813-0.978) | 0.916(0.839-1.001) | 0.938(0.864-1.019) | 0.956(0.886-1.031) | 0.97(0.906-1.038) | 0.979(0.923-1.038) | 0.984(0.937-1.033) | 0.986(0.949-1.025) | 0.989(0.962-1.017) | 0.994(0.98-1.008) | 1.003(0.997-1.009) | 1.016(0.984-1.049) | 1.031(0.973-1.092) | 1.043(0.965-1.127) | 1.05(0.959-1.15) | 1.052(0.945-1.172) |
| lag1 | 0.787(0.723-0.858) | 0.795(0.73-0.865) | 0.829(0.763-0.9) | 0.86(0.795-0.931) | 0.889(0.824-0.958) | 0.913(0.851-0.979) | 0.932(0.875-0.993) | 0.946(0.896-1) | 0.956(0.913-1.001) | 0.964(0.929-1) | 0.973(0.946-1) | 0.986(0.972-1) | 1.006(1-1.012) | 1.034(1.001-1.068) | 1.063(1.002-1.128) | 1.085(1.004-1.174) | 1.093(0.999-1.195) | 1.087(0.978-1.207) |
| lag2 | 0.807(0.746-0.874) | 0.815(0.754-0.882) | 0.853(0.791-0.921) | 0.888(0.825-0.956) | 0.919(0.857-0.986) | 0.945(0.886-1.009) | 0.965(0.91-1.023) | 0.978(0.93-1.029) | 0.985(0.944-1.028) | 0.989(0.955-1.023) | 0.991(0.966-1.016) | 0.995(0.982-1.008) | 1.003(0.997-1.008) | 1.015(0.984-1.047) | 1.03(0.974-1.089) | 1.042(0.968-1.121) | 1.047(0.964-1.138) | 1.047(0.95-1.154) |
| lag3 | 0.848(0.779-0.923) | 0.856(0.786-0.932) | 0.894(0.824-0.971) | 0.929(0.858-1.006) | 0.958(0.888-1.033) | 0.981(0.915-1.053) | 0.997(0.936-1.063) | 1.005(0.952-1.062) | 1.006(0.961-1.054) | 1.003(0.967-1.042) | 1(0.972-1.028) | 0.998(0.984-1.012) | 1.002(0.995-1.008) | 1.011(0.978-1.046) | 1.023(0.963-1.086) | 1.032(0.953-1.117) | 1.034(0.947-1.13) | 1.03(0.931-1.14) |
| lag4 | 0.897(0.822-0.98) | 0.905(0.829-0.988) | 0.941(0.864-1.025) | 0.972(0.895-1.056) | 0.998(0.922-1.079) | 1.016(0.944-1.093) | 1.026(0.961-1.096) | 1.028(0.97-1.089) | 1.022(0.974-1.073) | 1.013(0.974-1.053) | 1.004(0.975-1.034) | 0.999(0.984-1.014) | 1.002(0.995-1.008) | 1.012(0.977-1.049) | 1.026(0.962-1.094) | 1.035(0.952-1.125) | 1.033(0.943-1.133) | 1.022(0.921-1.134) |
| lag5 | 0.952(0.865-1.048) | 0.959(0.872-1.055) | 0.992(0.904-1.088) | 1.018(0.931-1.114) | 1.039(0.953-1.132) | 1.051(0.971-1.139) | 1.056(0.982-1.135) | 1.051(0.987-1.119) | 1.039(0.985-1.095) | 1.024(0.981-1.069) | 1.01(0.978-1.042) | 1.001(0.984-1.018) | 1.001(0.994-1.008) | 1.011(0.972-1.051) | 1.023(0.954-1.097) | 1.03(0.941-1.128) | 1.025(0.929-1.132) | 1.01(0.903-1.129) |
| lag6 | 1.012(0.914-1.121) | 1.018(0.92-1.127) | 1.046(0.947-1.155) | 1.068(0.97-1.176) | 1.083(0.988-1.186) | 1.09(1.001-1.186) | 1.088(1.008-1.175) | 1.077(1.008-1.151) | 1.059(1.001-1.12) | 1.038(0.992-1.086) | 1.018(0.985-1.053) | 1.005(0.987-1.022) | 1(0.992-1.007) | 1.005(0.965-1.047) | 1.013(0.941-1.09) | 1.016(0.925-1.117) | 1.009(0.911-1.119) | 0.992(0.883-1.115) |
| lag7 | 1.076(0.966-1.199) | 1.082(0.971-1.205) | 1.105(0.995-1.228) | 1.122(1.014-1.242) | 1.132(1.027-1.246) | 1.132(1.035-1.238) | 1.124(1.037-1.219) | 1.107(1.032-1.187) | 1.082(1.02-1.147) | 1.054(1.006-1.105) | 1.029(0.993-1.065) | 1.009(0.991-1.027) | 0.998(0.991-1.006) | 0.998(0.956-1.041) | 1.001(0.928-1.08) | 1.001(0.907-1.104) | 0.992(0.892-1.103) | 0.974(0.863-1.099) |
| lag8 | 1.146(1.021-1.286) | 1.15(1.026-1.29) | 1.17(1.046-1.309) | 1.182(1.061-1.317) | 1.186(1.07-1.314) | 1.18(1.073-1.298) | 1.165(1.069-1.269) | 1.14(1.058-1.227) | 1.107(1.04-1.178) | 1.072(1.02-1.127) | 1.039(1.002-1.078) | 1.013(0.994-1.032) | 0.997(0.989-1.005) | 0.991(0.948-1.037) | 0.99(0.914-1.073) | 0.987(0.891-1.094) | 0.977(0.874-1.092) | 0.958(0.844-1.088) |
| lag9 | 1.22(1.08-1.38) | 1.225(1.084-1.384) | 1.241(1.102-1.398) | 1.249(1.113-1.401) | 1.246(1.117-1.39) | 1.234(1.115-1.365) | 1.21(1.105-1.325) | 1.176(1.087-1.272) | 1.134(1.062-1.211) | 1.09(1.034-1.149) | 1.049(1.009-1.091) | 1.017(0.996-1.037) | 0.996(0.987-1.005) | 0.987(0.942-1.035) | 0.984(0.905-1.071) | 0.979(0.879-1.091) | 0.967(0.86-1.087) | 0.947(0.828-1.083) |
| lag10 | 1.301(1.143-1.481) | 1.305(1.147-1.484) | 1.319(1.163-1.495) | 1.322(1.171-1.493) | 1.314(1.171-1.474) | 1.293(1.163-1.439) | 1.261(1.145-1.387) | 1.215(1.119-1.32) | 1.162(1.084-1.245) | 1.108(1.048-1.171) | 1.058(1.016-1.102) | 1.019(0.998-1.041) | 0.995(0.986-1.005) | 0.986(0.938-1.036) | 0.983(0.9-1.074) | 0.979(0.874-1.096) | 0.964(0.853-1.09) | 0.941(0.818-1.083) |
| lag11 | 1.388(1.212-1.589) | 1.391(1.216-1.592) | 1.403(1.231-1.601) | 1.403(1.236-1.592) | 1.388(1.231-1.565) | 1.359(1.216-1.519) | 1.315(1.19-1.453) | 1.257(1.153-1.371) | 1.191(1.108-1.28) | 1.124(1.061-1.191) | 1.066(1.022-1.112) | 1.021(0.999-1.044) | 0.995(0.986-1.005) | 0.988(0.938-1.04) | 0.988(0.901-1.083) | 0.985(0.876-1.107) | 0.968(0.853-1.099) | 0.94(0.813-1.086) |
| lag12 | 1.481(1.286-1.705) | 1.484(1.29-1.708) | 1.495(1.304-1.714) | 1.49(1.306-1.7) | 1.468(1.295-1.664) | 1.429(1.273-1.605) | 1.373(1.237-1.523) | 1.301(1.189-1.423) | 1.219(1.132-1.314) | 1.14(1.074-1.211) | 1.072(1.026-1.121) | 1.022(0.999-1.046) | 0.996(0.986-1.006) | 0.992(0.94-1.046) | 0.997(0.907-1.096) | 0.997(0.883-1.125) | 0.978(0.858-1.115) | 0.942(0.811-1.094) |
| lag13 | 1.58(1.365-1.83) | 1.584(1.369-1.833) | 1.593(1.382-1.837) | 1.583(1.38-1.816) | 1.553(1.364-1.769) | 1.503(1.332-1.695) | 1.433(1.286-1.597) | 1.345(1.225-1.477) | 1.248(1.154-1.348) | 1.155(1.085-1.229) | 1.077(1.029-1.128) | 1.022(0.998-1.047) | 0.996(0.986-1.007) | 0.998(0.944-1.055) | 1.01(0.916-1.114) | 1.014(0.894-1.149) | 0.992(0.867-1.136) | 0.948(0.812-1.106) |
| lag14 | 1.687(1.448-1.966) | 1.691(1.453-1.969) | 1.698(1.464-1.969) | 1.682(1.458-1.94) | 1.642(1.434-1.881) | 1.58(1.393-1.791) | 1.494(1.335-1.672) | 1.389(1.26-1.531) | 1.275(1.175-1.382) | 1.168(1.095-1.246) | 1.081(1.031-1.133) | 1.022(0.997-1.047) | 0.997(0.987-1.008) | 1.006(0.95-1.066) | 1.027(0.927-1.137) | 1.035(0.909-1.179) | 1.011(0.878-1.163) | 0.955(0.813-1.122) |
| lag15 | 1.802(1.537-2.113) | 1.806(1.541-2.115) | 1.808(1.55-2.11) | 1.785(1.539-2.072) | 1.735(1.507-1.998) | 1.658(1.454-1.889) | 1.555(1.383-1.749) | 1.431(1.293-1.584) | 1.3(1.194-1.414) | 1.179(1.102-1.262) | 1.083(1.031-1.138) | 1.02(0.995-1.047) | 0.999(0.988-1.01) | 1.016(0.957-1.078) | 1.046(0.941-1.163) | 1.06(0.926-1.214) | 1.032(0.892-1.194) | 0.965(0.816-1.141) |
| lag16 | 1.925(1.632-2.271) | 1.928(1.636-2.273) | 1.925(1.64-2.26) | 1.893(1.621-2.209) | 1.829(1.579-2.118) | 1.736(1.515-1.989) | 1.615(1.429-1.825) | 1.472(1.324-1.636) | 1.322(1.211-1.444) | 1.188(1.107-1.275) | 1.084(1.029-1.141) | 1.018(0.992-1.046) | 1(0.989-1.012) | 1.027(0.965-1.093) | 1.068(0.957-1.192) | 1.089(0.946-1.253) | 1.056(0.908-1.229) | 0.976(0.82-1.162) |
| lag17 | 2.057(1.733-2.442) | 2.059(1.736-2.442) | 2.048(1.734-2.418) | 2.003(1.706-2.352) | 1.924(1.652-2.241) | 1.813(1.574-2.088) | 1.673(1.473-1.899) | 1.51(1.353-1.685) | 1.342(1.225-1.471) | 1.195(1.111-1.285) | 1.083(1.027-1.142) | 1.016(0.988-1.044) | 1.002(0.99-1.014) | 1.039(0.975-1.108) | 1.092(0.975-1.224) | 1.12(0.968-1.295) | 1.083(0.925-1.267) | 0.988(0.825-1.184) |
| lag18 | 2.198(1.841-2.625) | 2.198(1.842-2.623) | 2.175(1.831-2.584) | 2.116(1.792-2.498) | 2.019(1.725-2.364) | 1.888(1.631-2.185) | 1.727(1.514-1.97) | 1.544(1.378-1.729) | 1.359(1.237-1.494) | 1.199(1.112-1.293) | 1.081(1.023-1.142) | 1.013(0.984-1.041) | 1.004(0.992-1.017) | 1.052(0.985-1.124) | 1.119(0.995-1.257) | 1.154(0.993-1.34) | 1.111(0.945-1.306) | 1(0.83-1.206) |
| lag19 | 2.349(1.956-2.822) | 2.346(1.955-2.816) | 2.308(1.932-2.758) | 2.23(1.878-2.648) | 2.112(1.794-2.487) | 1.96(1.684-2.28) | 1.777(1.551-2.036) | 1.573(1.399-1.77) | 1.372(1.244-1.513) | 1.201(1.111-1.299) | 1.077(1.018-1.14) | 1.009(0.98-1.038) | 1.006(0.994-1.019) | 1.066(0.997-1.141) | 1.146(1.017-1.292) | 1.189(1.02-1.386) | 1.141(0.966-1.346) | 1.013(0.835-1.228) |
| lag20 | 2.511(2.076-3.037) | 2.504(2.072-3.027) | 2.445(2.033-2.941) | 2.344(1.961-2.802) | 2.203(1.86-2.61) | 2.026(1.732-2.372) | 1.822(1.581-2.098) | 1.598(1.414-1.806) | 1.381(1.248-1.529) | 1.201(1.108-1.302) | 1.072(1.011-1.137) | 1.005(0.975-1.035) | 1.009(0.996-1.022) | 1.081(1.009-1.159) | 1.176(1.04-1.33) | 1.227(1.048-1.437) | 1.171(0.987-1.39) | 1.025(0.839-1.252) |
| lag21 | 2.684(2.2-3.275) | 2.672(2.192-3.258) | 2.587(2.132-3.138) | 2.458(2.04-2.963) | 2.29(1.918-2.735) | 2.088(1.771-2.462) | 1.86(1.604-2.158) | 1.618(1.423-1.839) | 1.387(1.247-1.542) | 1.198(1.101-1.303) | 1.066(1.003-1.133) | 1(0.969-1.032) | 1.011(0.998-1.025) | 1.097(1.021-1.179) | 1.207(1.062-1.372) | 1.267(1.076-1.493) | 1.204(1.007-1.439) | 1.037(0.841-1.28) |

| Table S14 Risk of male DN with each 1 °C change in lag time from 13.8 °C to 30 °C | | | | | | | | | | | | | | | | | | |
| --- | --- | --- | --- | --- | --- | --- | --- | --- | --- | --- | --- | --- | --- | --- | --- | --- | --- | --- |
| Lag(days) | Temperature(℃)(male), RR(95%CI) | | | | | | | | | | | | | | | | | |
|  | 13.8 | 14 | 15 | 16 | 17 | 18 | 19 | 20 | 21 | 22 | 23 | 24 | 25 | 26 | 27 | 28 | 29 | 30 |
| lag0 | 0.847(0.796-0.901) | 0.85(0.8-0.904) | 0.866(0.817-0.918) | 0.883(0.836-0.934) | 0.9(0.855-0.949) | 0.918(0.875-0.963) | 0.936(0.897-0.977) | 0.955(0.921-0.99) | 0.973(0.945-1.001) | 0.988(0.967-1.01) | 0.999(0.984-1.014) | 1.003(0.996-1.01) | 0.998(0.995-1.001) | 0.985(0.969-1.001) | 0.971(0.942-1.001) | 0.965(0.926-1.005) | 0.971(0.927-1.017) | 0.99(0.94-1.044) |
| lag1 | 0.83(0.779-0.884) | 0.834(0.783-0.888) | 0.853(0.803-0.906) | 0.872(0.824-0.924) | 0.892(0.845-0.941) | 0.911(0.867-0.958) | 0.931(0.89-0.973) | 0.95(0.915-0.986) | 0.969(0.94-0.998) | 0.984(0.962-1.007) | 0.996(0.981-1.012) | 1.001(0.994-1.009) | 0.998(0.995-1.002) | 0.988(0.971-1.006) | 0.977(0.947-1.009) | 0.971(0.931-1.013) | 0.975(0.93-1.024) | 0.99(0.937-1.045) |
| lag2 | 0.85(0.798-0.906) | 0.854(0.802-0.909) | 0.872(0.821-0.925) | 0.889(0.84-0.942) | 0.906(0.858-0.957) | 0.923(0.878-0.97) | 0.939(0.898-0.982) | 0.954(0.919-0.991) | 0.969(0.94-0.999) | 0.981(0.959-1.004) | 0.991(0.976-1.007) | 0.998(0.99-1.006) | 1(0.997-1.003) | 0.999(0.981-1.017) | 0.995(0.963-1.029) | 0.993(0.951-1.037) | 0.994(0.947-1.043) | 0.998(0.946-1.052) |
| lag3 | 0.86(0.804-0.92) | 0.864(0.808-0.923) | 0.88(0.825-0.938) | 0.895(0.842-0.953) | 0.911(0.859-0.966) | 0.925(0.876-0.977) | 0.939(0.895-0.986) | 0.952(0.914-0.992) | 0.964(0.933-0.996) | 0.975(0.952-1) | 0.985(0.969-1.002) | 0.994(0.986-1.003) | 1.002(0.998-1.005) | 1.008(0.988-1.028) | 1.012(0.977-1.049) | 1.015(0.968-1.063) | 1.015(0.964-1.068) | 1.013(0.958-1.07) |
| lag4 | 0.861(0.801-0.925) | 0.863(0.804-0.927) | 0.878(0.82-0.94) | 0.892(0.836-0.953) | 0.906(0.852-0.964) | 0.92(0.868-0.974) | 0.932(0.886-0.981) | 0.945(0.905-0.986) | 0.956(0.924-0.99) | 0.968(0.943-0.993) | 0.979(0.962-0.996) | 0.991(0.982-0.999) | 1.003(1-1.007) | 1.016(0.996-1.037) | 1.027(0.99-1.066) | 1.034(0.986-1.086) | 1.036(0.983-1.091) | 1.032(0.975-1.092) |
| lag5 | 0.856(0.792-0.924) | 0.859(0.795-0.927) | 0.872(0.81-0.938) | 0.885(0.825-0.95) | 0.898(0.84-0.96) | 0.911(0.856-0.969) | 0.923(0.874-0.975) | 0.935(0.893-0.979) | 0.947(0.913-0.982) | 0.959(0.933-0.986) | 0.973(0.955-0.991) | 0.988(0.979-0.997) | 1.005(1.001-1.009) | 1.023(1.002-1.046) | 1.04(1.001-1.082) | 1.052(1-1.107) | 1.056(1-1.116) | 1.053(0.993-1.116) |
| lag6 | 0.85(0.782-0.924) | 0.852(0.785-0.926) | 0.865(0.798-0.937) | 0.877(0.812-0.947) | 0.889(0.827-0.956) | 0.901(0.843-0.964) | 0.913(0.86-0.969) | 0.926(0.88-0.973) | 0.938(0.901-0.976) | 0.952(0.924-0.98) | 0.967(0.948-0.986) | 0.985(0.975-0.994) | 1.006(1.002-1.01) | 1.03(1.006-1.054) | 1.052(1.01-1.097) | 1.069(1.013-1.128) | 1.076(1.015-1.14) | 1.073(1.008-1.142) |
| lag7 | 0.845(0.772-0.925) | 0.848(0.775-0.927) | 0.859(0.787-0.936) | 0.87(0.8-0.945) | 0.881(0.815-0.953) | 0.893(0.83-0.96) | 0.905(0.848-0.965) | 0.917(0.869-0.968) | 0.93(0.891-0.971) | 0.945(0.915-0.975) | 0.962(0.941-0.983) | 0.982(0.972-0.992) | 1.007(1.002-1.011) | 1.036(1.01-1.061) | 1.063(1.017-1.111) | 1.084(1.024-1.148) | 1.094(1.028-1.163) | 1.092(1.022-1.166) |
| lag8 | 0.842(0.765-0.928) | 0.844(0.767-0.929) | 0.854(0.779-0.937) | 0.865(0.791-0.945) | 0.875(0.805-0.952) | 0.887(0.82-0.958) | 0.898(0.838-0.962) | 0.911(0.859-0.965) | 0.924(0.882-0.967) | 0.939(0.908-0.971) | 0.957(0.936-0.979) | 0.98(0.969-0.991) | 1.008(1.003-1.013) | 1.041(1.014-1.068) | 1.073(1.024-1.124) | 1.098(1.034-1.166) | 1.11(1.04-1.183) | 1.109(1.035-1.188) |
| lag9 | 0.841(0.759-0.932) | 0.843(0.761-0.933) | 0.852(0.772-0.939) | 0.861(0.784-0.946) | 0.871(0.797-0.952) | 0.882(0.812-0.957) | 0.893(0.83-0.961) | 0.905(0.851-0.963) | 0.919(0.875-0.964) | 0.934(0.902-0.968) | 0.954(0.931-0.977) | 0.978(0.967-0.989) | 1.009(1.004-1.014) | 1.045(1.017-1.073) | 1.081(1.03-1.134) | 1.11(1.042-1.181) | 1.124(1.052-1.201) | 1.124(1.047-1.206) |
| lag10 | 0.841(0.755-0.937) | 0.843(0.757-0.938) | 0.851(0.767-0.943) | 0.859(0.778-0.949) | 0.869(0.791-0.954) | 0.879(0.806-0.958) | 0.89(0.824-0.96) | 0.901(0.845-0.961) | 0.915(0.869-0.962) | 0.931(0.897-0.966) | 0.951(0.927-0.975) | 0.976(0.965-0.988) | 1.009(1.004-1.014) | 1.049(1.02-1.078) | 1.088(1.035-1.144) | 1.12(1.05-1.194) | 1.136(1.061-1.216) | 1.137(1.057-1.223) |
| lag11 | 0.843(0.753-0.943) | 0.844(0.755-0.944) | 0.851(0.764-0.948) | 0.859(0.774-0.953) | 0.867(0.786-0.957) | 0.877(0.801-0.96) | 0.887(0.819-0.962) | 0.899(0.84-0.962) | 0.912(0.865-0.962) | 0.928(0.892-0.965) | 0.949(0.924-0.973) | 0.975(0.963-0.987) | 1.01(1.005-1.015) | 1.052(1.022-1.082) | 1.094(1.039-1.151) | 1.128(1.056-1.205) | 1.146(1.069-1.229) | 1.149(1.066-1.238) |
| lag12 | 0.845(0.751-0.951) | 0.846(0.753-0.952) | 0.853(0.761-0.955) | 0.86(0.771-0.959) | 0.867(0.782-0.962) | 0.876(0.797-0.964) | 0.886(0.814-0.964) | 0.897(0.836-0.963) | 0.91(0.861-0.962) | 0.926(0.889-0.965) | 0.947(0.922-0.973) | 0.974(0.962-0.987) | 1.01(1.005-1.016) | 1.054(1.023-1.085) | 1.098(1.041-1.158) | 1.134(1.06-1.215) | 1.155(1.074-1.241) | 1.159(1.073-1.252) |
| lag13 | 0.849(0.75-0.961) | 0.85(0.751-0.961) | 0.855(0.759-0.963) | 0.861(0.768-0.966) | 0.868(0.779-0.968) | 0.876(0.793-0.968) | 0.886(0.811-0.968) | 0.896(0.832-0.966) | 0.909(0.857-0.964) | 0.925(0.886-0.966) | 0.946(0.92-0.973) | 0.974(0.961-0.987) | 1.01(1.005-1.016) | 1.055(1.023-1.088) | 1.101(1.042-1.163) | 1.139(1.061-1.222) | 1.161(1.078-1.251) | 1.167(1.077-1.264) |
| lag14 | 0.854(0.75-0.972) | 0.854(0.751-0.972) | 0.859(0.758-0.973) | 0.864(0.766-0.974) | 0.87(0.777-0.975) | 0.878(0.791-0.974) | 0.886(0.808-0.973) | 0.897(0.829-0.969) | 0.909(0.855-0.966) | 0.925(0.885-0.967) | 0.946(0.918-0.974) | 0.974(0.96-0.988) | 1.011(1.005-1.016) | 1.055(1.022-1.09) | 1.102(1.041-1.167) | 1.141(1.061-1.228) | 1.166(1.079-1.259) | 1.174(1.081-1.276) |
| lag15 | 0.859(0.75-0.984) | 0.86(0.751-0.983) | 0.863(0.758-0.983) | 0.867(0.765-0.983) | 0.873(0.775-0.983) | 0.88(0.789-0.981) | 0.888(0.806-0.978) | 0.898(0.827-0.974) | 0.91(0.853-0.97) | 0.926(0.883-0.97) | 0.946(0.918-0.976) | 0.974(0.96-0.988) | 1.01(1.004-1.017) | 1.055(1.021-1.09) | 1.102(1.038-1.169) | 1.142(1.058-1.232) | 1.168(1.078-1.266) | 1.18(1.083-1.286) |
| lag16 | 0.865(0.752-0.996) | 0.866(0.753-0.996) | 0.868(0.758-0.994) | 0.871(0.765-0.993) | 0.876(0.775-0.991) | 0.882(0.788-0.989) | 0.89(0.805-0.985) | 0.9(0.826-0.979) | 0.911(0.853-0.974) | 0.927(0.883-0.973) | 0.947(0.918-0.978) | 0.975(0.96-0.989) | 1.01(1.004-1.017) | 1.054(1.018-1.09) | 1.1(1.035-1.169) | 1.14(1.055-1.233) | 1.169(1.076-1.269) | 1.184(1.083-1.295) |
| lag17 | 0.873(0.754-1.01) | 0.873(0.755-1.009) | 0.874(0.759-1.006) | 0.876(0.766-1.003) | 0.88(0.775-1) | 0.886(0.787-0.997) | 0.893(0.804-0.992) | 0.902(0.826-0.985) | 0.914(0.853-0.979) | 0.929(0.883-0.977) | 0.949(0.918-0.981) | 0.975(0.96-0.991) | 1.01(1.003-1.016) | 1.052(1.016-1.089) | 1.096(1.03-1.167) | 1.137(1.049-1.232) | 1.168(1.073-1.271) | 1.188(1.083-1.302) |
| lag18 | 0.88(0.757-1.024) | 0.88(0.758-1.023) | 0.88(0.761-1.018) | 0.882(0.767-1.014) | 0.885(0.775-1.01) | 0.89(0.787-1.006) | 0.897(0.804-1) | 0.905(0.826-0.992) | 0.917(0.853-0.985) | 0.932(0.884-0.982) | 0.951(0.919-0.984) | 0.977(0.961-0.992) | 1.009(1.003-1.016) | 1.049(1.012-1.087) | 1.091(1.023-1.163) | 1.131(1.042-1.228) | 1.164(1.068-1.27) | 1.19(1.083-1.308) |
| lag19 | 0.889(0.76-1.039) | 0.888(0.761-1.038) | 0.887(0.763-1.031) | 0.888(0.768-1.026) | 0.89(0.776-1.021) | 0.894(0.788-1.015) | 0.901(0.805-1.008) | 0.909(0.827-1) | 0.921(0.855-0.992) | 0.935(0.886-0.987) | 0.954(0.921-0.988) | 0.978(0.962-0.995) | 1.009(1.002-1.015) | 1.045(1.007-1.084) | 1.084(1.016-1.158) | 1.124(1.033-1.222) | 1.159(1.061-1.267) | 1.191(1.08-1.312) |
| lag20 | 0.898(0.764-1.056) | 0.897(0.764-1.054) | 0.895(0.766-1.046) | 0.895(0.77-1.04) | 0.896(0.777-1.033) | 0.9(0.789-1.026) | 0.905(0.805-1.018) | 0.914(0.828-1.009) | 0.925(0.856-1) | 0.939(0.888-0.994) | 0.958(0.923-0.993) | 0.98(0.964-0.997) | 1.008(1.001-1.015) | 1.04(1.001-1.08) | 1.076(1.006-1.151) | 1.114(1.022-1.215) | 1.152(1.052-1.263) | 1.191(1.076-1.317) |
| lag21 | 0.908(0.767-1.075) | 0.907(0.767-1.073) | 0.903(0.768-1.063) | 0.902(0.771-1.055) | 0.902(0.778-1.047) | 0.905(0.789-1.039) | 0.911(0.806-1.03) | 0.919(0.829-1.019) | 0.93(0.858-1.009) | 0.944(0.89-1.002) | 0.962(0.926-1) | 0.982(0.965-1) | 1.007(0.999-1.014) | 1.035(0.994-1.076) | 1.066(0.994-1.144) | 1.103(1.008-1.207) | 1.144(1.039-1.259) | 1.19(1.069-1.323) |

| Table S15 Risk of female DN with each 1 °C change in lag time from 13.8 °C to 30 °C | | | | | | | | | | | | | | | | | | |
| --- | --- | --- | --- | --- | --- | --- | --- | --- | --- | --- | --- | --- | --- | --- | --- | --- | --- | --- |
| Lag(days) | Temperature(℃)(female), RR(95%CI) | | | | | | | | | | | | | | | | | |
|  | 13.8 | 14 | 15 | 16 | 17 | 18 | 19 | 20 | 21 | 22 | 23 | 24 | 25 | 26 | 27 | 28 | 29 | 30 |
| lag0 | 0.9(0.842-0.962) | 0.904(0.846-0.966) | 0.925(0.869-0.985) | 0.944(0.889-1.002) | 0.962(0.909-1.017) | 0.977(0.927-1.029) | 0.989(0.944-1.036) | 0.999(0.96-1.039) | 1.006(0.974-1.038) | 1.009(0.985-1.033) | 1.009(0.993-1.025) | 1.005(0.997-1.013) | 0.998(0.994-1.001) | 0.987(0.97-1.005) | 0.977(0.945-1.009) | 0.968(0.926-1.011) | 0.963(0.916-1.013) | 0.962(0.909-1.019) |
| lag1 | 0.86(0.803-0.922) | 0.865(0.808-0.926) | 0.89(0.833-0.95) | 0.913(0.858-0.971) | 0.934(0.881-0.99) | 0.953(0.903-1.006) | 0.969(0.924-1.017) | 0.983(0.944-1.024) | 0.994(0.962-1.026) | 1(0.976-1.025) | 1.004(0.987-1.021) | 1.003(0.995-1.011) | 0.998(0.995-1.002) | 0.99(0.972-1.009) | 0.98(0.947-1.014) | 0.971(0.927-1.016) | 0.963(0.914-1.015) | 0.958(0.903-1.015) |
| lag2 | 0.843(0.787-0.903) | 0.847(0.792-0.907) | 0.87(0.815-0.929) | 0.892(0.838-0.95) | 0.913(0.86-0.968) | 0.931(0.882-0.983) | 0.948(0.903-0.995) | 0.963(0.924-1.003) | 0.975(0.944-1.008) | 0.985(0.961-1.01) | 0.993(0.976-1.01) | 0.998(0.99-1.006) | 1(0.997-1.004) | 1(0.981-1.02) | 0.997(0.963-1.033) | 0.991(0.946-1.039) | 0.982(0.932-1.035) | 0.97(0.916-1.027) |
| lag3 | 0.829(0.77-0.893) | 0.834(0.775-0.897) | 0.855(0.797-0.917) | 0.875(0.818-0.936) | 0.895(0.84-0.954) | 0.913(0.861-0.968) | 0.93(0.882-0.98) | 0.945(0.904-0.988) | 0.958(0.925-0.993) | 0.97(0.945-0.997) | 0.982(0.964-1) | 0.992(0.983-1.001) | 1.003(0.999-1.007) | 1.012(0.991-1.034) | 1.018(0.98-1.058) | 1.018(0.968-1.07) | 1.008(0.954-1.065) | 0.99(0.933-1.051) |
| lag4 | 0.818(0.757-0.885) | 0.822(0.761-0.889) | 0.842(0.782-0.908) | 0.861(0.802-0.925) | 0.88(0.822-0.942) | 0.897(0.843-0.955) | 0.914(0.864-0.966) | 0.929(0.886-0.974) | 0.943(0.908-0.979) | 0.957(0.93-0.984) | 0.971(0.953-0.99) | 0.987(0.978-0.996) | 1.005(1.001-1.009) | 1.025(1.002-1.047) | 1.04(1-1.082) | 1.046(0.993-1.101) | 1.036(0.979-1.096) | 1.013(0.954-1.076) |
| lag5 | 0.809(0.744-0.88) | 0.813(0.748-0.884) | 0.832(0.768-0.901) | 0.85(0.787-0.918) | 0.867(0.806-0.933) | 0.884(0.827-0.945) | 0.9(0.848-0.955) | 0.915(0.87-0.962) | 0.929(0.893-0.967) | 0.944(0.917-0.973) | 0.961(0.942-0.981) | 0.982(0.972-0.991) | 1.007(1.003-1.011) | 1.036(1.012-1.06) | 1.061(1.018-1.106) | 1.073(1.016-1.133) | 1.064(1.003-1.128) | 1.036(0.973-1.104) |
| lag6 | 0.802(0.733-0.879) | 0.806(0.736-0.882) | 0.823(0.754-0.898) | 0.84(0.773-0.913) | 0.857(0.791-0.927) | 0.873(0.811-0.939) | 0.888(0.832-0.947) | 0.903(0.855-0.953) | 0.917(0.878-0.958) | 0.933(0.904-0.964) | 0.953(0.932-0.974) | 0.977(0.967-0.988) | 1.009(1.005-1.014) | 1.046(1.021-1.073) | 1.08(1.033-1.129) | 1.098(1.035-1.163) | 1.089(1.023-1.16) | 1.058(0.99-1.131) |
| lag7 | 0.798(0.723-0.88) | 0.801(0.726-0.883) | 0.816(0.743-0.897) | 0.832(0.76-0.911) | 0.848(0.778-0.923) | 0.863(0.797-0.934) | 0.878(0.818-0.941) | 0.892(0.841-0.946) | 0.907(0.866-0.95) | 0.924(0.893-0.956) | 0.946(0.924-0.968) | 0.974(0.963-0.985) | 1.011(1.006-1.016) | 1.055(1.028-1.083) | 1.096(1.045-1.149) | 1.118(1.051-1.19) | 1.111(1.04-1.187) | 1.076(1.003-1.155) |
| lag8 | 0.794(0.715-0.882) | 0.797(0.718-0.885) | 0.811(0.734-0.897) | 0.826(0.749-0.909) | 0.84(0.766-0.92) | 0.854(0.785-0.929) | 0.869(0.806-0.936) | 0.883(0.829-0.94) | 0.898(0.855-0.944) | 0.916(0.883-0.95) | 0.94(0.917-0.963) | 0.971(0.959-0.982) | 1.012(1.007-1.017) | 1.062(1.033-1.092) | 1.109(1.055-1.165) | 1.136(1.064-1.212) | 1.129(1.053-1.21) | 1.091(1.014-1.175) |
| lag9 | 0.792(0.709-0.886) | 0.795(0.712-0.888) | 0.807(0.726-0.898) | 0.82(0.741-0.909) | 0.834(0.757-0.918) | 0.847(0.775-0.926) | 0.861(0.795-0.932) | 0.875(0.819-0.935) | 0.891(0.845-0.939) | 0.91(0.875-0.945) | 0.935(0.911-0.959) | 0.968(0.956-0.98) | 1.013(1.008-1.018) | 1.068(1.037-1.099) | 1.119(1.062-1.178) | 1.149(1.075-1.229) | 1.143(1.064-1.228) | 1.103(1.022-1.19) |
| lag10 | 0.792(0.704-0.89) | 0.794(0.707-0.892) | 0.805(0.719-0.9) | 0.816(0.733-0.909) | 0.828(0.748-0.917) | 0.841(0.766-0.924) | 0.854(0.786-0.928) | 0.868(0.81-0.931) | 0.884(0.837-0.934) | 0.904(0.869-0.942) | 0.931(0.906-0.956) | 0.966(0.954-0.979) | 1.014(1.008-1.019) | 1.072(1.041-1.105) | 1.127(1.068-1.189) | 1.16(1.082-1.243) | 1.154(1.073-1.242) | 1.113(1.029-1.203) |
| lag11 | 0.792(0.701-0.896) | 0.794(0.703-0.897) | 0.803(0.714-0.904) | 0.813(0.726-0.91) | 0.824(0.74-0.917) | 0.836(0.757-0.922) | 0.849(0.777-0.926) | 0.863(0.802-0.928) | 0.879(0.83-0.931) | 0.9(0.863-0.939) | 0.927(0.902-0.954) | 0.964(0.952-0.977) | 1.014(1.009-1.02) | 1.075(1.043-1.109) | 1.133(1.072-1.197) | 1.168(1.088-1.254) | 1.163(1.078-1.254) | 1.121(1.034-1.214) |
| lag12 | 0.793(0.698-0.902) | 0.795(0.699-0.903) | 0.802(0.709-0.908) | 0.811(0.72-0.913) | 0.82(0.733-0.918) | 0.831(0.75-0.922) | 0.844(0.77-0.925) | 0.858(0.794-0.927) | 0.875(0.823-0.929) | 0.896(0.857-0.937) | 0.925(0.898-0.952) | 0.963(0.95-0.977) | 1.015(1.009-1.021) | 1.078(1.044-1.113) | 1.137(1.074-1.204) | 1.174(1.091-1.264) | 1.169(1.082-1.264) | 1.127(1.038-1.224) |
| lag13 | 0.795(0.695-0.91) | 0.796(0.696-0.91) | 0.802(0.705-0.913) | 0.809(0.714-0.916) | 0.818(0.727-0.92) | 0.828(0.743-0.923) | 0.84(0.763-0.925) | 0.854(0.787-0.926) | 0.871(0.817-0.928) | 0.893(0.853-0.935) | 0.923(0.895-0.951) | 0.962(0.948-0.976) | 1.015(1.009-1.021) | 1.08(1.045-1.116) | 1.141(1.075-1.21) | 1.178(1.092-1.272) | 1.174(1.084-1.273) | 1.132(1.039-1.233) |
| lag14 | 0.798(0.693-0.918) | 0.798(0.694-0.918) | 0.802(0.701-0.919) | 0.808(0.709-0.921) | 0.816(0.721-0.922) | 0.825(0.736-0.924) | 0.836(0.756-0.925) | 0.85(0.781-0.926) | 0.868(0.812-0.927) | 0.891(0.849-0.935) | 0.921(0.892-0.951) | 0.961(0.947-0.976) | 1.016(1.009-1.022) | 1.081(1.044-1.119) | 1.143(1.075-1.216) | 1.182(1.092-1.279) | 1.178(1.084-1.281) | 1.137(1.04-1.242) |
| lag15 | 0.8(0.691-0.927) | 0.801(0.692-0.927) | 0.803(0.697-0.925) | 0.808(0.705-0.925) | 0.814(0.716-0.926) | 0.823(0.731-0.926) | 0.834(0.75-0.926) | 0.848(0.776-0.926) | 0.865(0.807-0.927) | 0.889(0.845-0.935) | 0.92(0.89-0.951) | 0.961(0.946-0.976) | 1.016(1.009-1.022) | 1.082(1.044-1.121) | 1.145(1.074-1.22) | 1.184(1.091-1.285) | 1.182(1.084-1.288) | 1.141(1.04-1.251) |
| lag16 | 0.803(0.689-0.936) | 0.803(0.69-0.935) | 0.804(0.694-0.932) | 0.808(0.701-0.93) | 0.813(0.711-0.93) | 0.821(0.725-0.929) | 0.832(0.745-0.928) | 0.845(0.771-0.927) | 0.863(0.803-0.928) | 0.887(0.842-0.935) | 0.919(0.888-0.951) | 0.961(0.945-0.976) | 1.016(1.009-1.023) | 1.082(1.043-1.123) | 1.146(1.073-1.224) | 1.186(1.09-1.29) | 1.184(1.084-1.294) | 1.145(1.041-1.259) |
| lag17 | 0.806(0.688-0.945) | 0.806(0.689-0.944) | 0.806(0.692-0.939) | 0.808(0.698-0.936) | 0.812(0.707-0.934) | 0.82(0.721-0.932) | 0.83(0.741-0.93) | 0.844(0.767-0.928) | 0.862(0.799-0.929) | 0.886(0.839-0.935) | 0.918(0.886-0.951) | 0.96(0.944-0.976) | 1.016(1.009-1.023) | 1.083(1.043-1.125) | 1.146(1.072-1.226) | 1.187(1.089-1.294) | 1.187(1.084-1.3) | 1.149(1.041-1.268) |
| lag18 | 0.81(0.687-0.954) | 0.809(0.687-0.952) | 0.808(0.69-0.946) | 0.809(0.695-0.941) | 0.812(0.703-0.938) | 0.819(0.717-0.935) | 0.829(0.736-0.933) | 0.842(0.763-0.93) | 0.86(0.796-0.93) | 0.885(0.836-0.936) | 0.917(0.884-0.952) | 0.96(0.944-0.977) | 1.016(1.009-1.023) | 1.083(1.042-1.126) | 1.147(1.071-1.229) | 1.189(1.088-1.298) | 1.19(1.084-1.306) | 1.153(1.043-1.276) |
| lag19 | 0.813(0.686-0.963) | 0.812(0.686-0.961) | 0.809(0.687-0.953) | 0.809(0.692-0.947) | 0.812(0.7-0.943) | 0.819(0.713-0.939) | 0.828(0.733-0.936) | 0.841(0.759-0.933) | 0.86(0.793-0.932) | 0.884(0.834-0.937) | 0.917(0.883-0.952) | 0.96(0.943-0.977) | 1.016(1.009-1.024) | 1.083(1.041-1.127) | 1.148(1.069-1.232) | 1.19(1.087-1.303) | 1.193(1.084-1.312) | 1.158(1.044-1.285) |
| lag20 | 0.816(0.685-0.973) | 0.815(0.684-0.971) | 0.812(0.685-0.961) | 0.811(0.689-0.954) | 0.813(0.696-0.949) | 0.819(0.71-0.944) | 0.828(0.729-0.94) | 0.841(0.756-0.936) | 0.859(0.79-0.934) | 0.884(0.831-0.939) | 0.916(0.881-0.953) | 0.96(0.942-0.978) | 1.016(1.008-1.024) | 1.084(1.04-1.129) | 1.148(1.068-1.235) | 1.192(1.086-1.308) | 1.196(1.084-1.32) | 1.164(1.045-1.297) |
| lag21 | 0.819(0.682-0.984) | 0.818(0.682-0.982) | 0.814(0.682-0.971) | 0.812(0.685-0.963) | 0.814(0.692-0.957) | 0.819(0.705-0.951) | 0.828(0.725-0.946) | 0.841(0.752-0.941) | 0.859(0.787-0.938) | 0.883(0.829-0.942) | 0.916(0.879-0.955) | 0.96(0.941-0.978) | 1.016(1.008-1.024) | 1.084(1.039-1.131) | 1.149(1.065-1.24) | 1.194(1.083-1.316) | 1.2(1.082-1.331) | 1.17(1.044-1.311) |

| Table S16 Risk of male HFMD with each 1 °C change in lag time from 13.8 °C to 30 °C | | | | | | | | | | | | | | | | | | |
| --- | --- | --- | --- | --- | --- | --- | --- | --- | --- | --- | --- | --- | --- | --- | --- | --- | --- | --- |
| Lag(days) | Temperature(℃), RR(95%CI) | | | | | | | | | | | | | | | | | |
|  | 13.8 | 14 | 15 | 16 | 17 | 18 | 19 | 20 | 21 | 22 | 23 | 24 | 25 | 26 | 27 | 28 | 29 | 30 |
| lag0 | 0.86(0.803~0.92) | 0.862(0.807~0.921) | 0.877(0.825~0.931) | 0.89(0.841~0.942) | 0.903(0.857~0.951) | 0.915(0.874~0.959) | 0.926(0.89~0.964) | 0.937(0.906~0.969) | 0.948(0.922~0.975) | 0.96(0.937~0.983) | 0.973(0.956~0.991) | 0.989(0.981~0.997) | 1.004(1.001~1.007) | 1.017(1.005~1.028) | 1.025(1.007~1.043) | 1.026(1~1.052) | 1.015(0.985~1.047) | 0.993(0.961~1.027) |
| lag1 | 0.923(0.862~0.989) | 0.924(0.864~0.989) | 0.929(0.873~0.989) | 0.934(0.881~0.989) | 0.937(0.889~0.989) | 0.94(0.896~0.986) | 0.941(0.903~0.981) | 0.943(0.911~0.976) | 0.946(0.918~0.974) | 0.952(0.928~0.977) | 0.964(0.946~0.983) | 0.982(0.974~0.991) | 1.007(1.004~1.01) | 1.037(1.024~1.049) | 1.07(1.05~1.09) | 1.095(1.066~1.124) | 1.091(1.057~1.127) | 1.053(1.017~1.09) |
| lag2 | 1.003(0.938~1.074) | 1.002(0.937~1.071) | 0.993(0.933~1.057) | 0.984(0.929~1.043) | 0.975(0.924~1.029) | 0.965(0.92~1.013) | 0.955(0.916~0.996) | 0.946(0.914~0.98) | 0.941(0.913~0.971) | 0.943(0.917~0.968) | 0.953(0.934~0.973) | 0.975(0.966~0.984) | 1.011(1.008~1.014) | 1.062(1.049~1.076) | 1.131(1.11~1.153) | 1.193(1.161~1.225) | 1.203(1.166~1.242) | 1.144(1.106~1.182) |
| lag3 | 0.997(0.926~1.073) | 0.994(0.924~1.069) | 0.98(0.915~1.048) | 0.966(0.906~1.029) | 0.953(0.898~1.011) | 0.94(0.891~0.991) | 0.928(0.886~0.971) | 0.918(0.883~0.953) | 0.913(0.883~0.944) | 0.917(0.89~0.944) | 0.933(0.912~0.954) | 0.964(0.954~0.974) | 1.016(1.012~1.019) | 1.094(1.079~1.109) | 1.203(1.179~1.229) | 1.307(1.271~1.345) | 1.331(1.287~1.375) | 1.24(1.198~1.284) |
| lag4 | 0.932(0.86~1.01) | 0.929(0.859~1.006) | 0.916(0.851~0.986) | 0.905(0.844~0.969) | 0.894(0.839~0.953) | 0.884(0.835~0.936) | 0.876(0.834~0.919) | 0.871(0.836~0.907) | 0.872(0.841~0.903) | 0.883(0.855~0.911) | 0.908(0.886~0.93) | 0.951(0.941~0.962) | 1.021(1.017~1.025) | 1.128(1.112~1.145) | 1.282(1.255~1.31) | 1.433(1.391~1.476) | 1.468(1.419~1.519) | 1.337(1.29~1.385) |
| lag5 | 0.85(0.778~0.928) | 0.848(0.777~0.925) | 0.839(0.774~0.909) | 0.831(0.771~0.897) | 0.826(0.77~0.885) | 0.821(0.772~0.874) | 0.819(0.777~0.863) | 0.82(0.785~0.857) | 0.828(0.797~0.86) | 0.847(0.819~0.877) | 0.882(0.859~0.905) | 0.938(0.927~0.95) | 1.027(1.023~1.031) | 1.163(1.144~1.181) | 1.363(1.332~1.394) | 1.564(1.516~1.613) | 1.61(1.554~1.669) | 1.432(1.379~1.486) |
| lag6 | 0.774(0.702~0.854) | 0.773(0.702~0.851) | 0.768(0.703~0.84) | 0.765(0.704~0.832) | 0.764(0.707~0.825) | 0.764(0.714~0.819) | 0.767(0.724~0.813) | 0.774(0.738~0.813) | 0.789(0.757~0.823) | 0.816(0.786~0.846) | 0.859(0.835~0.884) | 0.927(0.915~0.939) | 1.032(1.028~1.036) | 1.196(1.175~1.216) | 1.442(1.407~1.478) | 1.695(1.639~1.752) | 1.752(1.686~1.821) | 1.522(1.463~1.584) |
| lag7 | 0.715(0.642~0.796) | 0.714(0.643~0.794) | 0.713(0.646~0.786) | 0.713(0.65~0.781) | 0.715(0.657~0.778) | 0.719(0.667~0.775) | 0.726(0.682~0.774) | 0.738(0.7~0.777) | 0.757(0.724~0.792) | 0.789(0.758~0.822) | 0.839(0.814~0.866) | 0.917(0.904~0.93) | 1.037(1.032~1.041) | 1.225(1.203~1.248) | 1.517(1.477~1.557) | 1.822(1.758~1.889) | 1.89(1.814~1.969) | 1.607(1.54~1.677) |
| lag8 | 0.671(0.598~0.754) | 0.671(0.599~0.752) | 0.672(0.604~0.747) | 0.674(0.611~0.744) | 0.679(0.62~0.743) | 0.686(0.632~0.743) | 0.695(0.649~0.744) | 0.71(0.671~0.751) | 0.732(0.698~0.769) | 0.769(0.736~0.803) | 0.824(0.797~0.852) | 0.908(0.895~0.922) | 1.04(1.035~1.046) | 1.252(1.228~1.277) | 1.586(1.542~1.631) | 1.943(1.871~2.019) | 2.022(1.937~2.111) | 1.685(1.611~1.762) |
| lag9 | 0.64(0.565~0.724) | 0.64(0.567~0.723) | 0.642(0.573~0.719) | 0.646(0.581~0.718) | 0.652(0.592~0.718) | 0.661(0.606~0.72) | 0.672(0.625~0.723) | 0.688(0.648~0.731) | 0.714(0.678~0.751) | 0.752(0.719~0.788) | 0.811(0.783~0.84) | 0.901(0.887~0.916) | 1.044(1.038~1.049) | 1.276(1.249~1.303) | 1.65(1.602~1.699) | 2.059(1.978~2.142) | 2.148(2.053~2.247) | 1.757(1.677~1.84) |
| lag10 | 0.616(0.541~0.703) | 0.617(0.542~0.702) | 0.62(0.55~0.699) | 0.625(0.559~0.699) | 0.632(0.571~0.7) | 0.642(0.586~0.703) | 0.655(0.606~0.707) | 0.672(0.631~0.716) | 0.699(0.662~0.738) | 0.74(0.705~0.777) | 0.802(0.773~0.832) | 0.896(0.881~0.911) | 1.047(1.041~1.052) | 1.296(1.268~1.325) | 1.708(1.657~1.761) | 2.168(2.08~2.259) | 2.267(2.164~2.376) | 1.823(1.738~1.913) |
| lag11 | 0.599(0.522~0.688) | 0.6(0.524~0.687) | 0.604(0.532~0.685) | 0.61(0.542~0.686) | 0.618(0.555~0.688) | 0.628(0.571~0.691) | 0.642(0.592~0.696) | 0.661(0.618~0.706) | 0.689(0.65~0.729) | 0.731(0.695~0.769) | 0.794(0.764~0.826) | 0.891(0.876~0.907) | 1.049(1.043~1.055) | 1.315(1.285~1.345) | 1.762(1.707~1.82) | 2.271(2.175~2.371) | 2.381(2.269~2.5) | 1.885(1.793~1.981) |
| lag12 | 0.586(0.507~0.678) | 0.587(0.509~0.677) | 0.592(0.518~0.675) | 0.598(0.529~0.677) | 0.607(0.542~0.68) | 0.618(0.559~0.684) | 0.633(0.581~0.689) | 0.652(0.608~0.7) | 0.681(0.641~0.724) | 0.724(0.687~0.764) | 0.789(0.757~0.822) | 0.888(0.872~0.905) | 1.051(1.044~1.057) | 1.33(1.299~1.363) | 1.812(1.752~1.874) | 2.37(2.266~2.479) | 2.491(2.368~2.62) | 1.943(1.845~2.046) |
| lag13 | 0.576(0.495~0.671) | 0.577(0.497~0.67) | 0.582(0.507~0.669) | 0.589(0.518~0.67) | 0.599(0.532~0.674) | 0.611(0.55~0.678) | 0.626(0.573~0.684) | 0.646(0.6~0.696) | 0.676(0.634~0.72) | 0.72(0.68~0.761) | 0.785(0.752~0.819) | 0.885(0.868~0.903) | 1.052(1.046~1.059) | 1.344(1.311~1.379) | 1.858(1.794~1.924) | 2.464(2.352~2.583) | 2.597(2.464~2.737) | 1.998(1.893~2.108) |
| lag14 | 0.568(0.484~0.665) | 0.569(0.486~0.665) | 0.574(0.497~0.664) | 0.582(0.508~0.666) | 0.592(0.523~0.67) | 0.605(0.542~0.675) | 0.62(0.565~0.681) | 0.642(0.594~0.693) | 0.672(0.629~0.719) | 0.717(0.676~0.76) | 0.782(0.748~0.819) | 0.884(0.866~0.902) | 1.054(1.047~1.061) | 1.357(1.321~1.393) | 1.901(1.833~1.972) | 2.556(2.434~2.684) | 2.701(2.557~2.853) | 2.05(1.939~2.169) |
| lag15 | 0.56(0.474~0.66) | 0.561(0.476~0.66) | 0.567(0.487~0.659) | 0.575(0.499~0.662) | 0.586(0.515~0.666) | 0.599(0.534~0.672) | 0.616(0.559~0.679) | 0.638(0.589~0.692) | 0.67(0.625~0.718) | 0.715(0.672~0.761) | 0.781(0.745~0.819) | 0.882(0.864~0.901) | 1.055(1.047~1.062) | 1.368(1.33~1.406) | 1.942(1.869~2.017) | 2.645(2.514~2.783) | 2.803(2.648~2.967) | 2.102(1.983~2.228) |
| lag16 | 0.551(0.464~0.655) | 0.552(0.466~0.654) | 0.559(0.478~0.654) | 0.568(0.491~0.657) | 0.579(0.507~0.662) | 0.594(0.528~0.669) | 0.612(0.553~0.677) | 0.636(0.584~0.692) | 0.668(0.621~0.719) | 0.715(0.67~0.762) | 0.781(0.743~0.82) | 0.881(0.862~0.901) | 1.055(1.048~1.063) | 1.377(1.338~1.417) | 1.98(1.904~2.06) | 2.733(2.593~2.881) | 2.905(2.739~3.082) | 2.153(2.027~2.287) |
| lag17 | 0.542(0.454~0.647) | 0.543(0.456~0.647) | 0.55(0.468~0.647) | 0.56(0.482~0.651) | 0.573(0.499~0.657) | 0.589(0.521~0.665) | 0.608(0.548~0.675) | 0.633(0.58~0.691) | 0.668(0.619~0.72) | 0.715(0.668~0.765) | 0.781(0.742~0.822) | 0.881(0.861~0.902) | 1.056(1.048~1.064) | 1.386(1.345~1.427) | 2.018(1.937~2.101) | 2.821(2.672~2.979) | 3.009(2.831~3.198) | 2.205(2.071~2.347) |
| lag18 | 0.531(0.443~0.638) | 0.533(0.445~0.638) | 0.541(0.458~0.638) | 0.551(0.472~0.643) | 0.565(0.49~0.651) | 0.582(0.513~0.661) | 0.604(0.542~0.673) | 0.631(0.576~0.691) | 0.667(0.616~0.722) | 0.716(0.667~0.768) | 0.782(0.741~0.825) | 0.881(0.86~0.903) | 1.057(1.048~1.065) | 1.393(1.351~1.437) | 2.053(1.969~2.141) | 2.909(2.751~3.077) | 3.114(2.925~3.316) | 2.257(2.116~2.408) |
| lag19 | 0.519(0.43~0.627) | 0.521(0.433~0.626) | 0.529(0.446~0.628) | 0.541(0.461~0.634) | 0.556(0.48~0.643) | 0.575(0.505~0.655) | 0.599(0.536~0.669) | 0.628(0.572~0.69) | 0.667(0.614~0.724) | 0.717(0.667~0.771) | 0.784(0.741~0.828) | 0.882(0.86~0.904) | 1.057(1.048~1.065) | 1.4(1.356~1.445) | 2.088(2~2.181) | 2.998(2.831~3.176) | 3.222(3.021~3.437) | 2.311(2.162~2.471) |
| lag20 | 0.505(0.416~0.613) | 0.507(0.419~0.613) | 0.516(0.433~0.616) | 0.529(0.449~0.623) | 0.546(0.469~0.634) | 0.567(0.495~0.648) | 0.593(0.528~0.665) | 0.626(0.567~0.69) | 0.667(0.612~0.726) | 0.719(0.666~0.776) | 0.786(0.742~0.832) | 0.882(0.86~0.906) | 1.057(1.048~1.066) | 1.406(1.361~1.454) | 2.123(2.029~2.22) | 3.089(2.91~3.279) | 3.334(3.118~3.565) | 2.367(2.209~2.538) |
| lag21 | 0.489(0.4~0.598) | 0.491(0.403~0.598) | 0.502(0.418~0.602) | 0.516(0.435~0.611) | 0.534(0.457~0.624) | 0.557(0.485~0.64) | 0.586(0.52~0.661) | 0.622(0.562~0.689) | 0.667(0.609~0.729) | 0.721(0.666~0.781) | 0.788(0.742~0.838) | 0.883(0.86~0.908) | 1.057(1.048~1.066) | 1.412(1.364~1.462) | 2.156(2.057~2.26) | 3.181(2.989~3.386) | 3.45(3.216~3.701) | 2.426(2.255~2.61) |

| Table S17 Risk of female HFMD with each 1 °C change in lag time from 13.8 °C to 30 °C | | | | | | | | | | | | | | | | | | |
| --- | --- | --- | --- | --- | --- | --- | --- | --- | --- | --- | --- | --- | --- | --- | --- | --- | --- | --- |
| Lag(days) | Temperature(℃), RR(95%CI) | | | | | | | | | | | | | | | | | |
|  | 13.8 | 14 | 15 | 16 | 17 | 18 | 19 | 20 | 21 | 22 | 23 | 24 | 25 | 26 | 27 | 28 | 29 | 30 |
| lag0 | 0.853(0.791~0.919) | 0.855(0.794~0.921) | 0.868(0.811~0.929) | 0.88(0.827~0.937) | 0.893(0.842~0.946) | 0.905(0.859~0.953) | 0.917(0.877~0.959) | 0.93(0.896~0.965) | 0.943(0.914~0.973) | 0.957(0.931~0.983) | 0.972(0.953~0.992) | 0.988(0.98~0.997) | 1.004(1.001~1.007) | 1.015(1.002~1.028) | 1.021(1~1.041) | 1.016(0.988~1.045) | 0.998(0.965~1.033) | 0.967(0.932~1.004) |
| lag1 | 0.912(0.845~0.985) | 0.913(0.847~0.985) | 0.918(0.857~0.984) | 0.923(0.866~0.985) | 0.929(0.875~0.986) | 0.934(0.885~0.986) | 0.94(0.898~0.984) | 0.946(0.91~0.983) | 0.954(0.922~0.986) | 0.962(0.935~0.99) | 0.974(0.953~0.995) | 0.988(0.978~0.997) | 1.005(1.002~1.008) | 1.026(1.013~1.041) | 1.052(1.03~1.074) | 1.069(1.037~1.101) | 1.058(1.02~1.096) | 1.012(0.974~1.052) |
| lag2 | 0.993(0.921~1.072) | 0.992(0.921~1.069) | 0.985(0.919~1.056) | 0.979(0.917~1.044) | 0.973(0.916~1.034) | 0.969(0.918~1.023) | 0.965(0.921~1.011) | 0.963(0.926~1.002) | 0.963(0.931~0.997) | 0.966(0.937~0.996) | 0.973(0.951~0.995) | 0.985(0.975~0.995) | 1.007(1.003~1.01) | 1.044(1.029~1.059) | 1.098(1.075~1.123) | 1.149(1.114~1.184) | 1.153(1.112~1.194) | 1.092(1.052~1.134) |
| lag3 | 0.993(0.914~1.079) | 0.99(0.913~1.074) | 0.978(0.907~1.055) | 0.967(0.901~1.039) | 0.958(0.897~1.023) | 0.95(0.896~1.008) | 0.944(0.897~0.993) | 0.94(0.901~0.982) | 0.94(0.906~0.976) | 0.945(0.914~0.977) | 0.956(0.932~0.98) | 0.976(0.964~0.987) | 1.011(1.007~1.015) | 1.07(1.054~1.087) | 1.161(1.134~1.188) | 1.248(1.208~1.288) | 1.264(1.217~1.312) | 1.178(1.133~1.225) |
| lag4 | 0.937(0.857~1.025) | 0.934(0.855~1.02) | 0.923(0.85~1.002) | 0.913(0.845~0.986) | 0.905(0.843~0.972) | 0.899(0.843~0.958) | 0.895(0.847~0.945) | 0.894(0.854~0.936) | 0.898(0.863~0.935) | 0.909(0.878~0.942) | 0.929(0.904~0.955) | 0.962(0.951~0.974) | 1.017(1.013~1.021) | 1.103(1.085~1.122) | 1.233(1.203~1.263) | 1.359(1.315~1.405) | 1.385(1.333~1.44) | 1.266(1.216~1.318) |
| lag5 | 0.862(0.781~0.951) | 0.86(0.781~0.947) | 0.852(0.778~0.932) | 0.845(0.777~0.92) | 0.841(0.778~0.909) | 0.838(0.782~0.898) | 0.838(0.79~0.889) | 0.842(0.801~0.884) | 0.851(0.816~0.889) | 0.87(0.837~0.904) | 0.9(0.874~0.927) | 0.948(0.936~0.961) | 1.023(1.018~1.027) | 1.138(1.118~1.158) | 1.309(1.276~1.343) | 1.478(1.428~1.531) | 1.513(1.453~1.575) | 1.353(1.297~1.411) |
| lag6 | 0.792(0.71~0.883) | 0.791(0.711~0.88) | 0.786(0.711~0.868) | 0.783(0.713~0.859) | 0.782(0.717~0.852) | 0.783(0.725~0.845) | 0.786(0.737~0.839) | 0.794(0.752~0.838) | 0.809(0.772~0.848) | 0.834(0.8~0.87) | 0.874(0.846~0.902) | 0.935(0.921~0.949) | 1.028(1.023~1.033) | 1.172(1.15~1.195) | 1.385(1.348~1.424) | 1.599(1.54~1.66) | 1.641(1.572~1.713) | 1.437(1.374~1.503) |
| lag7 | 0.736(0.653~0.829) | 0.735(0.654~0.827) | 0.733(0.658~0.818) | 0.733(0.662~0.812) | 0.735(0.669~0.807) | 0.739(0.679~0.803) | 0.745(0.694~0.8) | 0.756(0.712~0.802) | 0.774(0.736~0.815) | 0.804(0.769~0.842) | 0.852(0.822~0.882) | 0.923(0.909~0.938) | 1.033(1.028~1.039) | 1.203(1.178~1.228) | 1.458(1.415~1.501) | 1.717(1.649~1.787) | 1.767(1.688~1.851) | 1.517(1.446~1.591) |
| lag8 | 0.695(0.611~0.79) | 0.695(0.612~0.788) | 0.695(0.618~0.782) | 0.697(0.624~0.778) | 0.7(0.633~0.775) | 0.706(0.645~0.772) | 0.714(0.662~0.771) | 0.727(0.682~0.774) | 0.748(0.708~0.79) | 0.781(0.744~0.82) | 0.834(0.803~0.866) | 0.914(0.899~0.93) | 1.037(1.032~1.043) | 1.23(1.203~1.258) | 1.524(1.477~1.573) | 1.829(1.753~1.909) | 1.889(1.799~1.983) | 1.593(1.515~1.675) |
| lag9 | 0.665(0.58~0.762) | 0.665(0.581~0.761) | 0.667(0.588~0.756) | 0.67(0.596~0.753) | 0.675(0.606~0.752) | 0.682(0.62~0.751) | 0.691(0.638~0.75) | 0.705(0.659~0.754) | 0.728(0.687~0.771) | 0.764(0.726~0.804) | 0.82(0.788~0.853) | 0.906(0.89~0.923) | 1.041(1.035~1.047) | 1.254(1.225~1.284) | 1.586(1.535~1.64) | 1.937(1.852~2.025) | 2.006(1.907~2.11) | 1.664(1.579~1.753) |
| lag10 | 0.643(0.556~0.743) | 0.643(0.558~0.742) | 0.646(0.566~0.738) | 0.651(0.575~0.737) | 0.657(0.586~0.736) | 0.665(0.601~0.736) | 0.675(0.62~0.735) | 0.69(0.642~0.74) | 0.713(0.671~0.758) | 0.751(0.711~0.793) | 0.809(0.776~0.844) | 0.9(0.884~0.918) | 1.044(1.038~1.051) | 1.275(1.244~1.307) | 1.643(1.587~1.701) | 2.039(1.947~2.136) | 2.119(2.01~2.233) | 1.73(1.639~1.826) |
| lag11 | 0.627(0.539~0.731) | 0.628(0.54~0.73) | 0.632(0.55~0.727) | 0.637(0.559~0.726) | 0.644(0.572~0.726) | 0.653(0.587~0.726) | 0.663(0.606~0.726) | 0.679(0.63~0.731) | 0.703(0.659~0.749) | 0.741(0.7~0.785) | 0.802(0.767~0.837) | 0.896(0.878~0.914) | 1.046(1.04~1.053) | 1.294(1.261~1.327) | 1.696(1.636~1.758) | 2.137(2.036~2.243) | 2.227(2.108~2.351) | 1.793(1.695~1.896) |
| lag12 | 0.616(0.525~0.724) | 0.617(0.527~0.723) | 0.622(0.537~0.721) | 0.628(0.548~0.72) | 0.636(0.561~0.721) | 0.645(0.577~0.721) | 0.656(0.596~0.721) | 0.671(0.62~0.726) | 0.695(0.65~0.744) | 0.734(0.692~0.78) | 0.796(0.76~0.833) | 0.892(0.874~0.911) | 1.048(1.041~1.056) | 1.31(1.275~1.346) | 1.744(1.68~1.811) | 2.23(2.121~2.345) | 2.33(2.202~2.466) | 1.852(1.747~1.962) |
| lag13 | 0.609(0.514~0.72) | 0.61(0.517~0.72) | 0.615(0.527~0.718) | 0.622(0.539~0.718) | 0.63(0.552~0.718) | 0.639(0.569~0.719) | 0.65(0.589~0.718) | 0.666(0.613~0.723) | 0.69(0.643~0.742) | 0.73(0.685~0.777) | 0.791(0.754~0.831) | 0.889(0.87~0.909) | 1.05(1.043~1.058) | 1.324(1.287~1.362) | 1.789(1.72~1.86) | 2.319(2.201~2.445) | 2.431(2.292~2.579) | 1.907(1.795~2.025) |
| lag14 | 0.603(0.506~0.719) | 0.604(0.508~0.719) | 0.61(0.52~0.717) | 0.618(0.532~0.717) | 0.626(0.545~0.718) | 0.636(0.563~0.718) | 0.647(0.583~0.718) | 0.663(0.608~0.723) | 0.687(0.638~0.741) | 0.727(0.68~0.777) | 0.789(0.75~0.83) | 0.887(0.867~0.908) | 1.052(1.044~1.059) | 1.337(1.298~1.377) | 1.831(1.758~1.907) | 2.406(2.277~2.541) | 2.528(2.378~2.689) | 1.959(1.84~2.086) |
| lag15 | 0.599(0.499~0.719) | 0.6(0.501~0.719) | 0.607(0.513~0.717) | 0.614(0.526~0.718) | 0.623(0.54~0.719) | 0.633(0.558~0.719) | 0.645(0.579~0.719) | 0.661(0.604~0.724) | 0.686(0.634~0.742) | 0.725(0.676~0.777) | 0.787(0.746~0.83) | 0.885(0.864~0.907) | 1.053(1.044~1.061) | 1.348(1.307~1.39) | 1.871(1.793~1.952) | 2.489(2.352~2.635) | 2.623(2.461~2.797) | 2.008(1.881~2.144) |
| lag16 | 0.595(0.492~0.72) | 0.596(0.495~0.719) | 0.604(0.507~0.718) | 0.612(0.52~0.719) | 0.621(0.535~0.72) | 0.632(0.553~0.721) | 0.644(0.575~0.72) | 0.66(0.601~0.725) | 0.685(0.631~0.744) | 0.724(0.674~0.779) | 0.786(0.743~0.83) | 0.884(0.862~0.907) | 1.054(1.045~1.062) | 1.358(1.315~1.403) | 1.908(1.826~1.995) | 2.572(2.424~2.728) | 2.717(2.543~2.903) | 2.056(1.921~2.2) |
| lag17 | 0.591(0.486~0.72) | 0.593(0.489~0.719) | 0.6(0.502~0.718) | 0.609(0.515~0.719) | 0.619(0.531~0.721) | 0.63(0.55~0.722) | 0.643(0.572~0.722) | 0.659(0.598~0.727) | 0.685(0.629~0.746) | 0.724(0.671~0.781) | 0.785(0.741~0.832) | 0.883(0.861~0.907) | 1.054(1.046~1.063) | 1.368(1.323~1.414) | 1.945(1.858~2.036) | 2.653(2.496~2.819) | 2.81(2.624~3.008) | 2.101(1.959~2.253) |
| lag18 | 0.587(0.479~0.719) | 0.589(0.482~0.718) | 0.597(0.496~0.717) | 0.606(0.51~0.719) | 0.616(0.526~0.721) | 0.628(0.545~0.722) | 0.641(0.569~0.723) | 0.659(0.595~0.729) | 0.685(0.626~0.748) | 0.724(0.67~0.784) | 0.785(0.74~0.834) | 0.883(0.859~0.907) | 1.055(1.046~1.065) | 1.376(1.33~1.425) | 1.98(1.889~2.075) | 2.733(2.567~2.909) | 2.902(2.705~3.113) | 2.144(1.995~2.305) |
| lag19 | 0.582(0.473~0.717) | 0.583(0.475~0.716) | 0.592(0.49~0.715) | 0.601(0.504~0.717) | 0.612(0.521~0.72) | 0.625(0.541~0.722) | 0.639(0.565~0.724) | 0.658(0.592~0.731) | 0.685(0.624~0.751) | 0.725(0.668~0.787) | 0.785(0.738~0.836) | 0.882(0.858~0.907) | 1.056(1.046~1.066) | 1.385(1.336~1.435) | 2.015(1.919~2.115) | 2.813(2.638~3) | 2.993(2.784~3.218) | 2.186(2.029~2.356) |
| lag20 | 0.576(0.465~0.714) | 0.577(0.468~0.713) | 0.586(0.482~0.712) | 0.596(0.497~0.715) | 0.608(0.515~0.718) | 0.621(0.535~0.721) | 0.637(0.56~0.724) | 0.657(0.589~0.732) | 0.685(0.622~0.754) | 0.725(0.666~0.79) | 0.786(0.737~0.839) | 0.882(0.857~0.908) | 1.056(1.046~1.066) | 1.392(1.342~1.445) | 2.049(1.948~2.154) | 2.894(2.707~3.093) | 3.085(2.862~3.326) | 2.227(2.06~2.407) |
| lag21 | 0.568(0.455~0.71) | 0.57(0.458~0.709) | 0.579(0.473~0.709) | 0.59(0.489~0.712) | 0.602(0.507~0.716) | 0.617(0.529~0.72) | 0.634(0.555~0.724) | 0.655(0.585~0.734) | 0.684(0.619~0.757) | 0.726(0.664~0.794) | 0.786(0.735~0.842) | 0.882(0.855~0.909) | 1.057(1.046~1.067) | 1.4(1.347~1.455) | 2.082(1.976~2.194) | 2.974(2.774~3.189) | 3.178(2.937~3.438) | 2.266(2.088~2.46) |

| Table S18 Risk of 0y DV with each 1 °C change in lag time from 13.8 °C to 30 °C | | | | | | | | | | | | | | | | | | |
| --- | --- | --- | --- | --- | --- | --- | --- | --- | --- | --- | --- | --- | --- | --- | --- | --- | --- | --- |
| Lag(days) | Temperature(℃) (0y), RR(95%CI) | | | | | | | | | | | | | | | | | |
|  | 13.8 | 14 | 15 | 16 | 17 | 18 | 19 | 20 | 21 | 22 | 23 | 24 | 25 | 26 | 27 | 28 | 29 | 30 |
| lag0 | 0.892(0.807-0.987) | 0.898(0.812-0.992) | 0.922(0.838-1.014) | 0.943(0.862-1.033) | 0.962(0.883-1.047) | 0.976(0.903-1.055) | 0.987(0.92-1.058) | 0.992(0.935-1.053) | 0.994(0.948-1.043) | 0.994(0.958-1.031) | 0.994(0.969-1.02) | 0.996(0.984-1.008) | 1.002(0.997-1.007) | 1.012(0.984-1.041) | 1.022(0.971-1.075) | 1.027(0.959-1.1) | 1.023(0.945-1.109) | 1.012(0.922-1.11) |
| lag1 | 0.804(0.736-0.879) | 0.809(0.741-0.883) | 0.833(0.765-0.907) | 0.855(0.788-0.928) | 0.876(0.811-0.947) | 0.896(0.834-0.963) | 0.914(0.857-0.974) | 0.929(0.879-0.982) | 0.943(0.901-0.986) | 0.956(0.923-0.99) | 0.97(0.946-0.994) | 0.986(0.974-0.998) | 1.006(1-1.011) | 1.029(1-1.059) | 1.053(1-1.109) | 1.073(1.002-1.149) | 1.085(1.003-1.173) | 1.089(0.996-1.19) |
| lag2 | 0.795(0.732-0.863) | 0.8(0.737-0.868) | 0.825(0.762-0.892) | 0.849(0.787-0.916) | 0.872(0.812-0.937) | 0.894(0.837-0.955) | 0.915(0.862-0.97) | 0.933(0.887-0.981) | 0.949(0.911-0.989) | 0.964(0.934-0.996) | 0.978(0.956-1.001) | 0.991(0.979-1.002) | 1.003(0.998-1.008) | 1.015(0.988-1.044) | 1.028(0.978-1.08) | 1.04(0.974-1.11) | 1.052(0.978-1.131) | 1.064(0.979-1.155) |
| lag3 | 0.815(0.746-0.89) | 0.82(0.75-0.895) | 0.843(0.775-0.919) | 0.866(0.798-0.94) | 0.888(0.821-0.959) | 0.907(0.845-0.975) | 0.925(0.868-0.987) | 0.941(0.89-0.994) | 0.954(0.912-0.998) | 0.966(0.933-1.001) | 0.978(0.954-1.003) | 0.99(0.978-1.003) | 1.004(0.998-1.009) | 1.019(0.989-1.05) | 1.034(0.98-1.091) | 1.047(0.976-1.123) | 1.056(0.978-1.141) | 1.062(0.974-1.158) |
| lag4 | 0.845(0.771-0.926) | 0.849(0.775-0.93) | 0.87(0.796-0.951) | 0.89(0.817-0.969) | 0.907(0.837-0.984) | 0.923(0.856-0.995) | 0.937(0.876-1.002) | 0.947(0.895-1.004) | 0.956(0.912-1.003) | 0.965(0.93-1.001) | 0.975(0.949-1.001) | 0.988(0.974-1.001) | 1.005(0.999-1.011) | 1.027(0.995-1.06) | 1.049(0.991-1.111) | 1.067(0.99-1.149) | 1.074(0.99-1.164) | 1.072(0.98-1.171) |
| lag5 | 0.876(0.793-0.969) | 0.88(0.797-0.972) | 0.897(0.814-0.988) | 0.913(0.831-1.002) | 0.927(0.848-1.012) | 0.938(0.865-1.019) | 0.948(0.881-1.02) | 0.955(0.897-1.017) | 0.961(0.913-1.012) | 0.967(0.929-1.007) | 0.975(0.947-1.003) | 0.987(0.973-1.002) | 1.006(0.999-1.012) | 1.03(0.995-1.066) | 1.055(0.992-1.123) | 1.074(0.991-1.164) | 1.081(0.99-1.179) | 1.076(0.978-1.184) |
| lag6 | 0.908(0.816-1.01) | 0.91(0.819-1.013) | 0.924(0.833-1.024) | 0.935(0.847-1.033) | 0.946(0.861-1.039) | 0.954(0.875-1.041) | 0.961(0.889-1.039) | 0.966(0.903-1.032) | 0.969(0.918-1.023) | 0.973(0.932-1.015) | 0.979(0.949-1.009) | 0.989(0.974-1.004) | 1.005(0.998-1.012) | 1.027(0.99-1.065) | 1.049(0.984-1.12) | 1.068(0.981-1.161) | 1.076(0.982-1.178) | 1.074(0.972-1.187) |
| lag7 | 0.941(0.84-1.054) | 0.943(0.842-1.056) | 0.952(0.854-1.063) | 0.961(0.864-1.067) | 0.967(0.876-1.069) | 0.973(0.887-1.067) | 0.977(0.9-1.06) | 0.979(0.912-1.05) | 0.98(0.925-1.037) | 0.981(0.939-1.026) | 0.985(0.954-1.016) | 0.992(0.976-1.008) | 1.004(0.997-1.011) | 1.021(0.983-1.06) | 1.04(0.972-1.112) | 1.056(0.968-1.152) | 1.066(0.971-1.171) | 1.071(0.965-1.187) |
| lag8 | 0.979(0.867-1.105) | 0.98(0.869-1.106) | 0.986(0.877-1.108) | 0.991(0.885-1.108) | 0.994(0.894-1.105) | 0.996(0.903-1.098) | 0.996(0.913-1.087) | 0.995(0.924-1.072) | 0.993(0.935-1.055) | 0.991(0.946-1.039) | 0.991(0.959-1.025) | 0.995(0.978-1.012) | 1.003(0.995-1.01) | 1.015(0.976-1.056) | 1.031(0.96-1.106) | 1.046(0.955-1.146) | 1.059(0.96-1.169) | 1.07(0.959-1.193) |
| lag9 | 1.023(0.899-1.164) | 1.024(0.9-1.164) | 1.026(0.906-1.162) | 1.027(0.911-1.158) | 1.027(0.917-1.15) | 1.025(0.923-1.137) | 1.021(0.93-1.12) | 1.015(0.938-1.099) | 1.008(0.946-1.075) | 1.002(0.954-1.053) | 0.998(0.963-1.034) | 0.997(0.98-1.015) | 1.002(0.994-1.01) | 1.012(0.971-1.055) | 1.026(0.952-1.106) | 1.042(0.946-1.148) | 1.058(0.954-1.174) | 1.074(0.957-1.205) |
| lag10 | 1.074(0.937-1.232) | 1.074(0.938-1.231) | 1.074(0.941-1.225) | 1.071(0.944-1.216) | 1.067(0.947-1.202) | 1.06(0.95-1.183) | 1.05(0.953-1.158) | 1.039(0.956-1.129) | 1.025(0.958-1.097) | 1.013(0.961-1.067) | 1.003(0.967-1.041) | 0.999(0.98-1.018) | 1.001(0.993-1.01) | 1.012(0.969-1.057) | 1.028(0.95-1.112) | 1.047(0.946-1.158) | 1.066(0.956-1.188) | 1.084(0.961-1.223) |
| lag11 | 1.133(0.982-1.307) | 1.133(0.982-1.306) | 1.129(0.984-1.296) | 1.123(0.984-1.281) | 1.113(0.983-1.261) | 1.101(0.981-1.234) | 1.084(0.979-1.201) | 1.065(0.976-1.161) | 1.043(0.972-1.119) | 1.023(0.969-1.08) | 1.008(0.969-1.047) | 1(0.98-1.019) | 1.002(0.993-1.01) | 1.015(0.97-1.062) | 1.035(0.955-1.123) | 1.059(0.953-1.176) | 1.08(0.965-1.209) | 1.1(0.971-1.246) |
| lag12 | 1.199(1.033-1.392) | 1.198(1.033-1.39) | 1.192(1.032-1.375) | 1.181(1.029-1.355) | 1.166(1.024-1.327) | 1.146(1.017-1.292) | 1.122(1.009-1.248) | 1.093(0.999-1.196) | 1.062(0.987-1.142) | 1.033(0.976-1.093) | 1.011(0.971-1.052) | 0.999(0.979-1.02) | 1.002(0.994-1.011) | 1.021(0.974-1.07) | 1.048(0.963-1.14) | 1.077(0.967-1.201) | 1.101(0.98-1.237) | 1.119(0.983-1.273) |
| lag13 | 1.272(1.089-1.486) | 1.27(1.089-1.483) | 1.261(1.086-1.464) | 1.245(1.079-1.437) | 1.224(1.069-1.4) | 1.196(1.056-1.354) | 1.162(1.041-1.298) | 1.123(1.022-1.233) | 1.081(1.001-1.166) | 1.042(0.983-1.105) | 1.013(0.972-1.056) | 0.999(0.978-1.02) | 1.003(0.994-1.013) | 1.028(0.979-1.08) | 1.065(0.976-1.162) | 1.101(0.984-1.232) | 1.127(0.999-1.271) | 1.14(0.998-1.303) |
| lag14 | 1.351(1.149-1.589) | 1.349(1.149-1.585) | 1.335(1.143-1.56) | 1.314(1.132-1.526) | 1.285(1.117-1.479) | 1.249(1.097-1.421) | 1.205(1.073-1.352) | 1.153(1.045-1.272) | 1.099(1.015-1.19) | 1.051(0.988-1.117) | 1.015(0.971-1.06) | 0.997(0.976-1.019) | 1.005(0.995-1.014) | 1.038(0.986-1.092) | 1.084(0.99-1.187) | 1.129(1.004-1.268) | 1.155(1.02-1.309) | 1.162(1.012-1.335) |
| lag15 | 1.437(1.214-1.701) | 1.434(1.213-1.696) | 1.416(1.204-1.665) | 1.388(1.188-1.621) | 1.35(1.166-1.563) | 1.303(1.139-1.492) | 1.248(1.106-1.407) | 1.183(1.068-1.311) | 1.117(1.028-1.214) | 1.058(0.992-1.128) | 1.015(0.97-1.062) | 0.995(0.973-1.018) | 1.006(0.996-1.016) | 1.048(0.994-1.105) | 1.106(1.006-1.216) | 1.159(1.027-1.309) | 1.186(1.041-1.351) | 1.184(1.025-1.368) |
| lag16 | 1.528(1.283-1.822) | 1.525(1.281-1.815) | 1.5(1.267-1.776) | 1.464(1.246-1.721) | 1.417(1.217-1.651) | 1.359(1.181-1.564) | 1.291(1.139-1.463) | 1.213(1.09-1.349) | 1.134(1.04-1.236) | 1.065(0.996-1.138) | 1.015(0.968-1.064) | 0.993(0.97-1.017) | 1.008(0.998-1.018) | 1.06(1.003-1.12) | 1.13(1.024-1.246) | 1.192(1.051-1.352) | 1.217(1.064-1.393) | 1.205(1.037-1.4) |
| lag17 | 1.625(1.355-1.95) | 1.62(1.352-1.942) | 1.588(1.333-1.892) | 1.543(1.305-1.825) | 1.485(1.267-1.74) | 1.414(1.222-1.637) | 1.333(1.17-1.518) | 1.241(1.111-1.387) | 1.149(1.05-1.257) | 1.07(0.998-1.147) | 1.014(0.966-1.065) | 0.991(0.967-1.015) | 1.01(0.999-1.02) | 1.072(1.013-1.134) | 1.154(1.043-1.277) | 1.225(1.075-1.395) | 1.248(1.086-1.435) | 1.222(1.046-1.429) |
| lag18 | 1.727(1.431-2.085) | 1.721(1.427-2.075) | 1.679(1.401-2.012) | 1.623(1.364-1.93) | 1.552(1.318-1.829) | 1.469(1.263-1.708) | 1.373(1.2-1.571) | 1.267(1.13-1.422) | 1.163(1.059-1.276) | 1.074(0.999-1.154) | 1.012(0.963-1.065) | 0.988(0.963-1.013) | 1.011(1.001-1.022) | 1.084(1.023-1.149) | 1.179(1.063-1.309) | 1.258(1.101-1.438) | 1.278(1.107-1.475) | 1.236(1.052-1.452) |
| lag19 | 1.834(1.509-2.227) | 1.825(1.504-2.214) | 1.772(1.469-2.136) | 1.703(1.423-2.038) | 1.619(1.366-1.918) | 1.521(1.301-1.778) | 1.411(1.227-1.622) | 1.291(1.147-1.454) | 1.174(1.066-1.293) | 1.077(1-1.16) | 1.01(0.959-1.064) | 0.985(0.96-1.011) | 1.013(1.002-1.025) | 1.097(1.033-1.164) | 1.204(1.083-1.34) | 1.291(1.126-1.48) | 1.305(1.126-1.513) | 1.246(1.055-1.471) |
| lag20 | 1.944(1.589-2.379) | 1.933(1.581-2.362) | 1.866(1.537-2.266) | 1.782(1.479-2.147) | 1.683(1.412-2.007) | 1.57(1.335-1.847) | 1.446(1.25-1.671) | 1.312(1.16-1.485) | 1.184(1.071-1.309) | 1.078(0.998-1.164) | 1.007(0.954-1.063) | 0.982(0.956-1.009) | 1.015(1.004-1.027) | 1.109(1.043-1.179) | 1.229(1.102-1.371) | 1.323(1.149-1.522) | 1.33(1.142-1.548) | 1.251(1.052-1.486) |
| lag21 | 2.058(1.667-2.542) | 2.044(1.657-2.521) | 1.961(1.6-2.402) | 1.86(1.531-2.261) | 1.745(1.451-2.099) | 1.616(1.363-1.917) | 1.477(1.268-1.72) | 1.331(1.168-1.515) | 1.191(1.072-1.324) | 1.078(0.994-1.169) | 1.004(0.949-1.062) | 0.979(0.952-1.007) | 1.017(1.005-1.029) | 1.121(1.052-1.195) | 1.253(1.119-1.404) | 1.353(1.169-1.566) | 1.351(1.153-1.583) | 1.25(1.043-1.498) |

| Table S19 Risk of 1-5y DV with each 1 °C change in lag time from 13.8 °C to 30 °C | | | | | | | | | | | | | | | | | | |
| --- | --- | --- | --- | --- | --- | --- | --- | --- | --- | --- | --- | --- | --- | --- | --- | --- | --- | --- |
| Lag(days) | Temperature(℃), RR(95%CI) | | | | | | | | | | | | | | | | | |
|  | 13.8 | 14 | 15 | 16 | 17 | 18 | 19 | 20 | 21 | 22 | 23 | 24 | 25 | 26 | 27 | 28 | 29 | 30 |
| lag0 | 0.903(0.824-0.988) | 0.909(0.831-0.995) | 0.938(0.86-1.022) | 0.963(0.887-1.045) | 0.983(0.91-1.062) | 0.998(0.929-1.071) | 1.006(0.944-1.073) | 1.008(0.953-1.066) | 1.004(0.958-1.052) | 0.998(0.961-1.037) | 0.994(0.966-1.022) | 0.994(0.98-1.008) | 1.003(0.997-1.009) | 1.022(0.989-1.055) | 1.044(0.984-1.107) | 1.064(0.982-1.151) | 1.075(0.979-1.181) | 1.079(0.962-1.21) |
| lag1 | 0.773(0.714-0.837) | 0.78(0.72-0.844) | 0.812(0.752-0.877) | 0.843(0.783-0.908) | 0.872(0.813-0.936) | 0.898(0.841-0.959) | 0.92(0.866-0.976) | 0.937(0.889-0.988) | 0.951(0.91-0.995) | 0.963(0.928-1) | 0.975(0.948-1.002) | 0.988(0.974-1.002) | 1.005(0.999-1.011) | 1.025(0.992-1.06) | 1.046(0.985-1.112) | 1.064(0.982-1.153) | 1.075(0.979-1.18) | 1.079(0.964-1.207) |
| lag2 | 0.808(0.751-0.87) | 0.816(0.758-0.879) | 0.856(0.796-0.919) | 0.892(0.832-0.956) | 0.923(0.864-0.986) | 0.95(0.893-1.01) | 0.97(0.917-1.025) | 0.982(0.935-1.031) | 0.989(0.948-1.031) | 0.991(0.957-1.026) | 0.992(0.967-1.018) | 0.995(0.982-1.009) | 1.002(0.997-1.008) | 1.015(0.983-1.048) | 1.03(0.973-1.091) | 1.045(0.968-1.127) | 1.055(0.967-1.151) | 1.061(0.955-1.178) |
| lag3 | 0.864(0.798-0.936) | 0.873(0.806-0.945) | 0.913(0.845-0.986) | 0.948(0.88-1.022) | 0.978(0.91-1.05) | 1(0.936-1.069) | 1.014(0.955-1.077) | 1.019(0.966-1.074) | 1.015(0.971-1.063) | 1.008(0.971-1.047) | 1.001(0.973-1.03) | 0.998(0.983-1.013) | 1.002(0.996-1.008) | 1.015(0.98-1.051) | 1.031(0.969-1.097) | 1.046(0.964-1.134) | 1.053(0.96-1.154) | 1.053(0.944-1.175) |
| lag4 | 0.927(0.853-1.007) | 0.935(0.861-1.015) | 0.972(0.897-1.053) | 1.003(0.928-1.084) | 1.027(0.953-1.106) | 1.043(0.973-1.117) | 1.049(0.985-1.117) | 1.046(0.989-1.105) | 1.034(0.986-1.085) | 1.02(0.98-1.061) | 1.006(0.977-1.037) | 0.999(0.983-1.015) | 1.002(0.995-1.009) | 1.016(0.979-1.054) | 1.034(0.968-1.105) | 1.048(0.961-1.142) | 1.051(0.955-1.156) | 1.042(0.931-1.166) |
| lag5 | 0.998(0.912-1.091) | 1.005(0.919-1.098) | 1.036(0.949-1.131) | 1.061(0.975-1.155) | 1.078(0.994-1.169) | 1.086(1.007-1.172) | 1.085(1.013-1.162) | 1.074(1.011-1.141) | 1.055(1.001-1.111) | 1.033(0.989-1.079) | 1.014(0.981-1.048) | 1.002(0.985-1.019) | 1.001(0.994-1.009) | 1.013(0.972-1.054) | 1.028(0.956-1.105) | 1.039(0.946-1.14) | 1.036(0.934-1.149) | 1.021(0.905-1.152) |
| lag6 | 1.077(0.978-1.185) | 1.083(0.984-1.191) | 1.108(1.009-1.216) | 1.126(1.029-1.232) | 1.135(1.042-1.238) | 1.136(1.048-1.231) | 1.127(1.047-1.212) | 1.107(1.038-1.181) | 1.08(1.022-1.141) | 1.051(1.004-1.1) | 1.025(0.99-1.061) | 1.006(0.988-1.025) | 1(0.992-1.008) | 1.005(0.963-1.049) | 1.015(0.941-1.094) | 1.019(0.925-1.124) | 1.012(0.909-1.126) | 0.992(0.874-1.125) |
| lag7 | 1.164(1.052-1.289) | 1.169(1.057-1.293) | 1.188(1.076-1.311) | 1.198(1.089-1.318) | 1.2(1.096-1.314) | 1.192(1.095-1.297) | 1.174(1.087-1.268) | 1.145(1.071-1.225) | 1.11(1.047-1.175) | 1.072(1.022-1.124) | 1.038(1.001-1.076) | 1.012(0.993-1.031) | 0.998(0.99-1.006) | 0.996(0.953-1.041) | 0.998(0.923-1.08) | 0.997(0.901-1.103) | 0.985(0.882-1.1) | 0.962(0.845-1.096) |
| lag8 | 1.26(1.131-1.404) | 1.263(1.135-1.407) | 1.276(1.149-1.417) | 1.279(1.155-1.416) | 1.273(1.155-1.402) | 1.255(1.147-1.374) | 1.227(1.131-1.332) | 1.189(1.107-1.276) | 1.142(1.075-1.214) | 1.095(1.041-1.151) | 1.052(1.013-1.092) | 1.017(0.997-1.038) | 0.996(0.987-1.004) | 0.987(0.942-1.034) | 0.984(0.906-1.068) | 0.977(0.879-1.087) | 0.961(0.856-1.08) | 0.936(0.816-1.073) |
| lag9 | 1.363(1.215-1.53) | 1.366(1.218-1.532) | 1.373(1.227-1.535) | 1.369(1.228-1.525) | 1.354(1.221-1.5) | 1.326(1.205-1.46) | 1.287(1.18-1.404) | 1.236(1.146-1.334) | 1.178(1.104-1.256) | 1.119(1.061-1.18) | 1.065(1.024-1.109) | 1.022(1.001-1.044) | 0.994(0.985-1.003) | 0.981(0.934-1.03) | 0.973(0.892-1.061) | 0.963(0.862-1.077) | 0.944(0.836-1.067) | 0.916(0.793-1.058) |
| lag10 | 1.475(1.306-1.665) | 1.476(1.308-1.666) | 1.478(1.314-1.664) | 1.468(1.309-1.645) | 1.443(1.295-1.609) | 1.405(1.27-1.555) | 1.353(1.235-1.483) | 1.288(1.189-1.395) | 1.215(1.135-1.3) | 1.143(1.081-1.208) | 1.078(1.034-1.124) | 1.027(1.005-1.049) | 0.993(0.984-1.003) | 0.977(0.928-1.028) | 0.967(0.883-1.06) | 0.956(0.851-1.074) | 0.935(0.823-1.062) | 0.903(0.777-1.05) |
| lag11 | 1.594(1.404-1.81) | 1.595(1.405-1.81) | 1.593(1.408-1.803) | 1.576(1.398-1.776) | 1.542(1.376-1.727) | 1.491(1.342-1.657) | 1.424(1.295-1.567) | 1.343(1.236-1.46) | 1.253(1.168-1.345) | 1.166(1.1-1.236) | 1.09(1.043-1.138) | 1.03(1.007-1.054) | 0.992(0.983-1.002) | 0.975(0.925-1.029) | 0.967(0.88-1.063) | 0.956(0.847-1.079) | 0.933(0.817-1.064) | 0.897(0.767-1.049) |
| lag12 | 1.721(1.508-1.965) | 1.722(1.509-1.965) | 1.716(1.509-1.952) | 1.692(1.494-1.916) | 1.647(1.463-1.854) | 1.583(1.418-1.767) | 1.5(1.358-1.656) | 1.4(1.283-1.526) | 1.292(1.2-1.39) | 1.189(1.119-1.262) | 1.1(1.051-1.151) | 1.033(1.009-1.057) | 0.992(0.982-1.002) | 0.976(0.924-1.032) | 0.971(0.881-1.071) | 0.962(0.849-1.09) | 0.937(0.818-1.073) | 0.896(0.763-1.053) |
| lag13 | 1.857(1.618-2.131) | 1.858(1.62-2.131) | 1.848(1.616-2.113) | 1.815(1.595-2.066) | 1.759(1.555-1.989) | 1.679(1.497-1.882) | 1.577(1.423-1.749) | 1.457(1.332-1.595) | 1.329(1.232-1.435) | 1.209(1.136-1.288) | 1.109(1.058-1.162) | 1.034(1.01-1.06) | 0.992(0.982-1.003) | 0.98(0.925-1.038) | 0.979(0.885-1.084) | 0.973(0.855-1.107) | 0.946(0.822-1.089) | 0.9(0.762-1.063) |
| lag14 | 2.002(1.735-2.31) | 2.002(1.736-2.309) | 1.987(1.729-2.285) | 1.945(1.7-2.226) | 1.875(1.65-2.131) | 1.778(1.578-2.003) | 1.656(1.487-1.844) | 1.514(1.378-1.663) | 1.365(1.261-1.478) | 1.228(1.151-1.311) | 1.116(1.063-1.171) | 1.035(1.009-1.062) | 0.993(0.982-1.004) | 0.985(0.928-1.045) | 0.991(0.892-1.101) | 0.989(0.864-1.131) | 0.96(0.83-1.11) | 0.907(0.763-1.078) |
| lag15 | 2.156(1.857-2.503) | 2.155(1.858-2.501) | 2.134(1.846-2.467) | 2.08(1.808-2.393) | 1.994(1.745-2.279) | 1.878(1.659-2.126) | 1.734(1.55-1.94) | 1.569(1.422-1.73) | 1.399(1.287-1.519) | 1.245(1.163-1.332) | 1.121(1.065-1.179) | 1.035(1.008-1.063) | 0.994(0.982-1.005) | 0.992(0.932-1.055) | 1.006(0.901-1.122) | 1.008(0.877-1.159) | 0.977(0.84-1.137) | 0.916(0.765-1.096) |
| lag16 | 2.32(1.986-2.709) | 2.318(1.986-2.705) | 2.287(1.966-2.659) | 2.219(1.918-2.567) | 2.115(1.841-2.43) | 1.977(1.737-2.249) | 1.809(1.61-2.033) | 1.62(1.463-1.794) | 1.428(1.31-1.557) | 1.258(1.172-1.35) | 1.124(1.066-1.185) | 1.034(1.006-1.063) | 0.995(0.983-1.007) | 1.001(0.938-1.067) | 1.023(0.913-1.146) | 1.031(0.892-1.192) | 0.998(0.853-1.168) | 0.927(0.769-1.118) |
| lag17 | 2.493(2.123-2.929) | 2.489(2.121-2.922) | 2.445(2.091-2.859) | 2.36(2.028-2.745) | 2.234(1.935-2.581) | 2.073(1.813-2.37) | 1.881(1.666-2.123) | 1.667(1.5-1.853) | 1.454(1.33-1.59) | 1.269(1.179-1.365) | 1.126(1.066-1.189) | 1.033(1.004-1.062) | 0.996(0.984-1.009) | 1.01(0.945-1.08) | 1.043(0.927-1.173) | 1.058(0.91-1.228) | 1.021(0.868-1.202) | 0.939(0.774-1.14) |
| lag18 | 2.677(2.266-3.162) | 2.67(2.262-3.152) | 2.608(2.218-3.066) | 2.501(2.138-2.925) | 2.351(2.026-2.73) | 2.164(1.883-2.486) | 1.946(1.717-2.206) | 1.708(1.531-1.906) | 1.475(1.345-1.618) | 1.275(1.183-1.376) | 1.125(1.063-1.19) | 1.03(1.001-1.061) | 0.998(0.985-1.011) | 1.022(0.954-1.094) | 1.065(0.944-1.202) | 1.087(0.932-1.267) | 1.047(0.885-1.237) | 0.953(0.78-1.164) |
| lag19 | 2.871(2.417-3.411) | 2.86(2.409-3.395) | 2.774(2.346-3.28) | 2.641(2.246-3.106) | 2.464(2.111-2.875) | 2.248(1.947-2.596) | 2.004(1.76-2.282) | 1.742(1.556-1.951) | 1.49(1.355-1.64) | 1.279(1.183-1.382) | 1.122(1.059-1.189) | 1.027(0.997-1.058) | 1(0.987-1.013) | 1.034(0.964-1.109) | 1.09(0.963-1.234) | 1.118(0.955-1.309) | 1.074(0.904-1.276) | 0.966(0.786-1.188) |
| lag20 | 3.076(2.573-3.678) | 3.059(2.56-3.655) | 2.943(2.473-3.502) | 2.778(2.348-3.287) | 2.569(2.189-3.016) | 2.324(2.002-2.699) | 2.053(1.794-2.35) | 1.769(1.573-1.99) | 1.5(1.359-1.657) | 1.278(1.179-1.385) | 1.117(1.052-1.186) | 1.023(0.992-1.055) | 1.002(0.989-1.016) | 1.048(0.975-1.126) | 1.117(0.983-1.268) | 1.153(0.98-1.356) | 1.103(0.924-1.317) | 0.98(0.791-1.214) |
| lag21 | 3.292(2.731-3.969) | 3.267(2.712-3.936) | 3.113(2.595-3.734) | 2.911(2.441-3.471) | 2.667(2.255-3.154) | 2.391(2.045-2.795) | 2.093(1.817-2.411) | 1.788(1.58-2.022) | 1.504(1.356-1.668) | 1.274(1.171-1.386) | 1.11(1.043-1.182) | 1.018(0.986-1.051) | 1.005(0.991-1.019) | 1.063(0.986-1.145) | 1.146(1.004-1.308) | 1.19(1.005-1.408) | 1.134(0.942-1.365) | 0.994(0.794-1.244) |

| Table S20 Risk of 6-20y DV with each 1 °C change in lag time from 13.8 °C to 30 °C | | | | | | | | | | | | | | | | | | |
| --- | --- | --- | --- | --- | --- | --- | --- | --- | --- | --- | --- | --- | --- | --- | --- | --- | --- | --- |
| Lag(days) | Temperature(℃), RR(95%CI) | | | | | | | | | | | | | | | | | |
|  | 13.8 | 14 | 15 | 16 | 17 | 18 | 19 | 20 | 21 | 22 | 23 | 24 | 25 | 26 | 27 | 28 | 29 | 30 |
| lag0 | 0.878(0.677-1.137) | 0.883(0.683-1.142) | 0.912(0.713-1.165) | 0.937(0.742-1.182) | 0.959(0.771-1.192) | 0.977(0.799-1.193) | 0.99(0.827-1.184) | 0.997(0.855-1.164) | 1(0.88-1.137) | 1.001(0.904-1.107) | 1(0.93-1.075) | 0.999(0.963-1.036) | 1.001(0.985-1.016) | 1.008(0.927-1.095) | 1.024(0.88-1.191) | 1.055(0.86-1.293) | 1.105(0.87-1.404) | 1.176(0.883-1.568) |
| lag1 | 0.885(0.704-1.112) | 0.888(0.707-1.114) | 0.903(0.725-1.124) | 0.916(0.742-1.13) | 0.928(0.76-1.132) | 0.938(0.78-1.129) | 0.947(0.802-1.119) | 0.954(0.825-1.102) | 0.959(0.849-1.083) | 0.965(0.875-1.065) | 0.974(0.906-1.046) | 0.987(0.951-1.024) | 1.006(0.99-1.022) | 1.031(0.946-1.123) | 1.057(0.905-1.234) | 1.076(0.875-1.323) | 1.084(0.855-1.374) | 1.08(0.817-1.428) |
| lag2 | 0.889(0.718-1.101) | 0.897(0.725-1.109) | 0.935(0.761-1.15) | 0.969(0.794-1.182) | 0.997(0.825-1.203) | 1.018(0.855-1.212) | 1.031(0.881-1.206) | 1.035(0.904-1.185) | 1.031(0.921-1.155) | 1.023(0.934-1.12) | 1.013(0.948-1.083) | 1.004(0.97-1.039) | 0.999(0.984-1.014) | 1.001(0.923-1.086) | 1.011(0.873-1.172) | 1.032(0.85-1.254) | 1.067(0.856-1.33) | 1.114(0.86-1.443) |
| lag3 | 0.964(0.766-1.213) | 0.973(0.775-1.223) | 1.017(0.815-1.27) | 1.055(0.852-1.306) | 1.084(0.885-1.328) | 1.104(0.915-1.332) | 1.112(0.939-1.316) | 1.108(0.958-1.282) | 1.094(0.969-1.235) | 1.072(0.973-1.181) | 1.046(0.974-1.123) | 1.019(0.982-1.057) | 0.994(0.978-1.01) | 0.975(0.895-1.062) | 0.969(0.83-1.131) | 0.982(0.802-1.204) | 1.023(0.815-1.284) | 1.09(0.838-1.42) |
| lag4 | 1.065(0.841-1.349) | 1.074(0.849-1.358) | 1.111(0.884-1.397) | 1.141(0.915-1.423) | 1.162(0.942-1.433) | 1.173(0.965-1.424) | 1.172(0.984-1.395) | 1.159(0.997-1.347) | 1.135(1.002-1.286) | 1.103(0.998-1.22) | 1.067(0.991-1.148) | 1.028(0.99-1.068) | 0.99(0.974-1.007) | 0.957(0.876-1.047) | 0.938(0.799-1.101) | 0.942(0.764-1.16) | 0.976(0.775-1.229) | 1.042(0.8-1.358) |
| lag5 | 1.151(0.891-1.487) | 1.158(0.897-1.494) | 1.186(0.924-1.521) | 1.206(0.948-1.534) | 1.217(0.969-1.53) | 1.219(0.986-1.506) | 1.21(1-1.463) | 1.19(1.01-1.402) | 1.16(1.012-1.329) | 1.122(1.006-1.251) | 1.08(0.997-1.169) | 1.034(0.992-1.078) | 0.988(0.971-1.006) | 0.946(0.859-1.043) | 0.918(0.772-1.093) | 0.914(0.729-1.145) | 0.942(0.735-1.207) | 1.002(0.755-1.33) |
| lag6 | 1.21(0.92-1.59) | 1.214(0.925-1.595) | 1.234(0.946-1.611) | 1.247(0.965-1.612) | 1.251(0.98-1.597) | 1.246(0.994-1.563) | 1.232(1.005-1.509) | 1.207(1.013-1.437) | 1.173(1.015-1.356) | 1.132(1.009-1.27) | 1.086(0.999-1.182) | 1.037(0.993-1.083) | 0.987(0.969-1.006) | 0.94(0.849-1.041) | 0.907(0.756-1.089) | 0.898(0.71-1.137) | 0.922(0.713-1.192) | 0.978(0.73-1.311) |
| lag7 | 1.249(0.934-1.669) | 1.252(0.938-1.672) | 1.267(0.955-1.681) | 1.274(0.97-1.674) | 1.274(0.983-1.65) | 1.264(0.995-1.607) | 1.245(1.005-1.544) | 1.217(1.012-1.464) | 1.18(1.014-1.375) | 1.137(1.008-1.283) | 1.089(0.997-1.189) | 1.038(0.993-1.086) | 0.986(0.967-1.006) | 0.938(0.844-1.043) | 0.904(0.748-1.092) | 0.893(0.699-1.139) | 0.913(0.701-1.189) | 0.965(0.714-1.304) |
| lag8 | 1.277(0.937-1.742) | 1.281(0.94-1.744) | 1.292(0.956-1.748) | 1.297(0.969-1.736) | 1.293(0.98-1.704) | 1.28(0.991-1.653) | 1.257(1-1.581) | 1.226(1.007-1.492) | 1.186(1.008-1.394) | 1.14(1.003-1.295) | 1.09(0.993-1.196) | 1.038(0.99-1.089) | 0.987(0.966-1.007) | 0.939(0.84-1.051) | 0.906(0.741-1.107) | 0.894(0.692-1.156) | 0.912(0.691-1.203) | 0.959(0.699-1.315) |
| lag9 | 1.304(0.936-1.815) | 1.307(0.94-1.817) | 1.318(0.955-1.819) | 1.321(0.967-1.803) | 1.314(0.978-1.766) | 1.298(0.988-1.706) | 1.272(0.996-1.624) | 1.236(1.002-1.524) | 1.192(1.003-1.416) | 1.142(0.996-1.309) | 1.09(0.987-1.203) | 1.037(0.986-1.091) | 0.987(0.966-1.009) | 0.943(0.837-1.063) | 0.912(0.738-1.128) | 0.902(0.687-1.183) | 0.917(0.684-1.229) | 0.957(0.686-1.336) |
| lag10 | 1.333(0.939-1.892) | 1.336(0.942-1.894) | 1.348(0.958-1.896) | 1.35(0.971-1.877) | 1.342(0.982-1.834) | 1.323(0.991-1.766) | 1.293(0.998-1.674) | 1.251(1.002-1.561) | 1.2(1-1.44) | 1.145(0.992-1.322) | 1.089(0.981-1.209) | 1.036(0.982-1.093) | 0.988(0.966-1.011) | 0.949(0.837-1.076) | 0.922(0.738-1.153) | 0.914(0.687-1.215) | 0.927(0.682-1.258) | 0.96(0.677-1.36) |
| lag11 | 1.367(0.947-1.974) | 1.371(0.951-1.977) | 1.385(0.968-1.98) | 1.387(0.982-1.959) | 1.377(0.993-1.911) | 1.355(1.001-1.834) | 1.319(1.006-1.729) | 1.27(1.007-1.602) | 1.211(1.001-1.466) | 1.149(0.989-1.336) | 1.089(0.977-1.214) | 1.034(0.979-1.094) | 0.989(0.966-1.013) | 0.956(0.839-1.089) | 0.935(0.742-1.179) | 0.93(0.692-1.249) | 0.94(0.685-1.289) | 0.965(0.673-1.383) |
| lag12 | 1.408(0.96-2.065) | 1.412(0.964-2.069) | 1.428(0.984-2.074) | 1.431(0.998-2.051) | 1.419(1.008-1.997) | 1.392(1.015-1.909) | 1.35(1.017-1.79) | 1.292(1.014-1.646) | 1.224(1.004-1.493) | 1.154(0.987-1.35) | 1.089(0.973-1.219) | 1.033(0.975-1.094) | 0.991(0.966-1.016) | 0.964(0.842-1.103) | 0.95(0.748-1.207) | 0.948(0.699-1.287) | 0.956(0.691-1.324) | 0.973(0.672-1.409) |
| lag13 | 1.455(0.976-2.169) | 1.461(0.981-2.174) | 1.479(1.002-2.181) | 1.481(1.017-2.156) | 1.467(1.027-2.094) | 1.434(1.032-1.994) | 1.384(1.031-1.858) | 1.317(1.023-1.695) | 1.239(1.008-1.524) | 1.16(0.986-1.365) | 1.089(0.968-1.224) | 1.031(0.971-1.094) | 0.992(0.967-1.018) | 0.972(0.845-1.118) | 0.967(0.754-1.238) | 0.969(0.707-1.329) | 0.976(0.698-1.365) | 0.984(0.672-1.442) |
| lag14 | 1.511(0.997-2.29) | 1.516(1.002-2.295) | 1.536(1.024-2.303) | 1.537(1.039-2.273) | 1.519(1.048-2.202) | 1.48(1.05-2.087) | 1.421(1.045-1.933) | 1.344(1.033-1.748) | 1.254(1.011-1.556) | 1.166(0.985-1.382) | 1.089(0.964-1.23) | 1.029(0.967-1.095) | 0.993(0.967-1.02) | 0.981(0.849-1.134) | 0.984(0.761-1.272) | 0.992(0.715-1.375) | 0.997(0.704-1.412) | 0.999(0.672-1.484) |
| lag15 | 1.575(1.021-2.428) | 1.581(1.027-2.434) | 1.6(1.049-2.44) | 1.599(1.064-2.404) | 1.576(1.07-2.32) | 1.529(1.069-2.188) | 1.46(1.06-2.012) | 1.371(1.042-1.804) | 1.27(1.014-1.591) | 1.173(0.983-1.399) | 1.089(0.959-1.236) | 1.028(0.963-1.096) | 0.994(0.967-1.023) | 0.99(0.851-1.15) | 1.001(0.766-1.307) | 1.015(0.723-1.426) | 1.021(0.711-1.466) | 1.017(0.674-1.536) |
| lag16 | 1.649(1.051-2.586) | 1.654(1.056-2.591) | 1.672(1.078-2.592) | 1.666(1.09-2.546) | 1.636(1.094-2.446) | 1.58(1.089-2.294) | 1.5(1.075-2.094) | 1.398(1.051-1.861) | 1.286(1.017-1.625) | 1.179(0.981-1.416) | 1.089(0.955-1.243) | 1.026(0.959-1.097) | 0.996(0.967-1.025) | 0.998(0.853-1.166) | 1.018(0.771-1.342) | 1.039(0.731-1.477) | 1.047(0.719-1.523) | 1.039(0.677-1.595) |
| lag17 | 1.734(1.087-2.765) | 1.739(1.092-2.768) | 1.751(1.111-2.76) | 1.738(1.12-2.698) | 1.699(1.119-2.579) | 1.632(1.109-2.403) | 1.54(1.089-2.177) | 1.425(1.06-1.918) | 1.301(1.02-1.659) | 1.185(0.979-1.433) | 1.09(0.951-1.249) | 1.025(0.956-1.098) | 0.997(0.967-1.027) | 1.005(0.856-1.181) | 1.034(0.777-1.375) | 1.062(0.739-1.528) | 1.073(0.729-1.581) | 1.065(0.683-1.661) |
| lag18 | 1.831(1.13-2.967) | 1.835(1.135-2.968) | 1.839(1.149-2.943) | 1.815(1.152-2.861) | 1.764(1.145-2.717) | 1.684(1.129-2.513) | 1.579(1.104-2.259) | 1.451(1.068-1.973) | 1.315(1.023-1.691) | 1.19(0.978-1.449) | 1.09(0.947-1.255) | 1.023(0.953-1.099) | 0.998(0.968-1.029) | 1.012(0.858-1.195) | 1.048(0.782-1.406) | 1.085(0.747-1.575) | 1.102(0.74-1.64) | 1.095(0.692-1.733) |
| lag19 | 1.943(1.181-3.198) | 1.944(1.183-3.195) | 1.935(1.19-3.145) | 1.897(1.186-3.035) | 1.831(1.172-2.861) | 1.736(1.148-2.626) | 1.616(1.116-2.341) | 1.475(1.074-2.026) | 1.328(1.024-1.722) | 1.195(0.976-1.465) | 1.091(0.943-1.261) | 1.022(0.95-1.1) | 0.999(0.968-1.03) | 1.018(0.859-1.206) | 1.061(0.786-1.434) | 1.107(0.755-1.621) | 1.131(0.752-1.701) | 1.131(0.705-1.814) |
| lag20 | 2.071(1.236-3.47) | 2.069(1.237-3.46) | 2.041(1.234-3.375) | 1.984(1.22-3.229) | 1.899(1.196-3.017) | 1.787(1.164-2.744) | 1.652(1.125-2.425) | 1.497(1.077-2.081) | 1.34(1.024-1.754) | 1.2(0.972-1.481) | 1.091(0.939-1.268) | 1.022(0.947-1.102) | 0.999(0.967-1.032) | 1.023(0.859-1.218) | 1.073(0.787-1.461) | 1.127(0.761-1.669) | 1.161(0.762-1.769) | 1.171(0.717-1.912) |
| lag21 | 2.218(1.295-3.8) | 2.211(1.293-3.78) | 2.157(1.276-3.647) | 2.076(1.249-3.452) | 1.969(1.214-3.192) | 1.837(1.174-2.876) | 1.685(1.128-2.518) | 1.517(1.075-2.141) | 1.35(1.019-1.79) | 1.204(0.966-1.501) | 1.092(0.933-1.277) | 1.021(0.944-1.105) | 1(0.966-1.034) | 1.026(0.855-1.231) | 1.082(0.784-1.494) | 1.145(0.76-1.726) | 1.192(0.766-1.853) | 1.217(0.725-2.04) |

| Table S21 Risk of >20y DV with each 1 °C change in lag time from 13.8 °C to 30 °C | | | | | | | | | | | | | | | | | | |
| --- | --- | --- | --- | --- | --- | --- | --- | --- | --- | --- | --- | --- | --- | --- | --- | --- | --- | --- |
| Lag(days) | Temperature(℃), RR(95%CI) | | | | | | | | | | | | | | | | | |
|  | 13.8 | 14 | 15 | 16 | 17 | 18 | 19 | 20 | 21 | 22 | 23 | 24 | 25 | 26 | 27 | 28 | 29 | 30 |
| lag0 | 0.716(0.588-0.872) | 0.719(0.592-0.875) | 0.736(0.61-0.888) | 0.754(0.631-0.902) | 0.774(0.654-0.916) | 0.795(0.681-0.929) | 0.819(0.712-0.942) | 0.844(0.747-0.953) | 0.872(0.786-0.966) | 0.902(0.83-0.98) | 0.935(0.88-0.993) | 0.971(0.942-1.001) | 1.011(0.998-1.024) | 1.053(0.982-1.13) | 1.095(0.965-1.243) | 1.132(0.955-1.342) | 1.161(0.952-1.415) | 1.18(0.933-1.493) |
| lag1 | 0.859(0.72-1.024) | 0.864(0.726-1.029) | 0.89(0.751-1.055) | 0.913(0.776-1.075) | 0.934(0.8-1.09) | 0.95(0.823-1.098) | 0.963(0.845-1.097) | 0.971(0.865-1.089) | 0.975(0.884-1.075) | 0.978(0.902-1.06) | 0.982(0.925-1.043) | 0.99(0.96-1.021) | 1.005(0.991-1.018) | 1.025(0.954-1.102) | 1.047(0.92-1.191) | 1.061(0.894-1.259) | 1.063(0.874-1.291) | 1.052(0.837-1.321) |
| lag2 | 0.817(0.694-0.962) | 0.823(0.699-0.967) | 0.849(0.725-0.995) | 0.875(0.751-1.019) | 0.899(0.778-1.039) | 0.921(0.805-1.054) | 0.941(0.833-1.062) | 0.958(0.862-1.065) | 0.973(0.889-1.064) | 0.984(0.914-1.06) | 0.992(0.939-1.049) | 0.998(0.97-1.027) | 1(0.988-1.013) | 1.001(0.935-1.071) | 1.004(0.888-1.134) | 1.013(0.862-1.189) | 1.031(0.861-1.236) | 1.059(0.858-1.309) |
| lag3 | 0.782(0.657-0.931) | 0.788(0.662-0.938) | 0.818(0.69-0.97) | 0.847(0.719-0.998) | 0.875(0.749-1.022) | 0.901(0.779-1.041) | 0.925(0.811-1.054) | 0.946(0.844-1.061) | 0.965(0.876-1.064) | 0.98(0.905-1.062) | 0.991(0.934-1.052) | 0.998(0.968-1.03) | 1(0.987-1.014) | 0.998(0.928-1.073) | 0.995(0.873-1.134) | 0.995(0.838-1.181) | 1.001(0.827-1.212) | 1.013(0.814-1.262) |
| lag4 | 0.775(0.648-0.928) | 0.782(0.654-0.936) | 0.815(0.684-0.971) | 0.847(0.715-1.004) | 0.877(0.746-1.03) | 0.904(0.777-1.051) | 0.928(0.81-1.064) | 0.948(0.841-1.069) | 0.965(0.872-1.069) | 0.978(0.899-1.064) | 0.989(0.928-1.053) | 0.996(0.964-1.029) | 1.001(0.987-1.016) | 1.004(0.929-1.085) | 1.003(0.873-1.152) | 0.995(0.831-1.192) | 0.981(0.805-1.196) | 0.961(0.767-1.203) |
| lag5 | 0.792(0.651-0.963) | 0.799(0.657-0.971) | 0.834(0.688-1.009) | 0.866(0.719-1.042) | 0.895(0.75-1.069) | 0.921(0.781-1.086) | 0.943(0.812-1.095) | 0.96(0.842-1.094) | 0.972(0.869-1.086) | 0.981(0.895-1.075) | 0.988(0.922-1.058) | 0.994(0.959-1.031) | 1.002(0.987-1.018) | 1.011(0.929-1.1) | 1.013(0.871-1.179) | 1.004(0.825-1.222) | 0.978(0.789-1.212) | 0.938(0.736-1.195) |
| lag6 | 0.826(0.669-1.018) | 0.833(0.676-1.027) | 0.867(0.706-1.064) | 0.898(0.736-1.096) | 0.926(0.766-1.118) | 0.948(0.795-1.131) | 0.966(0.824-1.133) | 0.977(0.851-1.123) | 0.984(0.874-1.107) | 0.987(0.896-1.087) | 0.99(0.92-1.064) | 0.994(0.957-1.032) | 1.003(0.987-1.019) | 1.015(0.929-1.109) | 1.022(0.872-1.198) | 1.015(0.827-1.246) | 0.988(0.79-1.234) | 0.942(0.732-1.212) |
| lag7 | 0.869(0.695-1.086) | 0.876(0.701-1.094) | 0.909(0.731-1.13) | 0.938(0.76-1.159) | 0.963(0.787-1.177) | 0.982(0.814-1.183) | 0.994(0.84-1.176) | 1(0.863-1.158) | 1(0.883-1.131) | 0.997(0.901-1.103) | 0.994(0.922-1.072) | 0.995(0.957-1.035) | 1.003(0.986-1.02) | 1.016(0.927-1.115) | 1.027(0.871-1.212) | 1.025(0.828-1.267) | 1.001(0.795-1.26) | 0.959(0.739-1.244) |
| lag8 | 0.916(0.722-1.163) | 0.923(0.728-1.171) | 0.955(0.756-1.205) | 0.982(0.784-1.23) | 1.003(0.809-1.243) | 1.018(0.834-1.242) | 1.025(0.857-1.227) | 1.025(0.877-1.198) | 1.018(0.893-1.161) | 1.009(0.906-1.123) | 1(0.924-1.083) | 0.997(0.957-1.039) | 1.002(0.984-1.021) | 1.016(0.921-1.12) | 1.028(0.864-1.223) | 1.029(0.823-1.287) | 1.011(0.794-1.288) | 0.976(0.742-1.283) |
| lag9 | 0.963(0.747-1.242) | 0.97(0.753-1.25) | 1.001(0.78-1.283) | 1.026(0.807-1.305) | 1.045(0.831-1.313) | 1.056(0.854-1.305) | 1.058(0.874-1.281) | 1.052(0.891-1.241) | 1.039(0.904-1.194) | 1.023(0.913-1.145) | 1.008(0.927-1.097) | 1(0.957-1.045) | 1.002(0.983-1.021) | 1.013(0.913-1.123) | 1.025(0.853-1.232) | 1.029(0.813-1.303) | 1.016(0.787-1.31) | 0.986(0.739-1.316) |
| lag10 | 1.007(0.77-1.317) | 1.014(0.775-1.325) | 1.045(0.803-1.359) | 1.069(0.829-1.379) | 1.086(0.853-1.383) | 1.094(0.874-1.368) | 1.092(0.893-1.336) | 1.08(0.907-1.286) | 1.06(0.916-1.228) | 1.038(0.921-1.169) | 1.017(0.931-1.111) | 1.003(0.958-1.051) | 1.001(0.981-1.021) | 1.009(0.905-1.124) | 1.02(0.841-1.236) | 1.024(0.799-1.311) | 1.012(0.776-1.321) | 0.987(0.73-1.335) |
| lag11 | 1.048(0.791-1.388) | 1.055(0.797-1.397) | 1.087(0.826-1.432) | 1.112(0.852-1.451) | 1.127(0.875-1.451) | 1.132(0.896-1.431) | 1.126(0.912-1.39) | 1.109(0.924-1.331) | 1.082(0.929-1.261) | 1.053(0.93-1.192) | 1.026(0.936-1.125) | 1.007(0.96-1.056) | 1(0.979-1.02) | 1.004(0.897-1.124) | 1.013(0.829-1.238) | 1.016(0.786-1.313) | 1.004(0.762-1.323) | 0.979(0.717-1.338) |
| lag12 | 1.088(0.811-1.459) | 1.096(0.818-1.468) | 1.13(0.848-1.505) | 1.155(0.875-1.524) | 1.17(0.899-1.522) | 1.172(0.918-1.496) | 1.162(0.933-1.447) | 1.139(0.942-1.377) | 1.105(0.943-1.296) | 1.068(0.939-1.215) | 1.035(0.941-1.139) | 1.01(0.961-1.061) | 0.999(0.977-1.02) | 1.001(0.891-1.125) | 1.008(0.819-1.24) | 1.009(0.774-1.315) | 0.995(0.749-1.322) | 0.967(0.701-1.335) |
| lag13 | 1.13(0.832-1.534) | 1.138(0.839-1.544) | 1.175(0.872-1.584) | 1.201(0.9-1.603) | 1.215(0.924-1.598) | 1.215(0.942-1.566) | 1.2(0.955-1.507) | 1.169(0.96-1.425) | 1.128(0.956-1.331) | 1.083(0.948-1.239) | 1.043(0.944-1.152) | 1.013(0.962-1.066) | 0.998(0.976-1.021) | 0.999(0.885-1.128) | 1.006(0.811-1.247) | 1.005(0.764-1.323) | 0.988(0.736-1.326) | 0.956(0.685-1.333) |
| lag14 | 1.176(0.856-1.617) | 1.186(0.863-1.628) | 1.225(0.898-1.672) | 1.253(0.927-1.692) | 1.265(0.951-1.683) | 1.261(0.968-1.644) | 1.24(0.978-1.573) | 1.202(0.978-1.476) | 1.151(0.969-1.367) | 1.097(0.954-1.261) | 1.049(0.946-1.163) | 1.014(0.961-1.07) | 0.998(0.975-1.021) | 1(0.882-1.134) | 1.008(0.806-1.261) | 1.008(0.758-1.341) | 0.987(0.727-1.34) | 0.947(0.67-1.339) |
| lag15 | 1.23(0.883-1.713) | 1.24(0.892-1.725) | 1.283(0.928-1.773) | 1.311(0.959-1.792) | 1.321(0.982-1.779) | 1.313(0.997-1.73) | 1.284(1.002-1.645) | 1.235(0.997-1.531) | 1.173(0.98-1.404) | 1.109(0.959-1.283) | 1.054(0.946-1.174) | 1.015(0.96-1.073) | 0.998(0.975-1.023) | 1.005(0.882-1.145) | 1.018(0.807-1.284) | 1.019(0.757-1.372) | 0.994(0.724-1.366) | 0.945(0.66-1.355) |
| lag16 | 1.294(0.917-1.824) | 1.305(0.926-1.838) | 1.35(0.965-1.889) | 1.378(0.996-1.908) | 1.386(1.017-1.887) | 1.371(1.029-1.825) | 1.332(1.029-1.723) | 1.271(1.016-1.589) | 1.195(0.991-1.441) | 1.12(0.963-1.303) | 1.056(0.945-1.181) | 1.013(0.956-1.074) | 1(0.975-1.025) | 1.013(0.885-1.161) | 1.035(0.813-1.317) | 1.041(0.765-1.417) | 1.013(0.728-1.409) | 0.953(0.655-1.385) |
| lag17 | 1.371(0.96-1.957) | 1.382(0.969-1.972) | 1.431(1.01-2.026) | 1.457(1.041-2.041) | 1.46(1.06-2.011) | 1.436(1.067-1.932) | 1.385(1.06-1.809) | 1.308(1.038-1.648) | 1.216(1.002-1.477) | 1.128(0.964-1.319) | 1.056(0.941-1.185) | 1.011(0.952-1.073) | 1.002(0.976-1.028) | 1.027(0.893-1.182) | 1.062(0.828-1.363) | 1.077(0.783-1.481) | 1.045(0.743-1.47) | 0.971(0.659-1.432) |
| lag18 | 1.464(1.014-2.115) | 1.477(1.024-2.131) | 1.527(1.066-2.187) | 1.551(1.095-2.196) | 1.546(1.11-2.152) | 1.51(1.111-2.053) | 1.443(1.095-1.902) | 1.347(1.06-1.711) | 1.237(1.012-1.511) | 1.134(0.964-1.332) | 1.053(0.935-1.186) | 1.006(0.946-1.07) | 1.005(0.978-1.032) | 1.046(0.906-1.208) | 1.101(0.852-1.422) | 1.129(0.814-1.565) | 1.094(0.77-1.554) | 1.004(0.672-1.499) |
| lag19 | 1.579(1.08-2.308) | 1.592(1.09-2.324) | 1.642(1.133-2.38) | 1.662(1.16-2.38) | 1.646(1.17-2.317) | 1.595(1.161-2.19) | 1.507(1.133-2.005) | 1.388(1.084-1.777) | 1.256(1.022-1.544) | 1.136(0.962-1.342) | 1.047(0.926-1.183) | 1(0.939-1.065) | 1.008(0.981-1.036) | 1.072(0.924-1.242) | 1.152(0.887-1.498) | 1.2(0.858-1.677) | 1.164(0.811-1.669) | 1.053(0.697-1.593) |
| lag20 | 1.719(1.16-2.546) | 1.732(1.171-2.564) | 1.782(1.213-2.616) | 1.794(1.236-2.602) | 1.764(1.238-2.513) | 1.691(1.218-2.349) | 1.579(1.175-2.122) | 1.432(1.108-1.85) | 1.274(1.029-1.578) | 1.136(0.956-1.35) | 1.038(0.914-1.177) | 0.992(0.929-1.058) | 1.013(0.985-1.042) | 1.104(0.948-1.285) | 1.22(0.931-1.597) | 1.294(0.917-1.827) | 1.258(0.868-1.824) | 1.123(0.731-1.725) |
| lag21 | 1.89(1.254-2.848) | 1.904(1.265-2.866) | 1.95(1.306-2.912) | 1.95(1.322-2.877) | 1.901(1.313-2.752) | 1.802(1.278-2.541) | 1.658(1.217-2.259) | 1.478(1.13-1.932) | 1.291(1.032-1.615) | 1.133(0.946-1.357) | 1.025(0.898-1.17) | 0.981(0.917-1.05) | 1.019(0.99-1.049) | 1.144(0.976-1.34) | 1.305(0.986-1.728) | 1.418(0.99-2.03) | 1.384(0.939-2.04) | 1.218(0.775-1.912) |

| Table S22 Risk of 0y DN with each 1 °C change in lag time from 13.8 °C to 30 °C | | | | | | | | | | | | | | | | | | |
| --- | --- | --- | --- | --- | --- | --- | --- | --- | --- | --- | --- | --- | --- | --- | --- | --- | --- | --- |
| Lag(days) | Temperature(℃), RR(95%CI) | | | | | | | | | | | | | | | | | |
|  | 13.8 | 14 | 15 | 16 | 17 | 18 | 19 | 20 | 21 | 22 | 23 | 24 | 25 | 26 | 27 | 28 | 29 | 30 |
| lag0 | 0.863(0.79-0.943) | 0.866(0.794-0.945) | 0.884(0.813-0.96) | 0.901(0.833-0.974) | 0.917(0.852-0.988) | 0.933(0.872-0.999) | 0.949(0.893-1.008) | 0.963(0.915-1.014) | 0.976(0.937-1.017) | 0.988(0.958-1.018) | 0.996(0.976-1.016) | 1(0.991-1.009) | 0.999(0.995-1.003) | 0.994(0.974-1.015) | 0.987(0.95-1.026) | 0.982(0.932-1.034) | 0.98(0.924-1.04) | 0.983(0.92-1.049) |
| lag1 | 0.823(0.752-0.902) | 0.828(0.756-0.906) | 0.849(0.78-0.925) | 0.871(0.803-0.945) | 0.892(0.826-0.963) | 0.912(0.85-0.979) | 0.931(0.875-0.992) | 0.95(0.901-1.002) | 0.967(0.927-1.008) | 0.981(0.951-1.012) | 0.992(0.972-1.013) | 0.999(0.989-1.009) | 1(0.996-1.004) | 0.996(0.974-1.018) | 0.99(0.95-1.031) | 0.986(0.933-1.041) | 0.986(0.927-1.048) | 0.99(0.925-1.06) |
| lag2 | 0.805(0.735-0.881) | 0.809(0.74-0.885) | 0.831(0.763-0.905) | 0.853(0.786-0.926) | 0.875(0.809-0.945) | 0.896(0.834-0.962) | 0.916(0.859-0.977) | 0.936(0.887-0.988) | 0.955(0.915-0.996) | 0.971(0.941-1.002) | 0.985(0.965-1.006) | 0.995(0.985-1.005) | 1.001(0.997-1.005) | 1.002(0.979-1.026) | 1.001(0.96-1.045) | 1.001(0.946-1.059) | 1.002(0.942-1.067) | 1.006(0.941-1.075) |
| lag3 | 0.775(0.702-0.854) | 0.779(0.707-0.858) | 0.8(0.729-0.878) | 0.822(0.752-0.898) | 0.844(0.776-0.918) | 0.866(0.801-0.936) | 0.889(0.829-0.952) | 0.911(0.859-0.966) | 0.933(0.891-0.977) | 0.954(0.922-0.987) | 0.973(0.952-0.996) | 0.99(0.979-1.001) | 1.003(0.998-1.008) | 1.012(0.987-1.038) | 1.017(0.972-1.065) | 1.02(0.96-1.084) | 1.021(0.956-1.09) | 1.02(0.952-1.093) |
| lag4 | 0.737(0.664-0.819) | 0.742(0.669-0.822) | 0.762(0.69-0.842) | 0.784(0.713-0.862) | 0.807(0.737-0.882) | 0.83(0.764-0.902) | 0.855(0.794-0.92) | 0.881(0.827-0.937) | 0.907(0.864-0.953) | 0.934(0.901-0.968) | 0.96(0.937-0.983) | 0.984(0.973-0.995) | 1.005(1-1.01) | 1.023(0.996-1.05) | 1.035(0.987-1.086) | 1.041(0.978-1.108) | 1.04(0.972-1.113) | 1.032(0.962-1.109) |
| lag5 | 0.7(0.626-0.784) | 0.704(0.63-0.787) | 0.724(0.651-0.806) | 0.746(0.673-0.826) | 0.769(0.698-0.847) | 0.794(0.726-0.868) | 0.821(0.758-0.889) | 0.85(0.795-0.909) | 0.881(0.836-0.929) | 0.913(0.879-0.949) | 0.946(0.922-0.97) | 0.978(0.966-0.989) | 1.007(1.002-1.013) | 1.034(1.005-1.063) | 1.053(1.001-1.107) | 1.062(0.994-1.134) | 1.058(0.986-1.136) | 1.044(0.969-1.124) |
| lag6 | 0.666(0.589-0.754) | 0.67(0.593-0.757) | 0.69(0.614-0.775) | 0.711(0.636-0.796) | 0.735(0.661-0.817) | 0.761(0.69-0.839) | 0.79(0.724-0.862) | 0.822(0.764-0.884) | 0.857(0.81-0.908) | 0.895(0.858-0.933) | 0.933(0.908-0.959) | 0.972(0.959-0.985) | 1.01(1.004-1.015) | 1.043(1.013-1.075) | 1.069(1.013-1.128) | 1.081(1.008-1.16) | 1.076(0.998-1.16) | 1.055(0.975-1.142) |
| lag7 | 0.638(0.559-0.729) | 0.642(0.562-0.732) | 0.661(0.582-0.75) | 0.682(0.604-0.771) | 0.707(0.63-0.793) | 0.734(0.66-0.816) | 0.764(0.696-0.84) | 0.799(0.738-0.865) | 0.837(0.787-0.891) | 0.879(0.84-0.919) | 0.922(0.896-0.95) | 0.967(0.954-0.981) | 1.011(1.005-1.017) | 1.052(1.019-1.086) | 1.083(1.023-1.147) | 1.098(1.019-1.184) | 1.092(1.008-1.183) | 1.067(0.981-1.16) |
| lag8 | 0.615(0.533-0.71) | 0.619(0.537-0.713) | 0.638(0.557-0.731) | 0.659(0.579-0.751) | 0.684(0.605-0.773) | 0.712(0.636-0.797) | 0.744(0.672-0.822) | 0.78(0.717-0.849) | 0.821(0.769-0.877) | 0.866(0.826-0.908) | 0.914(0.886-0.943) | 0.963(0.949-0.977) | 1.013(1.007-1.019) | 1.059(1.024-1.095) | 1.095(1.031-1.164) | 1.113(1.029-1.204) | 1.107(1.017-1.204) | 1.078(0.988-1.178) |
| lag9 | 0.597(0.513-0.694) | 0.6(0.517-0.697) | 0.619(0.536-0.715) | 0.641(0.558-0.735) | 0.666(0.585-0.758) | 0.694(0.616-0.783) | 0.727(0.654-0.809) | 0.765(0.7-0.837) | 0.808(0.754-0.867) | 0.856(0.814-0.9) | 0.907(0.878-0.937) | 0.96(0.945-0.975) | 1.014(1.008-1.02) | 1.065(1.029-1.103) | 1.105(1.038-1.177) | 1.126(1.038-1.222) | 1.12(1.027-1.222) | 1.091(0.996-1.195) |
| lag10 | 0.582(0.497-0.683) | 0.586(0.5-0.686) | 0.605(0.52-0.704) | 0.626(0.542-0.724) | 0.651(0.568-0.747) | 0.681(0.6-0.772) | 0.714(0.639-0.799) | 0.753(0.686-0.827) | 0.798(0.742-0.859) | 0.848(0.804-0.893) | 0.901(0.871-0.932) | 0.957(0.942-0.973) | 1.015(1.008-1.022) | 1.07(1.032-1.109) | 1.114(1.044-1.188) | 1.137(1.045-1.237) | 1.133(1.036-1.239) | 1.103(1.004-1.211) |
| lag11 | 0.571(0.483-0.675) | 0.575(0.487-0.678) | 0.593(0.506-0.696) | 0.615(0.528-0.716) | 0.641(0.555-0.74) | 0.67(0.587-0.765) | 0.704(0.626-0.792) | 0.744(0.674-0.821) | 0.79(0.732-0.853) | 0.841(0.796-0.889) | 0.897(0.865-0.929) | 0.955(0.94-0.971) | 1.016(1.009-1.023) | 1.074(1.034-1.114) | 1.121(1.048-1.198) | 1.147(1.051-1.251) | 1.144(1.043-1.254) | 1.115(1.013-1.228) |
| lag12 | 0.563(0.473-0.671) | 0.566(0.476-0.674) | 0.585(0.495-0.691) | 0.607(0.517-0.712) | 0.632(0.544-0.735) | 0.662(0.576-0.761) | 0.697(0.616-0.788) | 0.737(0.665-0.818) | 0.784(0.723-0.85) | 0.836(0.789-0.886) | 0.893(0.861-0.927) | 0.954(0.938-0.97) | 1.016(1.009-1.024) | 1.077(1.036-1.119) | 1.127(1.051-1.208) | 1.155(1.056-1.264) | 1.155(1.05-1.27) | 1.128(1.021-1.246) |
| lag13 | 0.557(0.464-0.67) | 0.561(0.467-0.673) | 0.579(0.486-0.69) | 0.601(0.509-0.711) | 0.627(0.535-0.734) | 0.657(0.568-0.76) | 0.692(0.608-0.787) | 0.733(0.657-0.817) | 0.78(0.717-0.849) | 0.833(0.784-0.885) | 0.891(0.857-0.926) | 0.953(0.936-0.97) | 1.017(1.009-1.024) | 1.079(1.037-1.123) | 1.132(1.053-1.216) | 1.163(1.06-1.277) | 1.165(1.056-1.286) | 1.14(1.028-1.265) |
| lag14 | 0.554(0.457-0.671) | 0.557(0.461-0.674) | 0.576(0.479-0.692) | 0.598(0.502-0.712) | 0.623(0.528-0.736) | 0.653(0.561-0.761) | 0.688(0.601-0.788) | 0.729(0.651-0.817) | 0.777(0.711-0.849) | 0.83(0.779-0.885) | 0.889(0.853-0.926) | 0.952(0.934-0.97) | 1.017(1.009-1.025) | 1.082(1.038-1.128) | 1.136(1.054-1.224) | 1.17(1.062-1.289) | 1.175(1.061-1.301) | 1.153(1.035-1.284) |
| lag15 | 0.552(0.452-0.675) | 0.556(0.456-0.678) | 0.574(0.474-0.695) | 0.596(0.496-0.716) | 0.622(0.523-0.739) | 0.651(0.555-0.764) | 0.686(0.596-0.791) | 0.727(0.646-0.819) | 0.775(0.706-0.85) | 0.829(0.775-0.885) | 0.887(0.851-0.926) | 0.951(0.933-0.97) | 1.018(1.009-1.026) | 1.083(1.038-1.131) | 1.14(1.055-1.232) | 1.177(1.065-1.301) | 1.185(1.066-1.317) | 1.165(1.042-1.303) |
| lag16 | 0.553(0.449-0.68) | 0.556(0.452-0.683) | 0.574(0.471-0.701) | 0.596(0.493-0.721) | 0.621(0.519-0.744) | 0.651(0.551-0.769) | 0.686(0.592-0.795) | 0.727(0.642-0.823) | 0.774(0.703-0.853) | 0.827(0.772-0.887) | 0.887(0.848-0.926) | 0.95(0.931-0.97) | 1.018(1.009-1.026) | 1.085(1.038-1.135) | 1.144(1.056-1.239) | 1.183(1.067-1.312) | 1.194(1.071-1.332) | 1.178(1.049-1.322) |
| lag17 | 0.555(0.447-0.688) | 0.558(0.45-0.691) | 0.576(0.469-0.708) | 0.598(0.49-0.728) | 0.623(0.517-0.751) | 0.652(0.549-0.775) | 0.687(0.589-0.8) | 0.727(0.639-0.827) | 0.774(0.7-0.856) | 0.827(0.77-0.889) | 0.886(0.846-0.927) | 0.95(0.93-0.97) | 1.018(1.009-1.027) | 1.087(1.038-1.138) | 1.148(1.057-1.246) | 1.19(1.07-1.322) | 1.203(1.076-1.346) | 1.19(1.056-1.34) |
| lag18 | 0.558(0.447-0.697) | 0.561(0.45-0.7) | 0.579(0.468-0.717) | 0.601(0.49-0.737) | 0.625(0.515-0.759) | 0.654(0.547-0.782) | 0.688(0.587-0.807) | 0.728(0.637-0.832) | 0.774(0.698-0.859) | 0.827(0.768-0.891) | 0.885(0.845-0.928) | 0.949(0.929-0.97) | 1.018(1.009-1.027) | 1.089(1.039-1.141) | 1.152(1.059-1.252) | 1.196(1.074-1.333) | 1.212(1.081-1.36) | 1.202(1.063-1.358) |
| lag19 | 0.563(0.448-0.709) | 0.567(0.451-0.712) | 0.584(0.469-0.728) | 0.605(0.49-0.747) | 0.629(0.515-0.769) | 0.658(0.547-0.791) | 0.691(0.586-0.815) | 0.73(0.636-0.839) | 0.776(0.696-0.864) | 0.827(0.766-0.894) | 0.885(0.843-0.93) | 0.949(0.928-0.97) | 1.019(1.009-1.028) | 1.09(1.039-1.144) | 1.156(1.06-1.259) | 1.203(1.077-1.344) | 1.222(1.086-1.374) | 1.213(1.069-1.377) |
| lag20 | 0.57(0.449-0.724) | 0.573(0.453-0.726) | 0.59(0.47-0.742) | 0.611(0.49-0.76) | 0.634(0.515-0.781) | 0.662(0.546-0.802) | 0.695(0.585-0.824) | 0.733(0.634-0.846) | 0.777(0.694-0.87) | 0.828(0.764-0.898) | 0.885(0.841-0.932) | 0.949(0.927-0.971) | 1.019(1.009-1.028) | 1.092(1.039-1.148) | 1.16(1.061-1.267) | 1.21(1.079-1.356) | 1.231(1.09-1.39) | 1.225(1.074-1.396) |
| lag21 | 0.579(0.451-0.742) | 0.582(0.454-0.744) | 0.598(0.471-0.759) | 0.618(0.491-0.776) | 0.64(0.516-0.796) | 0.667(0.546-0.816) | 0.699(0.584-0.836) | 0.736(0.633-0.856) | 0.779(0.692-0.877) | 0.829(0.762-0.902) | 0.885(0.839-0.934) | 0.949(0.926-0.972) | 1.019(1.009-1.029) | 1.094(1.039-1.152) | 1.164(1.061-1.277) | 1.217(1.08-1.371) | 1.24(1.092-1.409) | 1.236(1.077-1.418) |

| Table S23 Risk of 1-5y DN with each 1 °C change in lag time from 13.8 °C to 30 °C | | | | | | | | | | | | | | | | | | |
| --- | --- | --- | --- | --- | --- | --- | --- | --- | --- | --- | --- | --- | --- | --- | --- | --- | --- | --- |
| Lag(days) | Temperature(℃), RR(95%CI) | | | | | | | | | | | | | | | | | |
|  | 13.8 | 14 | 15 | 16 | 17 | 18 | 19 | 20 | 21 | 22 | 23 | 24 | 25 | 26 | 27 | 28 | 29 | 30 |
| lag0 | 0.856(0.793-0.924) | 0.859(0.797-0.927) | 0.875(0.814-0.941) | 0.891(0.832-0.955) | 0.907(0.851-0.968) | 0.924(0.871-0.981) | 0.941(0.892-0.992) | 0.958(0.915-1.002) | 0.974(0.939-1.01) | 0.988(0.961-1.016) | 0.998(0.979-1.017) | 1.002(0.993-1.011) | 0.998(0.994-1.002) | 0.987(0.966-1.008) | 0.974(0.937-1.012) | 0.963(0.914-1.014) | 0.96(0.904-1.018) | 0.963(0.9-1.031) |
| lag1 | 0.845(0.78-0.914) | 0.848(0.784-0.917) | 0.867(0.804-0.934) | 0.885(0.824-0.951) | 0.904(0.845-0.967) | 0.922(0.866-0.981) | 0.94(0.889-0.993) | 0.957(0.913-1.003) | 0.973(0.937-1.011) | 0.987(0.959-1.016) | 0.997(0.977-1.017) | 1.001(0.991-1.011) | 0.999(0.995-1.003) | 0.99(0.968-1.012) | 0.977(0.938-1.018) | 0.964(0.913-1.018) | 0.953(0.896-1.014) | 0.945(0.881-1.013) |
| lag2 | 0.87(0.805-0.94) | 0.874(0.809-0.944) | 0.891(0.827-0.96) | 0.908(0.845-0.975) | 0.924(0.863-0.988) | 0.939(0.882-0.999) | 0.953(0.901-1.007) | 0.965(0.92-1.012) | 0.976(0.94-1.014) | 0.985(0.957-1.014) | 0.993(0.973-1.013) | 0.998(0.988-1.008) | 1(0.996-1.005) | 1(0.977-1.024) | 0.996(0.954-1.039) | 0.985(0.932-1.042) | 0.968(0.91-1.03) | 0.945(0.883-1.012) |
| lag3 | 0.885(0.814-0.963) | 0.889(0.818-0.966) | 0.905(0.835-0.98) | 0.92(0.852-0.994) | 0.934(0.868-1.004) | 0.946(0.884-1.012) | 0.956(0.901-1.016) | 0.965(0.917-1.016) | 0.972(0.933-1.013) | 0.978(0.948-1.009) | 0.985(0.964-1.006) | 0.993(0.982-1.003) | 1.003(0.998-1.008) | 1.014(0.989-1.04) | 1.02(0.975-1.068) | 1.016(0.957-1.078) | 0.995(0.932-1.062) | 0.961(0.895-1.031) |
| lag4 | 0.891(0.815-0.973) | 0.894(0.818-0.976) | 0.909(0.835-0.99) | 0.923(0.85-1.001) | 0.934(0.865-1.01) | 0.945(0.879-1.015) | 0.953(0.894-1.016) | 0.959(0.908-1.012) | 0.963(0.922-1.006) | 0.968(0.937-1) | 0.975(0.954-0.998) | 0.987(0.976-0.998) | 1.005(1.001-1.01) | 1.029(1.002-1.056) | 1.047(0.999-1.098) | 1.05(0.987-1.117) | 1.029(0.962-1.1) | 0.985(0.916-1.06) |
| lag5 | 0.891(0.81-0.98) | 0.894(0.813-0.982) | 0.907(0.828-0.994) | 0.92(0.842-1.004) | 0.93(0.856-1.011) | 0.939(0.869-1.014) | 0.945(0.882-1.012) | 0.949(0.896-1.006) | 0.952(0.909-0.997) | 0.956(0.924-0.99) | 0.965(0.943-0.989) | 0.981(0.97-0.993) | 1.008(1.003-1.013) | 1.043(1.015-1.073) | 1.075(1.022-1.13) | 1.086(1.018-1.159) | 1.065(0.993-1.142) | 1.015(0.941-1.094) |
| lag6 | 0.889(0.802-0.986) | 0.892(0.805-0.988) | 0.904(0.819-0.999) | 0.915(0.832-1.007) | 0.924(0.844-1.012) | 0.931(0.856-1.012) | 0.936(0.869-1.008) | 0.939(0.881-1) | 0.941(0.895-0.989) | 0.945(0.91-0.981) | 0.956(0.931-0.98) | 0.976(0.964-0.988) | 1.011(1.005-1.016) | 1.057(1.027-1.089) | 1.101(1.044-1.161) | 1.121(1.046-1.202) | 1.101(1.022-1.187) | 1.045(0.965-1.132) |
| lag7 | 0.888(0.794-0.993) | 0.891(0.797-0.995) | 0.901(0.809-1.003) | 0.91(0.821-1.01) | 0.918(0.832-1.012) | 0.924(0.844-1.011) | 0.927(0.856-1.005) | 0.929(0.868-0.994) | 0.93(0.881-0.981) | 0.935(0.898-0.973) | 0.947(0.921-0.973) | 0.971(0.958-0.984) | 1.013(1.007-1.019) | 1.07(1.037-1.104) | 1.125(1.062-1.191) | 1.153(1.071-1.242) | 1.135(1.048-1.229) | 1.074(0.986-1.169) |
| lag8 | 0.889(0.789-1.001) | 0.891(0.791-1.003) | 0.9(0.802-1.009) | 0.907(0.812-1.013) | 0.913(0.822-1.014) | 0.917(0.833-1.01) | 0.92(0.844-1.002) | 0.92(0.856-0.989) | 0.92(0.869-0.975) | 0.925(0.887-0.966) | 0.939(0.912-0.967) | 0.967(0.953-0.981) | 1.015(1.008-1.021) | 1.081(1.046-1.117) | 1.146(1.079-1.216) | 1.182(1.094-1.277) | 1.165(1.073-1.266) | 1.101(1.007-1.203) |
| lag9 | 0.891(0.785-1.011) | 0.892(0.787-1.011) | 0.899(0.797-1.015) | 0.905(0.805-1.017) | 0.909(0.814-1.015) | 0.912(0.824-1.01) | 0.913(0.834-1) | 0.913(0.845-0.985) | 0.913(0.859-0.97) | 0.918(0.877-0.96) | 0.933(0.905-0.962) | 0.963(0.949-0.978) | 1.016(1.01-1.023) | 1.09(1.053-1.129) | 1.164(1.094-1.238) | 1.207(1.114-1.308) | 1.192(1.095-1.299) | 1.124(1.026-1.232) |
| lag10 | 0.895(0.784-1.022) | 0.896(0.785-1.022) | 0.901(0.793-1.023) | 0.904(0.8-1.022) | 0.907(0.807-1.019) | 0.908(0.816-1.011) | 0.908(0.825-0.999) | 0.907(0.836-0.982) | 0.906(0.85-0.965) | 0.911(0.869-0.955) | 0.927(0.898-0.957) | 0.96(0.946-0.975) | 1.018(1.011-1.024) | 1.098(1.06-1.138) | 1.179(1.106-1.257) | 1.229(1.131-1.334) | 1.216(1.113-1.327) | 1.146(1.043-1.259) |
| lag11 | 0.9(0.784-1.035) | 0.901(0.785-1.035) | 0.904(0.79-1.033) | 0.905(0.796-1.03) | 0.906(0.802-1.024) | 0.906(0.809-1.014) | 0.905(0.818-1) | 0.902(0.829-0.981) | 0.901(0.843-0.963) | 0.906(0.863-0.952) | 0.923(0.893-0.954) | 0.958(0.943-0.974) | 1.019(1.012-1.026) | 1.104(1.065-1.146) | 1.192(1.116-1.273) | 1.246(1.145-1.357) | 1.236(1.129-1.352) | 1.164(1.057-1.283) |
| lag12 | 0.908(0.785-1.051) | 0.908(0.786-1.05) | 0.909(0.79-1.046) | 0.908(0.794-1.04) | 0.907(0.798-1.031) | 0.905(0.805-1.019) | 0.903(0.813-1.003) | 0.899(0.823-0.982) | 0.897(0.837-0.962) | 0.903(0.857-0.95) | 0.92(0.889-0.952) | 0.956(0.941-0.972) | 1.02(1.012-1.027) | 1.109(1.068-1.152) | 1.202(1.122-1.287) | 1.261(1.155-1.376) | 1.252(1.141-1.374) | 1.18(1.068-1.304) |
| lag13 | 0.918(0.788-1.069) | 0.917(0.788-1.068) | 0.916(0.79-1.061) | 0.913(0.793-1.052) | 0.91(0.796-1.041) | 0.907(0.801-1.026) | 0.903(0.809-1.008) | 0.898(0.819-0.985) | 0.896(0.832-0.964) | 0.9(0.853-0.95) | 0.918(0.886-0.952) | 0.955(0.939-0.972) | 1.02(1.013-1.028) | 1.113(1.07-1.158) | 1.209(1.126-1.298) | 1.271(1.161-1.393) | 1.265(1.149-1.393) | 1.194(1.076-1.324) |
| lag14 | 0.93(0.792-1.091) | 0.929(0.792-1.089) | 0.925(0.793-1.078) | 0.92(0.793-1.067) | 0.915(0.795-1.053) | 0.91(0.799-1.036) | 0.905(0.806-1.015) | 0.899(0.816-0.99) | 0.895(0.829-0.967) | 0.899(0.85-0.952) | 0.917(0.883-0.952) | 0.954(0.937-0.972) | 1.02(1.013-1.028) | 1.115(1.07-1.162) | 1.213(1.127-1.306) | 1.279(1.164-1.405) | 1.274(1.153-1.408) | 1.204(1.082-1.341) |
| lag15 | 0.944(0.799-1.115) | 0.942(0.798-1.112) | 0.936(0.797-1.099) | 0.929(0.796-1.085) | 0.922(0.796-1.068) | 0.915(0.799-1.048) | 0.908(0.805-1.025) | 0.901(0.814-0.998) | 0.897(0.828-0.971) | 0.9(0.849-0.955) | 0.917(0.882-0.954) | 0.954(0.937-0.972) | 1.021(1.013-1.029) | 1.116(1.069-1.164) | 1.216(1.126-1.312) | 1.283(1.163-1.414) | 1.281(1.155-1.42) | 1.213(1.085-1.355) |
| lag16 | 0.96(0.807-1.142) | 0.958(0.807-1.139) | 0.949(0.803-1.122) | 0.941(0.801-1.105) | 0.932(0.8-1.086) | 0.923(0.802-1.063) | 0.914(0.806-1.037) | 0.906(0.815-1.007) | 0.9(0.828-0.978) | 0.902(0.849-0.959) | 0.918(0.882-0.956) | 0.955(0.937-0.973) | 1.02(1.012-1.029) | 1.115(1.067-1.165) | 1.215(1.124-1.314) | 1.284(1.161-1.42) | 1.283(1.154-1.428) | 1.218(1.086-1.367) |
| lag17 | 0.979(0.818-1.172) | 0.977(0.817-1.168) | 0.966(0.812-1.149) | 0.955(0.808-1.128) | 0.944(0.805-1.106) | 0.933(0.806-1.08) | 0.923(0.81-1.051) | 0.912(0.817-1.018) | 0.905(0.83-0.987) | 0.906(0.85-0.965) | 0.92(0.883-0.96) | 0.956(0.937-0.975) | 1.02(1.012-1.029) | 1.114(1.065-1.165) | 1.213(1.119-1.314) | 1.281(1.156-1.421) | 1.283(1.15-1.431) | 1.221(1.085-1.375) |
| lag18 | 1.001(0.831-1.206) | 0.998(0.83-1.201) | 0.985(0.823-1.178) | 0.971(0.817-1.155) | 0.958(0.813-1.13) | 0.946(0.812-1.101) | 0.933(0.815-1.068) | 0.921(0.822-1.032) | 0.912(0.833-0.997) | 0.911(0.853-0.973) | 0.924(0.885-0.965) | 0.957(0.938-0.977) | 1.019(1.011-1.028) | 1.111(1.061-1.163) | 1.208(1.112-1.311) | 1.276(1.148-1.418) | 1.28(1.145-1.431) | 1.222(1.081-1.381) |
| lag19 | 1.025(0.846-1.243) | 1.022(0.844-1.238) | 1.007(0.836-1.212) | 0.991(0.828-1.186) | 0.976(0.823-1.157) | 0.961(0.821-1.125) | 0.946(0.822-1.089) | 0.932(0.828-1.049) | 0.92(0.838-1.01) | 0.917(0.857-0.982) | 0.928(0.888-0.971) | 0.959(0.939-0.98) | 1.019(1.01-1.028) | 1.107(1.056-1.16) | 1.201(1.104-1.306) | 1.268(1.138-1.412) | 1.274(1.136-1.428) | 1.22(1.076-1.384) |
| lag20 | 1.053(0.863-1.286) | 1.05(0.861-1.28) | 1.032(0.851-1.251) | 1.014(0.842-1.221) | 0.996(0.835-1.189) | 0.979(0.831-1.153) | 0.962(0.831-1.113) | 0.945(0.836-1.069) | 0.931(0.845-1.026) | 0.926(0.862-0.994) | 0.934(0.891-0.978) | 0.962(0.941-0.983) | 1.018(1.008-1.027) | 1.101(1.049-1.156) | 1.192(1.093-1.3) | 1.257(1.124-1.404) | 1.264(1.123-1.423) | 1.216(1.067-1.386) |
| lag21 | 1.084(0.88-1.335) | 1.08(0.878-1.329) | 1.06(0.867-1.296) | 1.04(0.856-1.263) | 1.02(0.848-1.227) | 1(0.843-1.187) | 0.98(0.841-1.142) | 0.961(0.844-1.093) | 0.944(0.853-1.045) | 0.935(0.868-1.008) | 0.94(0.895-0.988) | 0.965(0.943-0.987) | 1.016(1.007-1.026) | 1.095(1.041-1.152) | 1.18(1.078-1.293) | 1.243(1.106-1.396) | 1.252(1.106-1.418) | 1.21(1.054-1.389) |

| Table S24 Risk of 6-20y DN with each 1 °C change in lag time from 13.8 °C to 30 °C | | | | | | | | | | | | | | | | | | |
| --- | --- | --- | --- | --- | --- | --- | --- | --- | --- | --- | --- | --- | --- | --- | --- | --- | --- | --- |
| Lag(days) | Temperature(℃), RR(95%CI) | | | | | | | | | | | | | | | | | |
|  | 13.8 | 14 | 15 | 16 | 17 | 18 | 19 | 20 | 21 | 22 | 23 | 24 | 25 | 26 | 27 | 28 | 29 | 30 |
| lag0 | 0.839(0.749-0.94) | 0.841(0.752-0.942) | 0.853(0.766-0.95) | 0.867(0.782-0.96) | 0.882(0.801-0.972) | 0.901(0.824-0.984) | 0.922(0.852-0.997) | 0.946(0.884-1.011) | 0.971(0.92-1.024) | 0.993(0.953-1.034) | 1.007(0.98-1.036) | 1.009(0.996-1.023) | 0.994(0.988-1) | 0.965(0.934-0.996) | 0.934(0.881-0.99) | 0.916(0.847-0.991) | 0.922(0.842-1.01) | 0.951(0.855-1.057) |
| lag1 | 0.807(0.717-0.908) | 0.81(0.72-0.91) | 0.824(0.737-0.921) | 0.84(0.755-0.934) | 0.858(0.776-0.948) | 0.879(0.801-0.963) | 0.902(0.831-0.979) | 0.928(0.866-0.995) | 0.956(0.904-1.011) | 0.981(0.94-1.023) | 0.999(0.97-1.028) | 1.005(0.991-1.02) | 0.996(0.99-1.002) | 0.972(0.94-1.005) | 0.944(0.888-1.003) | 0.923(0.851-1.002) | 0.919(0.837-1.009) | 0.929(0.834-1.036) |
| lag2 | 0.825(0.734-0.927) | 0.828(0.737-0.929) | 0.84(0.752-0.939) | 0.854(0.768-0.95) | 0.87(0.787-0.961) | 0.887(0.808-0.973) | 0.906(0.834-0.984) | 0.927(0.864-0.994) | 0.949(0.898-1.004) | 0.97(0.93-1.012) | 0.987(0.959-1.017) | 0.998(0.983-1.013) | 0.999(0.993-1.006) | 0.991(0.957-1.026) | 0.976(0.917-1.04) | 0.959(0.882-1.042) | 0.943(0.859-1.035) | 0.927(0.835-1.03) |
| lag3 | 0.854(0.754-0.968) | 0.856(0.756-0.969) | 0.865(0.768-0.974) | 0.875(0.781-0.981) | 0.887(0.796-0.988) | 0.9(0.814-0.994) | 0.914(0.836-0.999) | 0.93(0.863-1.002) | 0.947(0.892-1.006) | 0.964(0.921-1.009) | 0.98(0.949-1.011) | 0.993(0.977-1.009) | 1.002(0.995-1.009) | 1.005(0.968-1.044) | 1.002(0.936-1.072) | 0.989(0.905-1.082) | 0.968(0.877-1.068) | 0.938(0.841-1.047) |
| lag4 | 0.885(0.776-1.01) | 0.886(0.777-1.01) | 0.891(0.785-1.011) | 0.897(0.795-1.012) | 0.904(0.807-1.013) | 0.912(0.821-1.014) | 0.922(0.84-1.013) | 0.934(0.863-1.011) | 0.947(0.889-1.009) | 0.961(0.916-1.008) | 0.975(0.943-1.008) | 0.99(0.973-1.006) | 1.004(0.996-1.011) | 1.015(0.976-1.055) | 1.02(0.95-1.094) | 1.013(0.923-1.111) | 0.991(0.896-1.096) | 0.957(0.856-1.069) |
| lag5 | 0.914(0.793-1.052) | 0.914(0.794-1.051) | 0.914(0.799-1.046) | 0.916(0.805-1.042) | 0.919(0.813-1.039) | 0.924(0.825-1.034) | 0.93(0.841-1.028) | 0.938(0.862-1.02) | 0.947(0.886-1.013) | 0.959(0.912-1.009) | 0.972(0.939-1.007) | 0.987(0.97-1.005) | 1.005(0.997-1.012) | 1.022(0.981-1.065) | 1.033(0.959-1.112) | 1.032(0.936-1.136) | 1.013(0.912-1.124) | 0.979(0.873-1.097) |
| lag6 | 0.936(0.803-1.091) | 0.935(0.803-1.089) | 0.932(0.805-1.079) | 0.931(0.809-1.071) | 0.93(0.815-1.062) | 0.932(0.825-1.053) | 0.935(0.839-1.042) | 0.94(0.858-1.03) | 0.948(0.881-1.019) | 0.957(0.907-1.011) | 0.97(0.934-1.007) | 0.986(0.968-1.004) | 1.005(0.997-1.014) | 1.027(0.983-1.073) | 1.043(0.964-1.129) | 1.047(0.945-1.161) | 1.032(0.924-1.153) | 1.001(0.887-1.13) |
| lag7 | 0.95(0.805-1.122) | 0.949(0.805-1.119) | 0.944(0.805-1.106) | 0.94(0.807-1.094) | 0.937(0.812-1.082) | 0.937(0.82-1.069) | 0.938(0.834-1.055) | 0.941(0.852-1.039) | 0.947(0.875-1.024) | 0.956(0.901-1.013) | 0.968(0.93-1.007) | 0.984(0.965-1.004) | 1.006(0.998-1.015) | 1.031(0.984-1.08) | 1.052(0.967-1.144) | 1.061(0.951-1.183) | 1.05(0.934-1.181) | 1.022(0.899-1.161) |
| lag8 | 0.957(0.801-1.142) | 0.955(0.801-1.139) | 0.948(0.8-1.124) | 0.943(0.801-1.11) | 0.94(0.806-1.096) | 0.938(0.814-1.08) | 0.938(0.827-1.064) | 0.94(0.846-1.045) | 0.945(0.869-1.028) | 0.953(0.896-1.015) | 0.966(0.926-1.008) | 0.983(0.963-1.004) | 1.007(0.998-1.016) | 1.035(0.985-1.087) | 1.059(0.97-1.157) | 1.072(0.957-1.202) | 1.065(0.943-1.203) | 1.04(0.911-1.188) |
| lag9 | 0.957(0.793-1.154) | 0.955(0.793-1.151) | 0.948(0.792-1.135) | 0.942(0.793-1.12) | 0.938(0.797-1.104) | 0.936(0.806-1.088) | 0.936(0.819-1.069) | 0.938(0.839-1.049) | 0.943(0.863-1.03) | 0.951(0.891-1.015) | 0.964(0.922-1.007) | 0.982(0.961-1.004) | 1.007(0.998-1.017) | 1.038(0.986-1.092) | 1.065(0.972-1.167) | 1.081(0.961-1.217) | 1.078(0.951-1.222) | 1.056(0.921-1.21) |
| lag10 | 0.951(0.781-1.159) | 0.95(0.781-1.156) | 0.943(0.78-1.14) | 0.938(0.782-1.125) | 0.934(0.787-1.109) | 0.932(0.796-1.092) | 0.933(0.81-1.073) | 0.935(0.831-1.052) | 0.94(0.857-1.031) | 0.949(0.886-1.016) | 0.962(0.919-1.007) | 0.981(0.959-1.004) | 1.008(0.998-1.017) | 1.04(0.987-1.096) | 1.07(0.974-1.175) | 1.088(0.964-1.228) | 1.088(0.956-1.237) | 1.069(0.929-1.229) |
| lag11 | 0.943(0.766-1.16) | 0.941(0.766-1.157) | 0.935(0.766-1.142) | 0.93(0.768-1.127) | 0.927(0.774-1.111) | 0.927(0.784-1.094) | 0.928(0.8-1.075) | 0.931(0.822-1.054) | 0.937(0.85-1.033) | 0.946(0.881-1.017) | 0.96(0.915-1.008) | 0.981(0.958-1.004) | 1.008(0.998-1.018) | 1.041(0.986-1.099) | 1.072(0.973-1.181) | 1.093(0.965-1.238) | 1.094(0.959-1.248) | 1.078(0.934-1.244) |
| lag12 | 0.932(0.749-1.159) | 0.93(0.749-1.156) | 0.925(0.75-1.141) | 0.921(0.753-1.127) | 0.919(0.76-1.112) | 0.919(0.772-1.095) | 0.922(0.789-1.076) | 0.926(0.813-1.055) | 0.933(0.842-1.034) | 0.944(0.876-1.018) | 0.959(0.912-1.009) | 0.98(0.957-1.004) | 1.008(0.998-1.018) | 1.041(0.984-1.101) | 1.073(0.97-1.186) | 1.094(0.962-1.244) | 1.098(0.958-1.257) | 1.085(0.936-1.257) |
| lag13 | 0.919(0.731-1.156) | 0.918(0.731-1.153) | 0.914(0.733-1.139) | 0.911(0.737-1.126) | 0.91(0.745-1.111) | 0.911(0.758-1.096) | 0.915(0.777-1.077) | 0.921(0.803-1.056) | 0.93(0.835-1.035) | 0.942(0.871-1.019) | 0.958(0.91-1.01) | 0.98(0.956-1.005) | 1.008(0.997-1.019) | 1.04(0.982-1.103) | 1.071(0.965-1.189) | 1.093(0.956-1.248) | 1.098(0.954-1.264) | 1.088(0.934-1.267) |
| lag14 | 0.907(0.714-1.153) | 0.906(0.714-1.15) | 0.902(0.716-1.137) | 0.9(0.721-1.124) | 0.9(0.73-1.11) | 0.903(0.744-1.095) | 0.908(0.765-1.077) | 0.915(0.793-1.057) | 0.926(0.827-1.036) | 0.94(0.866-1.021) | 0.958(0.907-1.012) | 0.98(0.955-1.006) | 1.008(0.996-1.019) | 1.038(0.978-1.103) | 1.068(0.958-1.189) | 1.088(0.948-1.249) | 1.095(0.947-1.266) | 1.088(0.929-1.275) |
| lag15 | 0.896(0.697-1.151) | 0.895(0.697-1.148) | 0.891(0.7-1.134) | 0.889(0.705-1.122) | 0.89(0.715-1.109) | 0.894(0.73-1.094) | 0.9(0.753-1.077) | 0.91(0.783-1.057) | 0.923(0.82-1.038) | 0.939(0.861-1.023) | 0.958(0.905-1.014) | 0.981(0.955-1.008) | 1.007(0.996-1.019) | 1.035(0.973-1.102) | 1.062(0.95-1.187) | 1.081(0.937-1.247) | 1.088(0.936-1.265) | 1.085(0.921-1.278) |
| lag16 | 0.885(0.682-1.15) | 0.884(0.682-1.146) | 0.88(0.685-1.132) | 0.879(0.69-1.119) | 0.88(0.7-1.107) | 0.885(0.717-1.092) | 0.893(0.741-1.076) | 0.904(0.773-1.058) | 0.92(0.813-1.04) | 0.938(0.858-1.026) | 0.959(0.904-1.017) | 0.982(0.955-1.01) | 1.007(0.995-1.019) | 1.031(0.967-1.1) | 1.054(0.939-1.182) | 1.07(0.924-1.24) | 1.078(0.924-1.259) | 1.079(0.91-1.278) |
| lag17 | 0.877(0.669-1.15) | 0.876(0.669-1.147) | 0.871(0.671-1.131) | 0.869(0.676-1.117) | 0.871(0.687-1.104) | 0.876(0.704-1.091) | 0.886(0.729-1.075) | 0.899(0.764-1.058) | 0.917(0.807-1.041) | 0.938(0.854-1.029) | 0.96(0.903-1.02) | 0.983(0.955-1.012) | 1.006(0.993-1.018) | 1.026(0.96-1.096) | 1.043(0.927-1.174) | 1.056(0.909-1.228) | 1.065(0.909-1.248) | 1.069(0.898-1.273) |
| lag18 | 0.871(0.658-1.153) | 0.87(0.658-1.149) | 0.863(0.659-1.132) | 0.861(0.664-1.117) | 0.862(0.674-1.103) | 0.868(0.692-1.089) | 0.878(0.718-1.074) | 0.894(0.755-1.058) | 0.914(0.801-1.044) | 0.938(0.852-1.032) | 0.962(0.903-1.025) | 0.985(0.956-1.015) | 1.005(0.992-1.017) | 1.019(0.953-1.091) | 1.031(0.914-1.163) | 1.04(0.891-1.213) | 1.048(0.891-1.233) | 1.057(0.883-1.264) |
| lag19 | 0.868(0.649-1.16) | 0.866(0.649-1.156) | 0.857(0.648-1.135) | 0.853(0.652-1.117) | 0.854(0.662-1.102) | 0.86(0.68-1.088) | 0.872(0.708-1.074) | 0.889(0.747-1.059) | 0.913(0.796-1.046) | 0.939(0.85-1.037) | 0.965(0.904-1.029) | 0.987(0.957-1.018) | 1.003(0.991-1.016) | 1.012(0.944-1.084) | 1.016(0.898-1.149) | 1.02(0.871-1.194) | 1.028(0.871-1.214) | 1.041(0.865-1.252) |
| lag20 | 0.868(0.642-1.172) | 0.865(0.641-1.167) | 0.853(0.638-1.141) | 0.847(0.641-1.121) | 0.847(0.65-1.104) | 0.853(0.668-1.088) | 0.865(0.697-1.074) | 0.885(0.738-1.061) | 0.911(0.79-1.051) | 0.94(0.848-1.043) | 0.968(0.904-1.035) | 0.99(0.959-1.022) | 1.002(0.989-1.015) | 1.003(0.933-1.077) | 0.999(0.879-1.134) | 0.998(0.848-1.173) | 1.005(0.847-1.194) | 1.023(0.844-1.239) |
| lag21 | 0.87(0.636-1.191) | 0.866(0.634-1.184) | 0.851(0.629-1.153) | 0.843(0.629-1.128) | 0.841(0.638-1.108) | 0.846(0.656-1.092) | 0.859(0.685-1.078) | 0.881(0.729-1.066) | 0.91(0.784-1.057) | 0.942(0.845-1.05) | 0.972(0.905-1.043) | 0.993(0.96-1.027) | 1(0.986-1.014) | 0.993(0.921-1.07) | 0.98(0.858-1.12) | 0.973(0.821-1.153) | 0.98(0.818-1.173) | 1.002(0.818-1.226) |

| Table S25 Risk of >20y DN with each 1 °C change in lag time from 13.8 °C to 30 °C | | | | | | | | | | | | | | | | | | |
| --- | --- | --- | --- | --- | --- | --- | --- | --- | --- | --- | --- | --- | --- | --- | --- | --- | --- | --- |
| Lag(days) | Temperature(℃), RR(95%CI) | | | | | | | | | | | | | | | | | |
|  | 13.8 | 14 | 15 | 16 | 17 | 18 | 19 | 20 | 21 | 22 | 23 | 24 | 25 | 26 | 27 | 28 | 29 | 30 |
| lag0 | 0.892(0.823-0.966) | 0.896(0.828-0.97) | 0.92(0.853-0.992) | 0.941(0.876-1.011) | 0.961(0.898-1.027) | 0.977(0.919-1.04) | 0.991(0.938-1.047) | 1.002(0.956-1.05) | 1.009(0.971-1.047) | 1.012(0.983-1.041) | 1.011(0.991-1.031) | 1.006(0.997-1.016) | 0.997(0.993-1.001) | 0.986(0.965-1.008) | 0.978(0.939-1.018) | 0.976(0.925-1.031) | 0.987(0.927-1.05) | 1.008(0.939-1.083) |
| lag1 | 0.862(0.793-0.937) | 0.867(0.799-0.942) | 0.893(0.826-0.966) | 0.917(0.851-0.988) | 0.939(0.876-1.007) | 0.959(0.899-1.022) | 0.975(0.921-1.033) | 0.989(0.942-1.038) | 0.999(0.961-1.039) | 1.005(0.976-1.035) | 1.007(0.987-1.028) | 1.005(0.995-1.015) | 0.998(0.994-1.002) | 0.988(0.965-1.011) | 0.98(0.94-1.022) | 0.979(0.926-1.036) | 0.99(0.928-1.055) | 1.011(0.94-1.088) |
| lag2 | 0.857(0.79-0.931) | 0.862(0.794-0.935) | 0.884(0.817-0.956) | 0.905(0.84-0.975) | 0.924(0.862-0.992) | 0.942(0.883-1.006) | 0.959(0.905-1.016) | 0.973(0.926-1.022) | 0.985(0.947-1.024) | 0.993(0.964-1.023) | 0.999(0.979-1.02) | 1.001(0.991-1.011) | 0.999(0.995-1.003) | 0.994(0.971-1.018) | 0.991(0.948-1.035) | 0.992(0.937-1.051) | 1.002(0.94-1.069) | 1.021(0.951-1.097) |
| lag3 | 0.852(0.78-0.93) | 0.855(0.784-0.934) | 0.875(0.804-0.951) | 0.893(0.824-0.968) | 0.911(0.844-0.983) | 0.928(0.865-0.996) | 0.944(0.887-1.005) | 0.959(0.909-1.011) | 0.972(0.932-1.014) | 0.984(0.953-1.016) | 0.993(0.971-1.015) | 0.998(0.987-1.009) | 1(0.995-1.005) | 0.999(0.974-1.026) | 1(0.954-1.048) | 1.005(0.945-1.069) | 1.02(0.953-1.091) | 1.042(0.968-1.122) |
| lag4 | 0.845(0.769-0.928) | 0.848(0.773-0.931) | 0.866(0.792-0.946) | 0.883(0.811-0.962) | 0.9(0.83-0.975) | 0.916(0.85-0.987) | 0.932(0.873-0.996) | 0.948(0.896-1.003) | 0.963(0.921-1.007) | 0.976(0.944-1.01) | 0.988(0.965-1.011) | 0.996(0.985-1.008) | 1.001(0.996-1.006) | 1.003(0.976-1.03) | 1.007(0.959-1.057) | 1.017(0.954-1.084) | 1.038(0.968-1.112) | 1.068(0.991-1.152) |
| lag5 | 0.84(0.76-0.928) | 0.843(0.763-0.931) | 0.859(0.78-0.945) | 0.875(0.798-0.958) | 0.891(0.817-0.971) | 0.907(0.837-0.982) | 0.923(0.86-0.991) | 0.94(0.885-0.998) | 0.956(0.911-1.002) | 0.971(0.937-1.006) | 0.984(0.961-1.008) | 0.995(0.983-1.007) | 1.001(0.996-1.007) | 1.006(0.978-1.034) | 1.012(0.962-1.065) | 1.027(0.96-1.097) | 1.054(0.981-1.132) | 1.094(1.012-1.182) |
| lag6 | 0.837(0.751-0.933) | 0.84(0.754-0.935) | 0.854(0.77-0.947) | 0.869(0.787-0.96) | 0.884(0.805-0.971) | 0.9(0.825-0.981) | 0.916(0.848-0.99) | 0.933(0.875-0.996) | 0.95(0.903-1.001) | 0.967(0.931-1.005) | 0.982(0.956-1.008) | 0.994(0.981-1.006) | 1.002(0.996-1.007) | 1.007(0.977-1.038) | 1.016(0.962-1.073) | 1.033(0.963-1.109) | 1.066(0.988-1.151) | 1.115(1.027-1.211) |
| lag7 | 0.838(0.745-0.942) | 0.84(0.748-0.944) | 0.853(0.762-0.954) | 0.866(0.778-0.964) | 0.88(0.795-0.974) | 0.895(0.815-0.983) | 0.911(0.839-0.99) | 0.929(0.866-0.996) | 0.946(0.896-1) | 0.964(0.925-1.004) | 0.98(0.953-1.008) | 0.993(0.979-1.007) | 1.002(0.996-1.008) | 1.008(0.976-1.041) | 1.017(0.96-1.078) | 1.037(0.962-1.119) | 1.075(0.991-1.165) | 1.13(1.036-1.232) |
| lag8 | 0.841(0.743-0.953) | 0.843(0.745-0.954) | 0.854(0.758-0.962) | 0.865(0.771-0.97) | 0.878(0.788-0.978) | 0.892(0.807-0.986) | 0.908(0.831-0.992) | 0.925(0.858-0.997) | 0.943(0.89-1.001) | 0.962(0.921-1.005) | 0.979(0.95-1.008) | 0.993(0.978-1.007) | 1.002(0.996-1.008) | 1.008(0.975-1.043) | 1.018(0.958-1.082) | 1.039(0.96-1.124) | 1.078(0.991-1.173) | 1.137(1.039-1.245) |
| lag9 | 0.846(0.742-0.966) | 0.848(0.744-0.966) | 0.856(0.754-0.971) | 0.866(0.767-0.977) | 0.877(0.782-0.984) | 0.89(0.801-0.989) | 0.905(0.824-0.994) | 0.922(0.852-0.998) | 0.941(0.884-1.002) | 0.96(0.917-1.005) | 0.978(0.948-1.009) | 0.992(0.978-1.007) | 1.002(0.995-1.008) | 1.008(0.973-1.044) | 1.017(0.955-1.084) | 1.038(0.957-1.127) | 1.079(0.989-1.176) | 1.139(1.038-1.25) |
| lag10 | 0.852(0.742-0.979) | 0.853(0.744-0.979) | 0.859(0.752-0.981) | 0.867(0.763-0.985) | 0.876(0.777-0.989) | 0.888(0.795-0.993) | 0.903(0.818-0.996) | 0.92(0.847-0.999) | 0.939(0.88-1.003) | 0.959(0.914-1.006) | 0.977(0.946-1.009) | 0.992(0.977-1.008) | 1.002(0.995-1.009) | 1.007(0.971-1.045) | 1.016(0.951-1.084) | 1.036(0.953-1.127) | 1.076(0.985-1.176) | 1.137(1.034-1.251) |
| lag11 | 0.859(0.742-0.993) | 0.859(0.744-0.992) | 0.862(0.75-0.991) | 0.868(0.759-0.992) | 0.876(0.772-0.994) | 0.887(0.789-0.996) | 0.9(0.812-0.999) | 0.918(0.841-1.001) | 0.937(0.875-1.004) | 0.958(0.911-1.007) | 0.977(0.945-1.01) | 0.992(0.976-1.009) | 1.002(0.995-1.009) | 1.006(0.969-1.045) | 1.014(0.947-1.084) | 1.033(0.947-1.126) | 1.072(0.979-1.174) | 1.132(1.027-1.249) |
| lag12 | 0.864(0.742-1.006) | 0.864(0.743-1.005) | 0.865(0.747-1.001) | 0.868(0.754-0.999) | 0.875(0.766-0.999) | 0.884(0.782-1) | 0.898(0.805-1.001) | 0.915(0.835-1.002) | 0.935(0.87-1.005) | 0.957(0.908-1.008) | 0.977(0.943-1.011) | 0.992(0.976-1.009) | 1.002(0.994-1.009) | 1.005(0.966-1.045) | 1.011(0.943-1.084) | 1.028(0.94-1.125) | 1.067(0.971-1.172) | 1.126(1.018-1.245) |
| lag13 | 0.868(0.74-1.018) | 0.867(0.74-1.016) | 0.866(0.742-1.009) | 0.867(0.748-1.005) | 0.872(0.759-1.003) | 0.881(0.775-1.002) | 0.894(0.798-1.002) | 0.912(0.828-1.003) | 0.933(0.865-1.006) | 0.955(0.904-1.009) | 0.976(0.941-1.013) | 0.993(0.975-1.01) | 1.001(0.994-1.009) | 1.004(0.964-1.045) | 1.008(0.937-1.084) | 1.024(0.933-1.123) | 1.06(0.962-1.169) | 1.119(1.008-1.242) |
| lag14 | 0.869(0.736-1.027) | 0.868(0.736-1.025) | 0.864(0.736-1.015) | 0.864(0.741-1.009) | 0.868(0.75-1.005) | 0.876(0.766-1.003) | 0.889(0.789-1.002) | 0.907(0.821-1.003) | 0.93(0.859-1.006) | 0.953(0.9-1.01) | 0.976(0.939-1.014) | 0.993(0.975-1.011) | 1.001(0.993-1.009) | 1.002(0.961-1.045) | 1.004(0.932-1.083) | 1.019(0.925-1.122) | 1.054(0.953-1.166) | 1.112(0.998-1.24) |
| lag15 | 0.868(0.73-1.033) | 0.867(0.729-1.03) | 0.861(0.728-1.018) | 0.859(0.731-1.01) | 0.862(0.74-1.005) | 0.87(0.756-1.002) | 0.883(0.779-1.001) | 0.902(0.812-1.002) | 0.926(0.853-1.005) | 0.951(0.896-1.01) | 0.975(0.937-1.014) | 0.993(0.974-1.012) | 1.001(0.993-1.009) | 1(0.958-1.045) | 1.001(0.926-1.082) | 1.014(0.918-1.12) | 1.049(0.945-1.164) | 1.107(0.989-1.239) |
| lag16 | 0.864(0.721-1.035) | 0.862(0.72-1.032) | 0.855(0.718-1.018) | 0.852(0.72-1.008) | 0.854(0.729-1.001) | 0.862(0.744-0.998) | 0.875(0.769-0.997) | 0.895(0.803-0.999) | 0.921(0.845-1.003) | 0.948(0.891-1.01) | 0.974(0.934-1.015) | 0.993(0.973-1.012) | 1.001(0.993-1.009) | 0.999(0.955-1.045) | 0.998(0.92-1.081) | 1.009(0.91-1.118) | 1.044(0.937-1.162) | 1.104(0.982-1.24) |
| lag17 | 0.856(0.71-1.033) | 0.854(0.709-1.029) | 0.845(0.706-1.013) | 0.842(0.707-1.002) | 0.844(0.716-0.995) | 0.852(0.731-0.991) | 0.866(0.757-0.991) | 0.888(0.792-0.994) | 0.915(0.837-1) | 0.945(0.885-1.008) | 0.972(0.932-1.015) | 0.993(0.973-1.013) | 1.001(0.992-1.009) | 0.997(0.952-1.044) | 0.994(0.915-1.08) | 1.004(0.904-1.116) | 1.04(0.932-1.161) | 1.103(0.978-1.243) |
| lag18 | 0.845(0.696-1.026) | 0.842(0.695-1.021) | 0.833(0.691-1.004) | 0.829(0.692-0.992) | 0.831(0.7-0.985) | 0.839(0.717-0.982) | 0.855(0.743-0.983) | 0.878(0.781-0.987) | 0.908(0.828-0.996) | 0.941(0.879-1.006) | 0.97(0.929-1.014) | 0.992(0.972-1.013) | 1.001(0.992-1.009) | 0.996(0.95-1.044) | 0.991(0.911-1.078) | 1.001(0.899-1.114) | 1.038(0.927-1.162) | 1.104(0.976-1.249) |
| lag19 | 0.83(0.679-1.014) | 0.827(0.678-1.009) | 0.817(0.673-0.991) | 0.813(0.675-0.979) | 0.815(0.683-0.973) | 0.824(0.7-0.97) | 0.841(0.728-0.972) | 0.867(0.768-0.979) | 0.9(0.818-0.99) | 0.936(0.873-1.003) | 0.968(0.925-1.013) | 0.992(0.971-1.014) | 1(0.991-1.009) | 0.994(0.947-1.044) | 0.988(0.906-1.077) | 0.997(0.893-1.113) | 1.037(0.924-1.164) | 1.109(0.977-1.26) |
| lag20 | 0.811(0.659-0.998) | 0.808(0.657-0.993) | 0.798(0.653-0.975) | 0.794(0.654-0.963) | 0.797(0.664-0.957) | 0.808(0.682-0.956) | 0.826(0.711-0.96) | 0.855(0.753-0.97) | 0.891(0.807-0.984) | 0.93(0.865-0.999) | 0.966(0.921-1.012) | 0.992(0.97-1.014) | 1(0.991-1.01) | 0.993(0.944-1.044) | 0.985(0.901-1.077) | 0.995(0.888-1.114) | 1.038(0.922-1.17) | 1.118(0.98-1.276) |
| lag21 | 0.788(0.634-0.979) | 0.785(0.633-0.974) | 0.775(0.629-0.956) | 0.772(0.631-0.945) | 0.777(0.641-0.94) | 0.789(0.661-0.941) | 0.81(0.692-0.947) | 0.841(0.737-0.959) | 0.88(0.793-0.977) | 0.923(0.856-0.996) | 0.962(0.916-1.011) | 0.991(0.968-1.014) | 1(0.99-1.01) | 0.991(0.941-1.045) | 0.982(0.895-1.078) | 0.993(0.882-1.118) | 1.041(0.919-1.18) | 1.13(0.983-1.299) |

| Table S26 Risk of 0y HFMD with each 1 °C change in lag time from 13.8 °C to 30 °C | | | | | | | | | | | | | | | | | | |
| --- | --- | --- | --- | --- | --- | --- | --- | --- | --- | --- | --- | --- | --- | --- | --- | --- | --- | --- |
| Lag(days) | Temperature(℃), RR(95%CI) | | | | | | | | | | | | | | | | | |
|  | 13.8 | 14 | 15 | 16 | 17 | 18 | 19 | 20 | 21 | 22 | 23 | 24 | 25 | 26 | 27 | 28 | 29 | 30 |
| lag0 | 0.792(0.699~0.898) | 0.798(0.706~0.901) | 0.823(0.738~0.919) | 0.846(0.765~0.936) | 0.866(0.789~0.951) | 0.882(0.811~0.959) | 0.894(0.832~0.96) | 0.902(0.851~0.957) | 0.912(0.869~0.956) | 0.925(0.889~0.963) | 0.945(0.918~0.974) | 0.975(0.962~0.988) | 1.009(1.004~1.014) | 1.043(1.023~1.063) | 1.069(1.039~1.1) | 1.084(1.043~1.126) | 1.08(1.031~1.131) | 1.057(1.006~1.111) |
| lag1 | 0.842(0.742~0.956) | 0.849(0.75~0.96) | 0.88(0.787~0.984) | 0.905(0.816~1.003) | 0.922(0.838~1.015) | 0.931(0.854~1.016) | 0.931(0.865~1.003) | 0.926(0.871~0.983) | 0.92(0.875~0.967) | 0.922(0.883~0.962) | 0.936(0.907~0.967) | 0.968(0.954~0.982) | 1.013(1.008~1.018) | 1.066(1.044~1.088) | 1.122(1.088~1.156) | 1.163(1.117~1.211) | 1.166(1.111~1.224) | 1.123(1.066~1.184) |
| lag2 | 0.905(0.797~1.027) | 0.911(0.806~1.031) | 0.942(0.842~1.054) | 0.963(0.867~1.07) | 0.975(0.883~1.075) | 0.975(0.892~1.065) | 0.963(0.892~1.039) | 0.944(0.887~1.005) | 0.926(0.879~0.976) | 0.919(0.878~0.961) | 0.929(0.897~0.961) | 0.962(0.946~0.977) | 1.016(1.011~1.022) | 1.091(1.067~1.115) | 1.181(1.144~1.218) | 1.258(1.207~1.311) | 1.272(1.212~1.335) | 1.204(1.145~1.267) |
| lag3 | 0.86(0.749~0.988) | 0.867(0.757~0.993) | 0.896(0.792~1.014) | 0.917(0.817~1.029) | 0.928(0.833~1.034) | 0.929(0.842~1.024) | 0.918(0.845~0.998) | 0.901(0.842~0.965) | 0.887(0.838~0.939) | 0.885(0.842~0.929) | 0.903(0.869~0.938) | 0.948(0.932~0.965) | 1.022(1.016~1.028) | 1.126(1.1~1.153) | 1.259(1.218~1.302) | 1.379(1.32~1.441) | 1.404(1.335~1.477) | 1.299(1.233~1.37) |
| lag4 | 0.758(0.653~0.881) | 0.765(0.66~0.886) | 0.793(0.693~0.907) | 0.815(0.719~0.924) | 0.83(0.738~0.933) | 0.836(0.753~0.929) | 0.834(0.762~0.912) | 0.827(0.769~0.889) | 0.824(0.776~0.875) | 0.834(0.792~0.879) | 0.867(0.833~0.903) | 0.931(0.914~0.948) | 1.029(1.023~1.036) | 1.168(1.14~1.198) | 1.351(1.305~1.399) | 1.52(1.453~1.591) | 1.555(1.475~1.638) | 1.404(1.329~1.482) |
| lag5 | 0.653(0.554~0.77) | 0.659(0.561~0.774) | 0.686(0.592~0.795) | 0.709(0.618~0.814) | 0.728(0.64~0.827) | 0.74(0.66~0.83) | 0.746(0.677~0.822) | 0.749(0.693~0.81) | 0.759(0.711~0.809) | 0.782(0.739~0.827) | 0.83(0.795~0.867) | 0.912(0.894~0.931) | 1.037(1.03~1.044) | 1.213(1.182~1.246) | 1.45(1.398~1.504) | 1.673(1.594~1.755) | 1.716(1.624~1.813) | 1.511(1.427~1.601) |
| lag6 | 0.57(0.475~0.684) | 0.575(0.481~0.688) | 0.601(0.51~0.708) | 0.625(0.536~0.728) | 0.645(0.56~0.743) | 0.661(0.583~0.751) | 0.673(0.605~0.749) | 0.684(0.628~0.746) | 0.702(0.654~0.754) | 0.736(0.692~0.782) | 0.796(0.759~0.835) | 0.896(0.876~0.915) | 1.044(1.037~1.052) | 1.257(1.222~1.294) | 1.549(1.489~1.611) | 1.828(1.736~1.925) | 1.88(1.772~1.995) | 1.618(1.521~1.72) |
| lag7 | 0.513(0.42~0.626) | 0.518(0.426~0.63) | 0.543(0.454~0.65) | 0.567(0.479~0.671) | 0.588(0.503~0.687) | 0.606(0.527~0.696) | 0.621(0.552~0.698) | 0.636(0.579~0.698) | 0.659(0.61~0.712) | 0.699(0.654~0.747) | 0.768(0.73~0.809) | 0.881(0.86~0.902) | 1.051(1.042~1.059) | 1.298(1.258~1.339) | 1.642(1.574~1.714) | 1.979(1.872~2.092) | 2.04(1.915~2.174) | 1.718(1.609~1.835) |
| lag8 | 0.478(0.385~0.593) | 0.483(0.391~0.597) | 0.508(0.418~0.617) | 0.531(0.443~0.637) | 0.552(0.467~0.653) | 0.571(0.491~0.663) | 0.586(0.517~0.665) | 0.603(0.545~0.667) | 0.628(0.578~0.682) | 0.671(0.625~0.721) | 0.746(0.707~0.788) | 0.869(0.847~0.891) | 1.056(1.047~1.065) | 1.333(1.29~1.378) | 1.728(1.652~1.808) | 2.122(2.001~2.25) | 2.192(2.05~2.344) | 1.811(1.69~1.941) |
| lag9 | 0.458(0.364~0.576) | 0.463(0.37~0.58) | 0.488(0.397~0.6) | 0.511(0.421~0.619) | 0.531(0.445~0.635) | 0.55(0.469~0.644) | 0.565(0.494~0.646) | 0.581(0.522~0.647) | 0.606(0.555~0.662) | 0.651(0.603~0.702) | 0.729(0.688~0.773) | 0.859(0.837~0.882) | 1.061(1.051~1.07) | 1.365(1.318~1.414) | 1.808(1.724~1.895) | 2.256(2.121~2.399) | 2.335(2.177~2.504) | 1.898(1.766~2.039) |
| lag10 | 0.448(0.352~0.571) | 0.454(0.358~0.575) | 0.478(0.384~0.595) | 0.501(0.408~0.614) | 0.521(0.432~0.629) | 0.539(0.456~0.637) | 0.553(0.48~0.636) | 0.567(0.507~0.635) | 0.591(0.539~0.649) | 0.636(0.587~0.689) | 0.716(0.674~0.761) | 0.851(0.828~0.875) | 1.065(1.055~1.075) | 1.393(1.343~1.445) | 1.88(1.789~1.975) | 2.382(2.234~2.539) | 2.47(2.297~2.656) | 1.977(1.835~2.131) |
| lag11 | 0.445(0.345~0.575) | 0.45(0.351~0.578) | 0.476(0.378~0.599) | 0.498(0.402~0.618) | 0.518(0.425~0.632) | 0.535(0.448~0.638) | 0.547(0.472~0.634) | 0.56(0.497~0.63) | 0.582(0.528~0.642) | 0.625(0.575~0.68) | 0.706(0.662~0.752) | 0.845(0.821~0.87) | 1.068(1.058~1.078) | 1.417(1.364~1.472) | 1.945(1.847~2.048) | 2.499(2.338~2.672) | 2.598(2.409~2.801) | 2.052(1.899~2.217) |
| lag12 | 0.446(0.341~0.583) | 0.451(0.347~0.587) | 0.477(0.375~0.608) | 0.5(0.399~0.627) | 0.52(0.422~0.64) | 0.535(0.445~0.644) | 0.546(0.467~0.638) | 0.556(0.491~0.63) | 0.576(0.52~0.638) | 0.618(0.566~0.675) | 0.698(0.653~0.747) | 0.84(0.815~0.866) | 1.071(1.06~1.082) | 1.438(1.382~1.497) | 2.005(1.9~2.116) | 2.61(2.435~2.798) | 2.719(2.514~2.941) | 2.121(1.958~2.298) |
| lag13 | 0.448(0.339~0.593) | 0.454(0.345~0.597) | 0.481(0.373~0.619) | 0.505(0.399~0.639) | 0.524(0.422~0.652) | 0.539(0.444~0.654) | 0.547(0.465~0.644) | 0.555(0.487~0.633) | 0.573(0.515~0.639) | 0.613(0.559~0.673) | 0.693(0.646~0.744) | 0.836(0.81~0.863) | 1.073(1.062~1.085) | 1.457(1.397~1.519) | 2.06(1.947~2.179) | 2.715(2.525~2.919) | 2.835(2.612~3.076) | 2.187(2.012~2.377) |
| lag14 | 0.45(0.336~0.603) | 0.456(0.342~0.607) | 0.484(0.372~0.631) | 0.509(0.398~0.651) | 0.529(0.421~0.664) | 0.543(0.443~0.665) | 0.55(0.464~0.653) | 0.556(0.485~0.638) | 0.572(0.511~0.641) | 0.61(0.554~0.672) | 0.689(0.64~0.742) | 0.833(0.805~0.862) | 1.075(1.063~1.087) | 1.473(1.41~1.539) | 2.11(1.99~2.238) | 2.815(2.61~3.036) | 2.947(2.706~3.209) | 2.251(2.063~2.455) |
| lag15 | 0.449(0.331~0.609) | 0.455(0.338~0.614) | 0.486(0.369~0.639) | 0.512(0.396~0.661) | 0.532(0.42~0.674) | 0.546(0.442~0.675) | 0.553(0.463~0.66) | 0.557(0.483~0.644) | 0.572(0.508~0.644) | 0.608(0.55~0.674) | 0.686(0.635~0.742) | 0.831(0.802~0.861) | 1.076(1.064~1.089) | 1.487(1.421~1.557) | 2.158(2.029~2.294) | 2.912(2.691~3.15) | 3.057(2.798~3.34) | 2.313(2.113~2.531) |
| lag16 | 0.444(0.324~0.609) | 0.451(0.331~0.615) | 0.483(0.363~0.643) | 0.511(0.392~0.666) | 0.533(0.417~0.681) | 0.548(0.44~0.682) | 0.554(0.461~0.667) | 0.558(0.481~0.648) | 0.572(0.505~0.647) | 0.607(0.546~0.676) | 0.685(0.631~0.742) | 0.829(0.799~0.86) | 1.078(1.065~1.091) | 1.5(1.43~1.573) | 2.202(2.066~2.346) | 3.006(2.771~3.262) | 3.167(2.89~3.471) | 2.374(2.162~2.607) |
| lag17 | 0.435(0.314~0.602) | 0.442(0.321~0.608) | 0.476(0.355~0.639) | 0.506(0.384~0.665) | 0.529(0.411~0.682) | 0.546(0.435~0.684) | 0.553(0.457~0.67) | 0.558(0.478~0.651) | 0.571(0.502~0.65) | 0.607(0.543~0.678) | 0.684(0.628~0.744) | 0.828(0.797~0.86) | 1.079(1.065~1.092) | 1.511(1.438~1.587) | 2.244(2.101~2.397) | 3.101(2.85~3.373) | 3.278(2.982~3.604) | 2.436(2.212~2.684) |
| lag18 | 0.419(0.3~0.586) | 0.427(0.307~0.593) | 0.463(0.342~0.626) | 0.495(0.373~0.656) | 0.521(0.402~0.676) | 0.54(0.428~0.681) | 0.549(0.451~0.669) | 0.555(0.473~0.652) | 0.57(0.499~0.651) | 0.606(0.54~0.68) | 0.683(0.626~0.745) | 0.827(0.795~0.861) | 1.08(1.066~1.094) | 1.521(1.445~1.601) | 2.285(2.135~2.445) | 3.195(2.93~3.484) | 3.392(3.077~3.739) | 2.5(2.262~2.762) |
| lag19 | 0.398(0.282~0.562) | 0.406(0.289~0.569) | 0.444(0.325~0.606) | 0.478(0.358~0.639) | 0.507(0.388~0.663) | 0.529(0.416~0.672) | 0.542(0.442~0.663) | 0.551(0.466~0.65) | 0.568(0.494~0.652) | 0.606(0.537~0.682) | 0.683(0.624~0.748) | 0.827(0.793~0.861) | 1.08(1.066~1.095) | 1.529(1.45~1.613) | 2.324(2.167~2.493) | 3.291(3.01~3.598) | 3.508(3.173~3.879) | 2.565(2.314~2.844) |
| lag20 | 0.371(0.26~0.53) | 0.379(0.268~0.538) | 0.419(0.304~0.577) | 0.456(0.338~0.614) | 0.488(0.37~0.643) | 0.513(0.401~0.657) | 0.53(0.43~0.654) | 0.543(0.458~0.645) | 0.564(0.488~0.651) | 0.604(0.534~0.685) | 0.683(0.621~0.751) | 0.827(0.792~0.863) | 1.081(1.065~1.096) | 1.537(1.455~1.624) | 2.363(2.198~2.541) | 3.389(3.089~3.717) | 3.63(3.27~4.029) | 2.633(2.365~2.931) |
| lag21 | 0.341(0.235~0.493) | 0.349(0.243~0.501) | 0.389(0.279~0.543) | 0.428(0.314~0.583) | 0.463(0.348~0.617) | 0.493(0.381~0.637) | 0.515(0.414~0.641) | 0.534(0.446~0.639) | 0.559(0.48~0.65) | 0.603(0.529~0.687) | 0.683(0.619~0.754) | 0.827(0.79~0.865) | 1.081(1.065~1.097) | 1.544(1.458~1.636) | 2.401(2.225~2.59) | 3.489(3.166~3.843) | 3.756(3.367~4.19) | 2.704(2.415~3.026) |

| Table S27 Risk of 1-5y HFMD with each 1 °C change in lag time from 13.8 °C to 30 °C | | | | | | | | | | | | | | | | | | |
| --- | --- | --- | --- | --- | --- | --- | --- | --- | --- | --- | --- | --- | --- | --- | --- | --- | --- | --- |
| Lag(days) | Temperature(℃), RR(95%CI) | | | | | | | | | | | | | | | | | |
|  | 13.8 | 14 | 15 | 16 | 17 | 18 | 19 | 20 | 21 | 22 | 23 | 24 | 25 | 26 | 27 | 28 | 29 | 30 |
| lag0 | 0.871(0.818~0.929) | 0.874(0.821~0.93) | 0.886(0.837~0.939) | 0.898(0.852~0.947) | 0.91(0.867~0.956) | 0.922(0.882~0.963) | 0.933(0.898~0.969) | 0.944(0.914~0.974) | 0.955(0.929~0.981) | 0.966(0.944~0.988) | 0.979(0.962~0.995) | 0.991(0.984~0.999) | 1.003(1~1.005) | 1.011(1~1.022) | 1.013(0.996~1.03) | 1.007(0.982~1.032) | 0.99(0.961~1.02) | 0.963(0.932~0.994) |
| lag1 | 0.939(0.88~1.002) | 0.939(0.881~1.001) | 0.942(0.888~0.999) | 0.944(0.894~0.997) | 0.947(0.9~0.996) | 0.949(0.907~0.993) | 0.952(0.915~0.989) | 0.955(0.924~0.986) | 0.959(0.932~0.986) | 0.965(0.942~0.989) | 0.975(0.957~0.993) | 0.988(0.98~0.996) | 1.005(1.002~1.008) | 1.026(1.014~1.038) | 1.051(1.032~1.07) | 1.067(1.04~1.095) | 1.058(1.026~1.091) | 1.017(0.983~1.051) |
| lag2 | 1.023(0.96~1.091) | 1.021(0.958~1.087) | 1.008(0.951~1.069) | 0.997(0.944~1.053) | 0.987(0.938~1.038) | 0.978(0.934~1.023) | 0.969(0.932~1.009) | 0.963(0.932~0.996) | 0.96(0.932~0.989) | 0.961(0.937~0.986) | 0.968(0.949~0.987) | 0.982(0.974~0.991) | 1.008(1.005~1.011) | 1.049(1.036~1.061) | 1.107(1.087~1.128) | 1.16(1.131~1.191) | 1.167(1.132~1.203) | 1.108(1.073~1.145) |
| lag3 | 1.022(0.953~1.096) | 1.018(0.951~1.091) | 1(0.938~1.066) | 0.983(0.926~1.044) | 0.969(0.916~1.024) | 0.956(0.909~1.005) | 0.945(0.906~0.987) | 0.938(0.904~0.973) | 0.935(0.906~0.965) | 0.938(0.912~0.965) | 0.95(0.929~0.971) | 0.972(0.963~0.982) | 1.012(1.009~1.016) | 1.078(1.063~1.092) | 1.175(1.152~1.199) | 1.269(1.235~1.305) | 1.29(1.249~1.332) | 1.204(1.164~1.245) |
| lag4 | 0.963(0.893~1.039) | 0.959(0.891~1.034) | 0.942(0.879~1.01) | 0.927(0.868~0.989) | 0.914(0.86~0.971) | 0.904(0.856~0.954) | 0.896(0.856~0.939) | 0.893(0.859~0.928) | 0.895(0.865~0.926) | 0.905(0.878~0.933) | 0.925(0.904~0.947) | 0.96(0.95~0.97) | 1.018(1.014~1.021) | 1.11(1.095~1.126) | 1.25(1.224~1.276) | 1.389(1.35~1.429) | 1.421(1.375~1.469) | 1.298(1.253~1.343) |
| lag5 | 0.885(0.814~0.961) | 0.882(0.812~0.957) | 0.868(0.804~0.937) | 0.857(0.798~0.92) | 0.849(0.795~0.906) | 0.843(0.795~0.894) | 0.841(0.8~0.884) | 0.843(0.809~0.879) | 0.852(0.822~0.884) | 0.87(0.842~0.899) | 0.9(0.878~0.923) | 0.947(0.937~0.958) | 1.023(1.019~1.027) | 1.144(1.127~1.161) | 1.327(1.299~1.357) | 1.513(1.468~1.559) | 1.556(1.503~1.611) | 1.388(1.339~1.439) |
| lag6 | 0.81(0.739~0.889) | 0.808(0.738~0.885) | 0.799(0.734~0.869) | 0.792(0.732~0.857) | 0.788(0.733~0.848) | 0.788(0.739~0.841) | 0.791(0.748~0.835) | 0.798(0.763~0.836) | 0.813(0.781~0.846) | 0.838(0.809~0.869) | 0.877(0.853~0.901) | 0.936(0.924~0.947) | 1.028(1.024~1.033) | 1.176(1.157~1.195) | 1.403(1.37~1.437) | 1.638(1.586~1.692) | 1.691(1.629~1.754) | 1.474(1.418~1.532) |
| lag7 | 0.751(0.678~0.83) | 0.749(0.678~0.827) | 0.743(0.677~0.815) | 0.74(0.679~0.807) | 0.74(0.683~0.801) | 0.743(0.692~0.798) | 0.75(0.706~0.796) | 0.762(0.725~0.801) | 0.781(0.748~0.816) | 0.812(0.781~0.844) | 0.857(0.832~0.883) | 0.926(0.913~0.938) | 1.033(1.028~1.037) | 1.205(1.184~1.227) | 1.475(1.438~1.513) | 1.759(1.699~1.822) | 1.822(1.751~1.896) | 1.555(1.492~1.62) |
| lag8 | 0.706(0.633~0.787) | 0.705(0.633~0.785) | 0.701(0.635~0.775) | 0.701(0.638~0.769) | 0.703(0.645~0.766) | 0.709(0.657~0.765) | 0.719(0.674~0.766) | 0.733(0.695~0.774) | 0.756(0.722~0.793) | 0.791(0.759~0.825) | 0.842(0.815~0.869) | 0.917(0.904~0.931) | 1.037(1.032~1.042) | 1.231(1.208~1.254) | 1.541(1.5~1.583) | 1.876(1.808~1.946) | 1.948(1.869~2.032) | 1.63(1.561~1.703) |
| lag9 | 0.672(0.598~0.755) | 0.671(0.599~0.753) | 0.67(0.602~0.745) | 0.671(0.608~0.741) | 0.676(0.617~0.74) | 0.683(0.63~0.741) | 0.695(0.649~0.744) | 0.712(0.673~0.754) | 0.738(0.702~0.775) | 0.775(0.742~0.81) | 0.83(0.802~0.858) | 0.911(0.897~0.925) | 1.04(1.035~1.045) | 1.253(1.228~1.279) | 1.603(1.558~1.649) | 1.986(1.911~2.064) | 2.069(1.981~2.162) | 1.7(1.626~1.779) |
| lag10 | 0.647(0.572~0.732) | 0.647(0.573~0.73) | 0.646(0.577~0.724) | 0.649(0.584~0.721) | 0.655(0.595~0.721) | 0.664(0.61~0.723) | 0.677(0.63~0.728) | 0.696(0.655~0.739) | 0.723(0.687~0.762) | 0.763(0.729~0.8) | 0.82(0.792~0.85) | 0.906(0.891~0.92) | 1.042(1.037~1.048) | 1.273(1.247~1.3) | 1.659(1.611~1.709) | 2.091(2.009~2.177) | 2.185(2.088~2.286) | 1.766(1.685~1.85) |
| lag11 | 0.628(0.551~0.715) | 0.628(0.552~0.713) | 0.629(0.558~0.708) | 0.632(0.566~0.707) | 0.639(0.578~0.708) | 0.65(0.594~0.711) | 0.664(0.615~0.717) | 0.684(0.642~0.729) | 0.713(0.675~0.753) | 0.755(0.719~0.792) | 0.814(0.784~0.845) | 0.901(0.886~0.917) | 1.045(1.039~1.051) | 1.29(1.262~1.319) | 1.711(1.659~1.764) | 2.191(2.101~2.284) | 2.296(2.19~2.406) | 1.826(1.74~1.916) |
| lag12 | 0.613(0.535~0.703) | 0.613(0.536~0.702) | 0.615(0.543~0.697) | 0.62(0.552~0.696) | 0.628(0.564~0.698) | 0.639(0.582~0.703) | 0.655(0.604~0.709) | 0.676(0.632~0.722) | 0.706(0.666~0.748) | 0.748(0.711~0.788) | 0.809(0.777~0.841) | 0.898(0.882~0.914) | 1.046(1.04~1.053) | 1.305(1.276~1.336) | 1.758(1.703~1.816) | 2.286(2.189~2.387) | 2.402(2.287~2.522) | 1.883(1.791~1.979) |
| lag13 | 0.602(0.522~0.695) | 0.602(0.523~0.693) | 0.605(0.531~0.689) | 0.61(0.54~0.689) | 0.619(0.554~0.692) | 0.631(0.572~0.697) | 0.648(0.595~0.704) | 0.67(0.625~0.719) | 0.701(0.66~0.745) | 0.744(0.705~0.786) | 0.805(0.773~0.839) | 0.896(0.879~0.913) | 1.048(1.042~1.054) | 1.319(1.287~1.351) | 1.802(1.743~1.864) | 2.377(2.271~2.487) | 2.504(2.38~2.635) | 1.936(1.837~2.039) |
| lag14 | 0.593(0.51~0.689) | 0.593(0.512~0.687) | 0.596(0.52~0.684) | 0.603(0.531~0.684) | 0.612(0.545~0.688) | 0.625(0.564~0.693) | 0.643(0.589~0.702) | 0.666(0.619~0.717) | 0.698(0.655~0.744) | 0.742(0.701~0.785) | 0.803(0.769~0.839) | 0.894(0.877~0.912) | 1.049(1.042~1.056) | 1.33(1.297~1.364) | 1.843(1.78~1.909) | 2.464(2.35~2.583) | 2.603(2.468~2.745) | 1.986(1.881~2.097) |
| lag15 | 0.585(0.5~0.684) | 0.585(0.502~0.682) | 0.589(0.511~0.679) | 0.596(0.522~0.681) | 0.606(0.537~0.685) | 0.62(0.557~0.691) | 0.639(0.583~0.7) | 0.663(0.614~0.716) | 0.696(0.651~0.744) | 0.741(0.698~0.786) | 0.802(0.767~0.84) | 0.893(0.875~0.912) | 1.05(1.043~1.057) | 1.34(1.305~1.376) | 1.881(1.814~1.951) | 2.548(2.426~2.677) | 2.699(2.555~2.853) | 2.034(1.922~2.152) |
| lag16 | 0.577(0.491~0.679) | 0.578(0.493~0.678) | 0.583(0.503~0.675) | 0.59(0.514~0.677) | 0.601(0.53~0.682) | 0.616(0.551~0.689) | 0.636(0.578~0.7) | 0.662(0.611~0.717) | 0.696(0.649~0.746) | 0.741(0.697~0.789) | 0.802(0.765~0.841) | 0.893(0.874~0.912) | 1.051(1.043~1.058) | 1.349(1.313~1.386) | 1.918(1.846~1.992) | 2.631(2.5~2.768) | 2.794(2.639~2.959) | 2.08(1.961~2.205) |
| lag17 | 0.57(0.482~0.674) | 0.571(0.484~0.673) | 0.576(0.494~0.671) | 0.584(0.507~0.673) | 0.596(0.524~0.679) | 0.613(0.546~0.688) | 0.633(0.574~0.699) | 0.66(0.608~0.718) | 0.696(0.647~0.748) | 0.742(0.696~0.792) | 0.803(0.764~0.844) | 0.893(0.873~0.913) | 1.051(1.044~1.059) | 1.357(1.319~1.396) | 1.952(1.877~2.03) | 2.711(2.572~2.858) | 2.888(2.723~3.064) | 2.124(1.999~2.257) |
| lag18 | 0.562(0.473~0.668) | 0.563(0.475~0.667) | 0.569(0.486~0.665) | 0.578(0.5~0.669) | 0.591(0.517~0.676) | 0.609(0.54~0.686) | 0.631(0.57~0.699) | 0.66(0.605~0.719) | 0.696(0.646~0.751) | 0.744(0.695~0.795) | 0.804(0.764~0.847) | 0.893(0.872~0.914) | 1.052(1.044~1.06) | 1.364(1.324~1.405) | 1.985(1.906~2.067) | 2.791(2.644~2.947) | 2.982(2.806~3.169) | 2.168(2.036~2.308) |
| lag19 | 0.553(0.463~0.661) | 0.554(0.466~0.66) | 0.561(0.477~0.659) | 0.571(0.492~0.664) | 0.585(0.51~0.672) | 0.604(0.535~0.683) | 0.628(0.566~0.698) | 0.659(0.603~0.72) | 0.697(0.645~0.754) | 0.746(0.695~0.8) | 0.806(0.764~0.85) | 0.893(0.872~0.915) | 1.052(1.044~1.06) | 1.37(1.329~1.413) | 2.017(1.934~2.102) | 2.871(2.715~3.035) | 3.076(2.889~3.274) | 2.211(2.073~2.359) |
| lag20 | 0.544(0.453~0.653) | 0.545(0.455~0.652) | 0.552(0.468~0.652) | 0.564(0.483~0.658) | 0.579(0.503~0.667) | 0.599(0.528~0.68) | 0.625(0.561~0.697) | 0.658(0.6~0.722) | 0.699(0.644~0.758) | 0.748(0.695~0.805) | 0.808(0.765~0.854) | 0.894(0.872~0.916) | 1.052(1.044~1.061) | 1.376(1.333~1.42) | 2.047(1.961~2.138) | 2.95(2.785~3.125) | 3.17(2.971~3.382) | 2.254(2.107~2.411) |
| lag21 | 0.533(0.441~0.644) | 0.534(0.444~0.643) | 0.543(0.457~0.644) | 0.555(0.473~0.651) | 0.572(0.494~0.662) | 0.594(0.521~0.677) | 0.622(0.556~0.697) | 0.657(0.597~0.724) | 0.7(0.642~0.763) | 0.751(0.695~0.81) | 0.811(0.765~0.859) | 0.895(0.872~0.918) | 1.052(1.043~1.061) | 1.381(1.336~1.428) | 2.077(1.985~2.173) | 3.029(2.851~3.218) | 3.265(3.05~3.495) | 2.296(2.139~2.465) |

| Table S28 Risk of 6-20y HFMD with each 1 °C change in lag time from 13.8 °C to 30 °C | | | | | | | | | | | | | | | | | | |
| --- | --- | --- | --- | --- | --- | --- | --- | --- | --- | --- | --- | --- | --- | --- | --- | --- | --- | --- |
| Lag(days) | Temperature(℃), RR(95%CI) | | | | | | | | | | | | | | | | | |
|  | 13.8 | 14 | 15 | 16 | 17 | 18 | 19 | 20 | 21 | 22 | 23 | 24 | 25 | 26 | 27 | 28 | 29 | 30 |
| lag0 | 0.768(0.665~0.887) | 0.771(0.669~0.887) | 0.784(0.688~0.892) | 0.797(0.707~0.899) | 0.81(0.725~0.905) | 0.823(0.745~0.91) | 0.836(0.767~0.911) | 0.85(0.79~0.914) | 0.867(0.815~0.922) | 0.891(0.845~0.939) | 0.923(0.888~0.96) | 0.966(0.949~0.983) | 1.012(1.006~1.018) | 1.055(1.029~1.081) | 1.085(1.043~1.129) | 1.1(1.039~1.164) | 1.097(1.024~1.175) | 1.079(1~1.163) |
| lag1 | 0.768(0.663~0.89) | 0.77(0.666~0.89) | 0.781(0.683~0.893) | 0.791(0.698~0.896) | 0.801(0.714~0.899) | 0.811(0.731~0.9) | 0.821(0.75~0.898) | 0.832(0.771~0.897) | 0.848(0.794~0.905) | 0.872(0.824~0.922) | 0.908(0.87~0.947) | 0.958(0.94~0.976) | 1.015(1.009~1.022) | 1.072(1.044~1.101) | 1.119(1.073~1.167) | 1.144(1.077~1.215) | 1.139(1.059~1.224) | 1.1(1.017~1.191) |
| lag2 | 0.816(0.704~0.944) | 0.817(0.707~0.943) | 0.822(0.718~0.94) | 0.826(0.728~0.937) | 0.83(0.738~0.933) | 0.833(0.75~0.926) | 0.836(0.763~0.916) | 0.84(0.777~0.908) | 0.85(0.794~0.91) | 0.869(0.818~0.923) | 0.903(0.863~0.945) | 0.954(0.935~0.974) | 1.017(1.01~1.024) | 1.085(1.055~1.117) | 1.148(1.099~1.199) | 1.188(1.117~1.263) | 1.181(1.099~1.27) | 1.123(1.04~1.213) |
| lag3 | 0.825(0.703~0.967) | 0.826(0.706~0.966) | 0.829(0.716~0.96) | 0.831(0.724~0.954) | 0.831(0.731~0.945) | 0.83(0.74~0.932) | 0.827(0.749~0.914) | 0.826(0.759~0.899) | 0.831(0.772~0.895) | 0.849(0.794~0.907) | 0.885(0.841~0.931) | 0.944(0.923~0.966) | 1.022(1.014~1.029) | 1.11(1.076~1.145) | 1.199(1.143~1.256) | 1.26(1.18~1.345) | 1.256(1.164~1.354) | 1.176(1.086~1.273) |
| lag4 | 0.804(0.677~0.954) | 0.805(0.68~0.953) | 0.81(0.691~0.949) | 0.812(0.7~0.943) | 0.812(0.707~0.932) | 0.808(0.714~0.915) | 0.802(0.721~0.892) | 0.797(0.728~0.871) | 0.799(0.738~0.865) | 0.817(0.761~0.876) | 0.859(0.813~0.906) | 0.93(0.907~0.953) | 1.027(1.019~1.036) | 1.144(1.106~1.182) | 1.265(1.204~1.328) | 1.353(1.265~1.448) | 1.354(1.253~1.463) | 1.25(1.153~1.355) |
| lag5 | 0.771(0.64~0.929) | 0.773(0.643~0.929) | 0.78(0.657~0.927) | 0.784(0.667~0.922) | 0.784(0.675~0.911) | 0.78(0.682~0.892) | 0.771(0.687~0.864) | 0.762(0.692~0.839) | 0.763(0.701~0.83) | 0.782(0.725~0.844) | 0.83(0.782~0.88) | 0.915(0.891~0.939) | 1.034(1.025~1.043) | 1.181(1.14~1.223) | 1.339(1.271~1.41) | 1.459(1.359~1.566) | 1.465(1.351~1.588) | 1.334(1.227~1.451) |
| lag6 | 0.738(0.601~0.906) | 0.741(0.605~0.907) | 0.751(0.622~0.907) | 0.757(0.633~0.904) | 0.757(0.642~0.892) | 0.752(0.649~0.871) | 0.741(0.654~0.84) | 0.73(0.657~0.811) | 0.729(0.665~0.8) | 0.749(0.689~0.814) | 0.802(0.753~0.855) | 0.9(0.874~0.927) | 1.04(1.03~1.05) | 1.217(1.172~1.265) | 1.414(1.337~1.494) | 1.567(1.452~1.691) | 1.578(1.448~1.72) | 1.418(1.297~1.551) |
| lag7 | 0.712(0.569~0.891) | 0.715(0.573~0.891) | 0.727(0.591~0.894) | 0.734(0.604~0.891) | 0.734(0.614~0.879) | 0.728(0.62~0.855) | 0.715(0.624~0.82) | 0.702(0.627~0.787) | 0.7(0.634~0.774) | 0.721(0.659~0.789) | 0.779(0.726~0.835) | 0.887(0.859~0.915) | 1.046(1.035~1.057) | 1.252(1.201~1.305) | 1.485(1.399~1.576) | 1.671(1.54~1.813) | 1.687(1.539~1.849) | 1.497(1.361~1.645) |
| lag8 | 0.692(0.544~0.882) | 0.696(0.548~0.883) | 0.71(0.568~0.886) | 0.717(0.582~0.884) | 0.717(0.591~0.87) | 0.71(0.598~0.844) | 0.695(0.601~0.805) | 0.68(0.602~0.769) | 0.677(0.608~0.753) | 0.697(0.633~0.768) | 0.759(0.704~0.817) | 0.876(0.847~0.906) | 1.051(1.04~1.063) | 1.283(1.227~1.341) | 1.551(1.456~1.652) | 1.769(1.623~1.928) | 1.788(1.624~1.97) | 1.566(1.418~1.729) |
| lag9 | 0.679(0.525~0.879) | 0.683(0.53~0.88) | 0.697(0.55~0.884) | 0.705(0.564~0.88) | 0.705(0.574~0.865) | 0.696(0.58~0.836) | 0.679(0.581~0.794) | 0.662(0.581~0.754) | 0.657(0.586~0.736) | 0.678(0.612~0.751) | 0.742(0.686~0.802) | 0.866(0.836~0.898) | 1.056(1.043~1.068) | 1.311(1.251~1.373) | 1.612(1.509~1.723) | 1.861(1.701~2.037) | 1.883(1.703~2.083) | 1.628(1.469~1.804) |
| lag10 | 0.671(0.51~0.881) | 0.674(0.515~0.883) | 0.689(0.536~0.886) | 0.696(0.55~0.881) | 0.695(0.56~0.864) | 0.685(0.565~0.831) | 0.667(0.566~0.786) | 0.647(0.564~0.743) | 0.641(0.568~0.723) | 0.661(0.594~0.737) | 0.727(0.669~0.79) | 0.858(0.826~0.89) | 1.06(1.047~1.073) | 1.336(1.273~1.403) | 1.67(1.558~1.79) | 1.949(1.775~2.141) | 1.974(1.778~2.191) | 1.684(1.514~1.872) |
| lag11 | 0.666(0.499~0.888) | 0.67(0.504~0.889) | 0.684(0.525~0.891) | 0.69(0.539~0.885) | 0.688(0.548~0.865) | 0.677(0.552~0.829) | 0.656(0.552~0.78) | 0.635(0.549~0.734) | 0.627(0.553~0.712) | 0.648(0.578~0.725) | 0.715(0.655~0.78) | 0.85(0.817~0.885) | 1.063(1.05~1.077) | 1.36(1.292~1.432) | 1.725(1.605~1.854) | 2.036(1.847~2.244) | 2.061(1.85~2.297) | 1.736(1.555~1.937) |
| lag12 | 0.664(0.49~0.9) | 0.668(0.495~0.9) | 0.681(0.516~0.9) | 0.687(0.529~0.891) | 0.683(0.537~0.868) | 0.67(0.541~0.829) | 0.647(0.54~0.776) | 0.624(0.536~0.727) | 0.615(0.539~0.703) | 0.636(0.564~0.716) | 0.704(0.643~0.772) | 0.844(0.81~0.88) | 1.067(1.052~1.081) | 1.382(1.31~1.458) | 1.779(1.65~1.918) | 2.122(1.917~2.349) | 2.149(1.921~2.405) | 1.785(1.593~2.001) |
| lag13 | 0.665(0.484~0.914) | 0.668(0.488~0.914) | 0.68(0.508~0.911) | 0.684(0.52~0.899) | 0.679(0.528~0.873) | 0.664(0.531~0.83) | 0.64(0.529~0.774) | 0.615(0.524~0.722) | 0.605(0.526~0.696) | 0.625(0.552~0.709) | 0.695(0.631~0.765) | 0.838(0.802~0.875) | 1.07(1.054~1.085) | 1.403(1.327~1.484) | 1.832(1.694~1.982) | 2.21(1.988~2.457) | 2.239(1.992~2.517) | 1.835(1.63~2.066) |
| lag14 | 0.667(0.478~0.931) | 0.67(0.483~0.93) | 0.68(0.501~0.924) | 0.682(0.513~0.908) | 0.675(0.519~0.878) | 0.658(0.521~0.832) | 0.632(0.518~0.772) | 0.607(0.513~0.717) | 0.596(0.515~0.691) | 0.616(0.54~0.702) | 0.687(0.621~0.759) | 0.833(0.796~0.872) | 1.072(1.056~1.088) | 1.424(1.343~1.51) | 1.886(1.738~2.047) | 2.302(2.062~2.571) | 2.334(2.067~2.637) | 1.887(1.668~2.135) |
| lag15 | 0.671(0.474~0.949) | 0.673(0.479~0.947) | 0.681(0.495~0.937) | 0.681(0.506~0.917) | 0.672(0.511~0.883) | 0.653(0.511~0.833) | 0.625(0.508~0.77) | 0.599(0.502~0.714) | 0.588(0.504~0.686) | 0.608(0.529~0.697) | 0.679(0.611~0.754) | 0.828(0.789~0.868) | 1.075(1.058~1.092) | 1.445(1.359~1.536) | 1.943(1.784~2.115) | 2.401(2.141~2.693) | 2.438(2.148~2.767) | 1.942(1.708~2.209) |
| lag16 | 0.675(0.471~0.968) | 0.677(0.475~0.965) | 0.682(0.49~0.949) | 0.679(0.499~0.925) | 0.668(0.502~0.887) | 0.647(0.502~0.834) | 0.618(0.498~0.768) | 0.591(0.492~0.709) | 0.58(0.493~0.681) | 0.6(0.519~0.692) | 0.672(0.602~0.75) | 0.823(0.784~0.865) | 1.078(1.06~1.095) | 1.465(1.375~1.562) | 2.002(1.833~2.187) | 2.509(2.228~2.826) | 2.552(2.238~2.909) | 2.003(1.753~2.29) |
| lag17 | 0.68(0.469~0.987) | 0.682(0.473~0.983) | 0.683(0.486~0.961) | 0.677(0.492~0.931) | 0.663(0.494~0.889) | 0.641(0.493~0.832) | 0.611(0.488~0.764) | 0.583(0.482~0.705) | 0.572(0.484~0.676) | 0.592(0.51~0.688) | 0.665(0.593~0.746) | 0.819(0.778~0.862) | 1.08(1.062~1.099) | 1.487(1.392~1.588) | 2.066(1.886~2.263) | 2.629(2.326~2.971) | 2.679(2.34~3.067) | 2.072(1.804~2.38) |
| lag18 | 0.686(0.468~1.006) | 0.686(0.471~1) | 0.684(0.481~0.971) | 0.674(0.486~0.936) | 0.657(0.486~0.889) | 0.633(0.484~0.83) | 0.603(0.478~0.76) | 0.575(0.472~0.7) | 0.564(0.474~0.671) | 0.585(0.501~0.684) | 0.659(0.585~0.742) | 0.815(0.772~0.86) | 1.083(1.064~1.102) | 1.509(1.41~1.615) | 2.136(1.944~2.345) | 2.762(2.435~3.132) | 2.824(2.457~3.246) | 2.151(1.864~2.482) |
| lag19 | 0.691(0.466~1.024) | 0.69(0.468~1.017) | 0.683(0.476~0.98) | 0.67(0.478~0.939) | 0.651(0.477~0.888) | 0.625(0.474~0.825) | 0.594(0.468~0.754) | 0.566(0.462~0.694) | 0.556(0.464~0.666) | 0.578(0.492~0.679) | 0.653(0.577~0.739) | 0.811(0.767~0.857) | 1.085(1.066~1.105) | 1.532(1.428~1.644) | 2.211(2.008~2.435) | 2.911(2.558~3.313) | 2.989(2.59~3.449) | 2.24(1.931~2.599) |
| lag20 | 0.696(0.464~1.044) | 0.694(0.466~1.035) | 0.682(0.471~0.989) | 0.665(0.47~0.941) | 0.643(0.467~0.886) | 0.616(0.463~0.82) | 0.585(0.457~0.748) | 0.557(0.451~0.688) | 0.548(0.454~0.662) | 0.571(0.482~0.676) | 0.647(0.569~0.735) | 0.806(0.761~0.854) | 1.088(1.068~1.109) | 1.557(1.447~1.675) | 2.294(2.076~2.534) | 3.08(2.695~3.521) | 3.178(2.739~3.686) | 2.343(2.007~2.736) |
| lag21 | 0.701(0.461~1.066) | 0.698(0.462~1.054) | 0.68(0.464~0.999) | 0.659(0.461~0.944) | 0.634(0.456~0.883) | 0.606(0.45~0.815) | 0.574(0.444~0.742) | 0.548(0.44~0.683) | 0.54(0.443~0.657) | 0.564(0.472~0.673) | 0.641(0.56~0.733) | 0.802(0.755~0.852) | 1.091(1.069~1.112) | 1.583(1.467~1.708) | 2.384(2.149~2.646) | 3.271(2.845~3.761) | 3.394(2.904~3.966) | 2.461(2.089~2.898) |

| Table S29 Risk of >20y HFMD with each 1 °C change in lag time from 13.8 °C to 30 °C | | | | | | | | | | | | | | | | | | |
| --- | --- | --- | --- | --- | --- | --- | --- | --- | --- | --- | --- | --- | --- | --- | --- | --- | --- | --- |
| Lag(days) | Temperature(℃), RR(95%CI) | | | | | | | | | | | | | | | | | |
|  | 13.8 | 14 | 15 | 16 | 17 | 18 | 19 | 20 | 21 | 22 | 23 | 24 | 25 | 26 | 27 | 28 | 29 | 30 |
| lag0 | 0.921(0.668~1.271) | 0.92(0.671~1.262) | 0.916(0.684~1.226) | 0.917(0.699~1.203) | 0.923(0.718~1.187) | 0.935(0.746~1.173) | 0.955(0.785~1.161) | 0.978(0.829~1.154) | 1.001(0.868~1.153) | 1.017(0.9~1.148) | 1.021(0.932~1.118) | 1.011(0.97~1.053) | 0.996(0.983~1.01) | 0.985(0.93~1.044) | 0.986(0.899~1.081) | 0.997(0.873~1.139) | 1.015(0.864~1.191) | 1.035(0.87~1.231) |
| lag1 | 0.991(0.714~1.376) | 0.988(0.716~1.365) | 0.977(0.724~1.317) | 0.967(0.731~1.28) | 0.961(0.741~1.246) | 0.957(0.757~1.21) | 0.957(0.78~1.173) | 0.959(0.806~1.141) | 0.964(0.829~1.121) | 0.971(0.852~1.108) | 0.98(0.888~1.082) | 0.99(0.947~1.035) | 1.004(0.989~1.019) | 1.023(0.961~1.089) | 1.049(0.95~1.158) | 1.078(0.936~1.243) | 1.103(0.93~1.307) | 1.118(0.932~1.342) |
| lag2 | 1.087(0.783~1.51) | 1.084(0.784~1.497) | 1.064(0.787~1.439) | 1.043(0.784~1.386) | 1.019(0.781~1.329) | 0.994(0.781~1.264) | 0.967(0.784~1.192) | 0.941(0.786~1.126) | 0.923(0.787~1.082) | 0.917(0.796~1.056) | 0.93(0.835~1.035) | 0.964(0.918~1.012) | 1.014(0.998~1.031) | 1.075(1.005~1.15) | 1.139(1.026~1.264) | 1.193(1.03~1.381) | 1.22(1.028~1.449) | 1.215(1.016~1.452) |
| lag3 | 1.135(0.792~1.626) | 1.131(0.794~1.61) | 1.108(0.796~1.542) | 1.079(0.79~1.475) | 1.046(0.781~1.4) | 1.007(0.774~1.311) | 0.964(0.767~1.212) | 0.922(0.758~1.122) | 0.891(0.748~1.06) | 0.879(0.753~1.026) | 0.895(0.795~1.008) | 0.945(0.896~0.997) | 1.022(1.004~1.041) | 1.118(1.039~1.204) | 1.221(1.092~1.366) | 1.306(1.118~1.526) | 1.339(1.118~1.602) | 1.303(1.083~1.568) |
| lag4 | 1.14(0.772~1.682) | 1.136(0.775~1.666) | 1.114(0.779~1.593) | 1.084(0.773~1.52) | 1.047(0.765~1.434) | 1.003(0.755~1.332) | 0.952(0.745~1.217) | 0.903(0.732~1.113) | 0.866(0.719~1.043) | 0.852(0.722~1.006) | 0.872(0.768~0.99) | 0.932(0.88~0.987) | 1.028(1.009~1.048) | 1.154(1.068~1.247) | 1.297(1.155~1.456) | 1.419(1.209~1.666) | 1.459(1.213~1.754) | 1.389(1.149~1.679) |
| lag5 | 1.121(0.732~1.716) | 1.118(0.735~1.701) | 1.099(0.743~1.627) | 1.072(0.741~1.551) | 1.035(0.734~1.46) | 0.99(0.727~1.349) | 0.937(0.718~1.223) | 0.884(0.705~1.109) | 0.846(0.692~1.033) | 0.831(0.695~0.995) | 0.854(0.744~0.98) | 0.922(0.866~0.981) | 1.033(1.012~1.055) | 1.185(1.091~1.288) | 1.37(1.213~1.547) | 1.534(1.297~1.815) | 1.585(1.308~1.921) | 1.48(1.215~1.803) |
| lag6 | 1.092(0.681~1.75) | 1.09(0.685~1.735) | 1.077(0.698~1.662) | 1.054(0.7~1.586) | 1.02(0.697~1.491) | 0.975(0.693~1.372) | 0.921(0.686~1.236) | 0.867(0.676~1.112) | 0.827(0.664~1.03) | 0.813(0.668~0.99) | 0.838(0.721~0.975) | 0.912(0.852~0.977) | 1.037(1.014~1.061) | 1.215(1.11~1.33) | 1.443(1.266~1.645) | 1.656(1.384~1.982) | 1.723(1.405~2.114) | 1.583(1.282~1.954) |
| lag7 | 1.062(0.632~1.783) | 1.062(0.637~1.768) | 1.055(0.655~1.7) | 1.036(0.661~1.624) | 1.005(0.662~1.526) | 0.961(0.66~1.399) | 0.906(0.656~1.251) | 0.85(0.647~1.116) | 0.809(0.637~1.028) | 0.795(0.642~0.985) | 0.822(0.698~0.969) | 0.903(0.838~0.973) | 1.042(1.017~1.068) | 1.247(1.13~1.375) | 1.52(1.321~1.749) | 1.787(1.475~2.164) | 1.876(1.509~2.331) | 1.702(1.36~2.129) |
| lag8 | 1.033(0.589~1.812) | 1.035(0.596~1.799) | 1.035(0.618~1.735) | 1.021(0.628~1.661) | 0.993(0.632~1.56) | 0.949(0.632~1.425) | 0.893(0.63~1.265) | 0.834(0.622~1.119) | 0.791(0.611~1.023) | 0.777(0.617~0.977) | 0.806(0.676~0.961) | 0.894(0.825~0.968) | 1.047(1.02~1.075) | 1.279(1.152~1.419) | 1.6(1.38~1.856) | 1.926(1.575~2.357) | 2.043(1.626~2.567) | 1.838(1.453~2.325) |
| lag9 | 1.007(0.552~1.838) | 1.01(0.559~1.826) | 1.017(0.585~1.769) | 1.008(0.598~1.698) | 0.982(0.605~1.593) | 0.938(0.607~1.449) | 0.88(0.606~1.277) | 0.819(0.598~1.12) | 0.773(0.588~1.017) | 0.758(0.594~0.967) | 0.79(0.655~0.952) | 0.884(0.812~0.962) | 1.052(1.023~1.081) | 1.312(1.175~1.464) | 1.684(1.442~1.967) | 2.074(1.681~2.559) | 2.224(1.755~2.818) | 1.991(1.56~2.541) |
| lag10 | 0.983(0.519~1.863) | 0.987(0.526~1.853) | 1(0.555~1.803) | 0.995(0.571~1.733) | 0.971(0.58~1.625) | 0.928(0.584~1.473) | 0.867(0.583~1.289) | 0.803(0.575~1.12) | 0.755(0.565~1.01) | 0.74(0.572~0.957) | 0.773(0.635~0.942) | 0.874(0.8~0.956) | 1.056(1.026~1.088) | 1.345(1.198~1.51) | 1.769(1.505~2.08) | 2.229(1.793~2.77) | 2.418(1.894~3.087) | 2.158(1.677~2.776) |
| lag11 | 0.96(0.488~1.889) | 0.966(0.496~1.881) | 0.984(0.527~1.836) | 0.982(0.545~1.769) | 0.96(0.556~1.657) | 0.916(0.561~1.496) | 0.854(0.561~1.299) | 0.787(0.553~1.12) | 0.737(0.543~1.002) | 0.722(0.55~0.947) | 0.757(0.616~0.932) | 0.865(0.787~0.95) | 1.061(1.029~1.094) | 1.378(1.221~1.556) | 1.856(1.568~2.196) | 2.388(1.906~2.992) | 2.621(2.036~3.373) | 2.336(1.8~3.031) |
| lag12 | 0.939(0.46~1.918) | 0.946(0.468~1.911) | 0.967(0.5~1.871) | 0.969(0.52~1.804) | 0.947(0.532~1.688) | 0.903(0.538~1.517) | 0.839(0.538~1.309) | 0.771(0.531~1.119) | 0.72(0.52~0.995) | 0.704(0.529~0.937) | 0.742(0.596~0.923) | 0.856(0.775~0.944) | 1.066(1.032~1.101) | 1.411(1.243~1.602) | 1.942(1.629~2.315) | 2.549(2.017~3.223) | 2.83(2.179~3.676) | 2.522(1.926~3.303) |
| lag13 | 0.919(0.433~1.949) | 0.926(0.442~1.942) | 0.951(0.475~1.904) | 0.954(0.495~1.836) | 0.933(0.507~1.716) | 0.889(0.514~1.536) | 0.823(0.515~1.316) | 0.754(0.508~1.118) | 0.702(0.499~0.989) | 0.687(0.508~0.929) | 0.727(0.578~0.915) | 0.847(0.763~0.94) | 1.07(1.034~1.107) | 1.443(1.262~1.649) | 2.027(1.687~2.435) | 2.711(2.124~3.46) | 3.042(2.318~3.993) | 2.711(2.048~3.59) |
| lag14 | 0.9(0.409~1.98) | 0.908(0.417~1.973) | 0.933(0.45~1.934) | 0.937(0.471~1.864) | 0.916(0.483~1.738) | 0.872(0.491~1.55) | 0.806(0.492~1.321) | 0.736(0.486~1.115) | 0.684(0.477~0.982) | 0.67(0.488~0.921) | 0.713(0.56~0.909) | 0.839(0.752~0.936) | 1.074(1.037~1.113) | 1.473(1.28~1.694) | 2.109(1.741~2.554) | 2.869(2.225~3.699) | 3.252(2.45~4.315) | 2.899(2.165~3.882) |
| lag15 | 0.883(0.388~2.01) | 0.89(0.395~2.002) | 0.915(0.427~1.959) | 0.918(0.447~1.884) | 0.897(0.459~1.752) | 0.852(0.467~1.557) | 0.787(0.469~1.321) | 0.717(0.464~1.109) | 0.667(0.456~0.975) | 0.654(0.468~0.914) | 0.7(0.543~0.903) | 0.831(0.741~0.932) | 1.078(1.038~1.119) | 1.501(1.297~1.738) | 2.187(1.791~2.67) | 3.021(2.32~3.934) | 3.454(2.575~4.634) | 3.079(2.273~4.171) |
| lag16 | 0.866(0.368~2.036) | 0.873(0.376~2.026) | 0.895(0.406~1.975) | 0.896(0.424~1.894) | 0.875(0.436~1.756) | 0.83(0.443~1.555) | 0.765(0.446~1.314) | 0.697(0.442~1.101) | 0.649(0.436~0.966) | 0.639(0.45~0.908) | 0.688(0.527~0.898) | 0.824(0.731~0.929) | 1.082(1.04~1.125) | 1.527(1.311~1.779) | 2.259(1.837~2.779) | 3.165(2.408~4.159) | 3.645(2.689~4.94) | 3.245(2.368~4.446) |
| lag17 | 0.85(0.351~2.059) | 0.856(0.358~2.046) | 0.874(0.385~1.983) | 0.872(0.402~1.893) | 0.849(0.412~1.749) | 0.805(0.42~1.544) | 0.742(0.423~1.302) | 0.677(0.421~1.089) | 0.632(0.417~0.957) | 0.625(0.434~0.901) | 0.677(0.513~0.894) | 0.818(0.722~0.927) | 1.085(1.042~1.13) | 1.55(1.323~1.817) | 2.325(1.877~2.879) | 3.296(2.487~4.367) | 3.817(2.79~5.223) | 3.391(2.449~4.697) |
| lag18 | 0.835(0.336~2.078) | 0.84(0.342~2.061) | 0.852(0.366~1.982) | 0.846(0.38~1.881) | 0.821(0.389~1.731) | 0.777(0.396~1.524) | 0.717(0.4~1.283) | 0.655(0.4~1.075) | 0.615(0.399~0.947) | 0.611(0.418~0.895) | 0.667(0.5~0.89) | 0.812(0.713~0.925) | 1.088(1.043~1.134) | 1.571(1.333~1.851) | 2.383(1.912~2.97) | 3.411(2.555~4.555) | 3.967(2.874~5.477) | 3.512(2.509~4.917) |
| lag19 | 0.821(0.322~2.098) | 0.824(0.327~2.076) | 0.828(0.347~1.976) | 0.817(0.358~1.862) | 0.789(0.366~1.704) | 0.746(0.372~1.497) | 0.689(0.377~1.261) | 0.633(0.379~1.059) | 0.597(0.381~0.938) | 0.599(0.402~0.891) | 0.658(0.487~0.889) | 0.808(0.706~0.924) | 1.09(1.043~1.139) | 1.588(1.34~1.882) | 2.432(1.939~3.051) | 3.509(2.607~4.724) | 4.09(2.934~5.702) | 3.602(2.544~5.102) |
| lag20 | 0.808(0.307~2.124) | 0.809(0.312~2.096) | 0.803(0.328~1.97) | 0.786(0.336~1.839) | 0.755(0.341~1.674) | 0.713(0.347~1.466) | 0.66(0.353~1.236) | 0.61(0.357~1.043) | 0.58(0.362~0.93) | 0.587(0.387~0.889) | 0.65(0.475~0.89) | 0.804(0.698~0.925) | 1.092(1.043~1.143) | 1.602(1.342~1.911) | 2.472(1.954~3.125) | 3.587(2.638~4.878) | 4.182(2.964~5.902) | 3.658(2.546~5.256) |
| lag21 | 0.796(0.292~2.165) | 0.793(0.296~2.128) | 0.777(0.307~1.97) | 0.753(0.311~1.819) | 0.719(0.315~1.644) | 0.678(0.32~1.436) | 0.63(0.328~1.212) | 0.587(0.335~1.028) | 0.563(0.343~0.924) | 0.575(0.372~0.89) | 0.643(0.463~0.895) | 0.8(0.69~0.928) | 1.093(1.042~1.147) | 1.612(1.339~1.94) | 2.501(1.956~3.198) | 3.643(2.64~5.027) | 4.241(2.953~6.09) | 3.678(2.511~5.387) |

Table S30 Sensitivity analysis of different parameters of random forest model

| Disease | ntree | mtry | RMSE | *R*^2^ |
| --- | --- | --- | --- | --- |
|  |  |  |  |  |
| HFMD | 500 | 2 | 539.967 | 0.827 |
|  | 1000 | 2 | 538.290 | 0.826 |
|  | 1500 | 2 | 538.586 | 0.826 |
|  | 500 | 3 | 524.634 | 0.821 |
|  | **1000** | **3** | **525.344** | **0.821** |
|  | 1500 | 3 | 525.977 | 0.820 |
|  | 500 | 4 | 528.727 | 0.810 |
|  | 1000 | 4 | 528.288 | 0.811 |
|  | 1500 | 4 | 528.682 | 0.811 |
| DV | 500 | 2 | 276.139 | 0.874 |
|  | 1000 | 2 | 285.686 | 0.868 |
|  | 1500 | 2 | 288.727 | 0.865 |
|  | 500 | 3 | 263.681 | 0.881 |
|  | 1000 | 3 | 262.348 | 0.879 |
|  | 1500 | 3 | 262.865 | 0.880 |
|  | **500** | **4** | **244.774** | **0.888** |
|  | 1000 | 4 | 247.097 | 0.888 |
|  | 1500 | 4 | 249.813 | 0.885 |
| DN | 500 | 2 | 62.166 | 0.698 |
|  | 1000 | 2 | 62.482 | 0.694 |
|  | 1500 | 2 | 62.563 | 0.694 |
|  | 500 | 3 | 60.944 | 0.687 |
|  | 1000 | 3 | 61.074 | 0.687 |
|  | 1500 | 3 | 61.337 | 0.685 |
|  | **500** | **4** | **59.059** | **0.702** |
|  | 1000 | 4 | 59.095 | 0.701 |
|  | 1500 | 4 | 59.545 | 0.697 |
