# Supplementary material for: Temperature-humidity synergistic effects on predominant intestinal infectious diseases in Shenzhen, China: A predictive modeling framework for epidemiological early warning systems
Source: PLoS One. 2025 Dec 5;20(12):e0337929. doi: 10.1371/journal.pone.0337929 (PMC12680148; doi:10.1371/journal.pone.0337929)
Supplement: S1 File — S1 Table. Descriptive result of correlation analysis between three kinds of intestinal infectious diseases and meteorological factors. S2 Table. Result of model analysis. S3 Fig. Epidemic profiles and model fitting. (ZIP) [file pone.0337929.s001.zip › S3 Fig.docx]

**Supplementary material**

Fig 1. Monthly distribution of three common intestinal infectious diseases in Shenzhen from 2012 to 2022


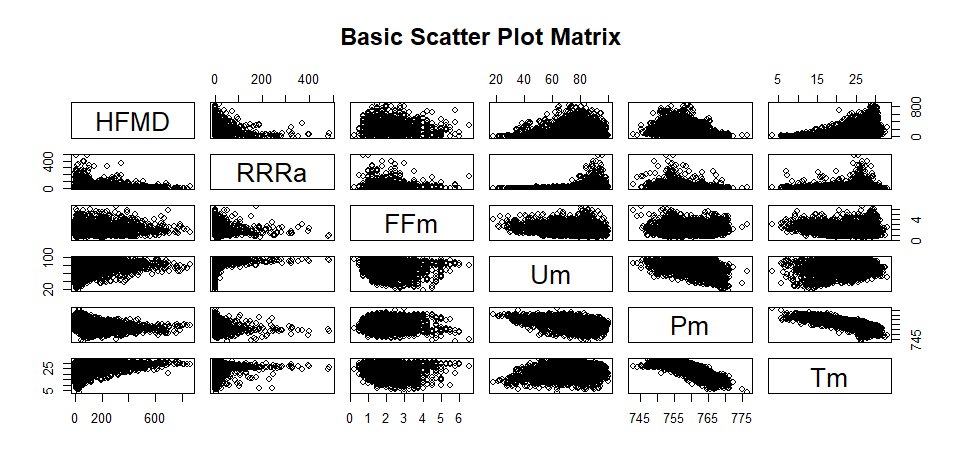


Fig 2. Correlation between HFMD and meteorological factors in Shenzhen, 2012-2022


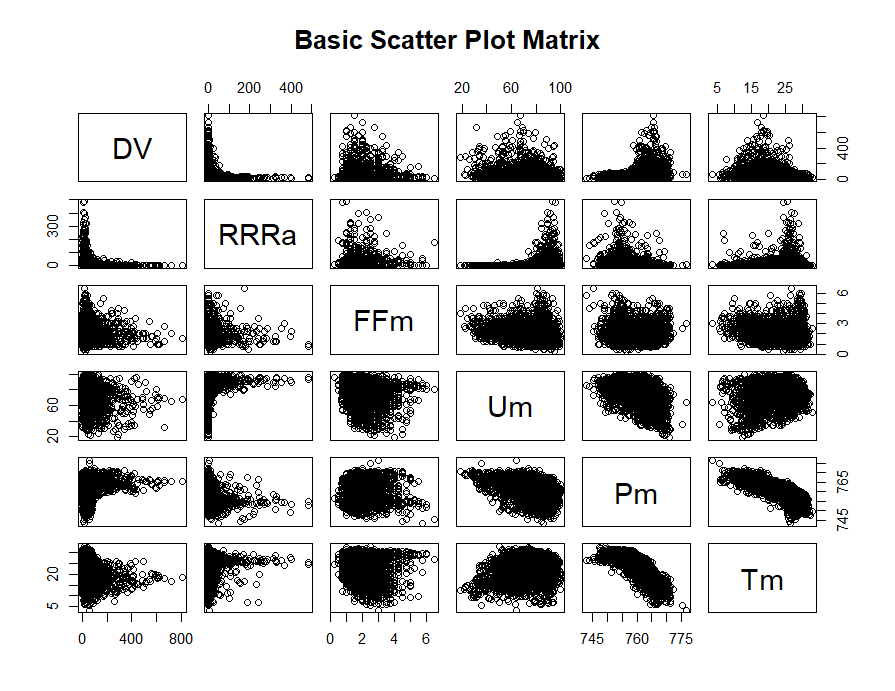


Fig 3. Correlation between HFMD and meteorological factors in Shenzhen, 2012-2022


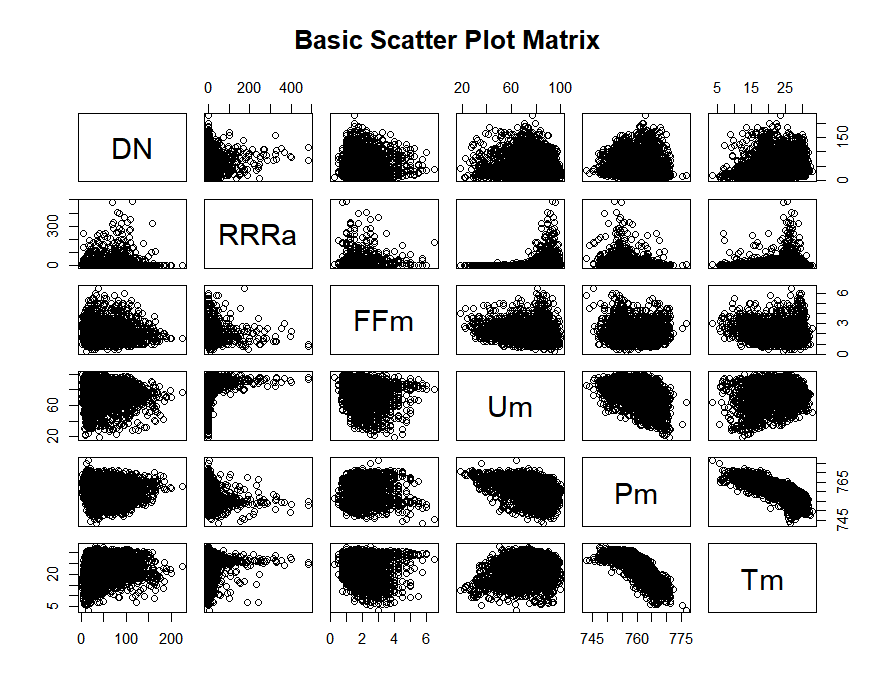


Fig 4. Correlation between HFMD and meteorological factors in Shenzhen, 2012-2022


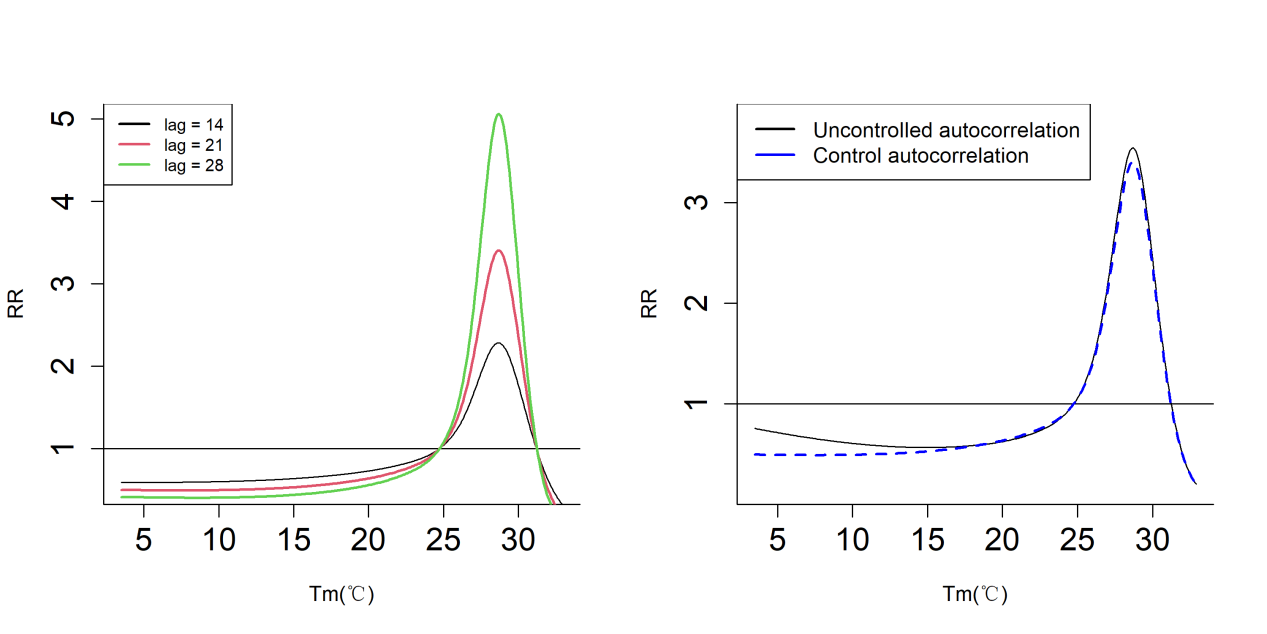


Fig 5. The overall effect of temperature on HFMD at different lag period, and with or without autocorrelation control


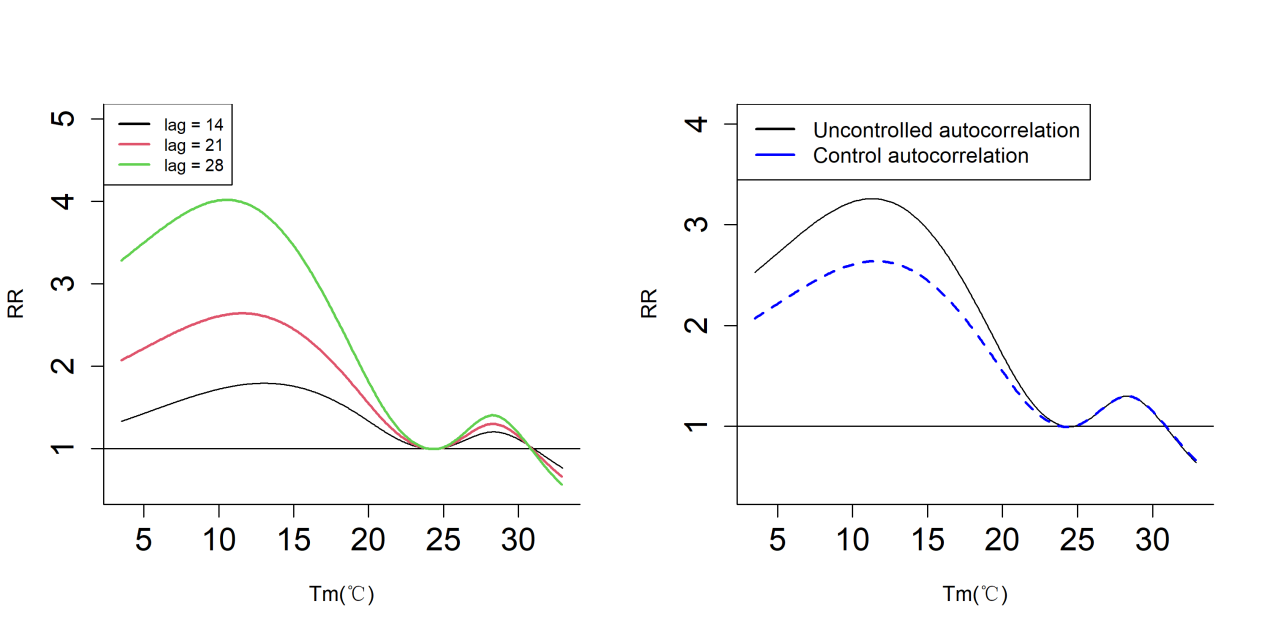


Fig 6. The overall effect of temperature on HFMD at different lag period, and with or without autocorrelation control


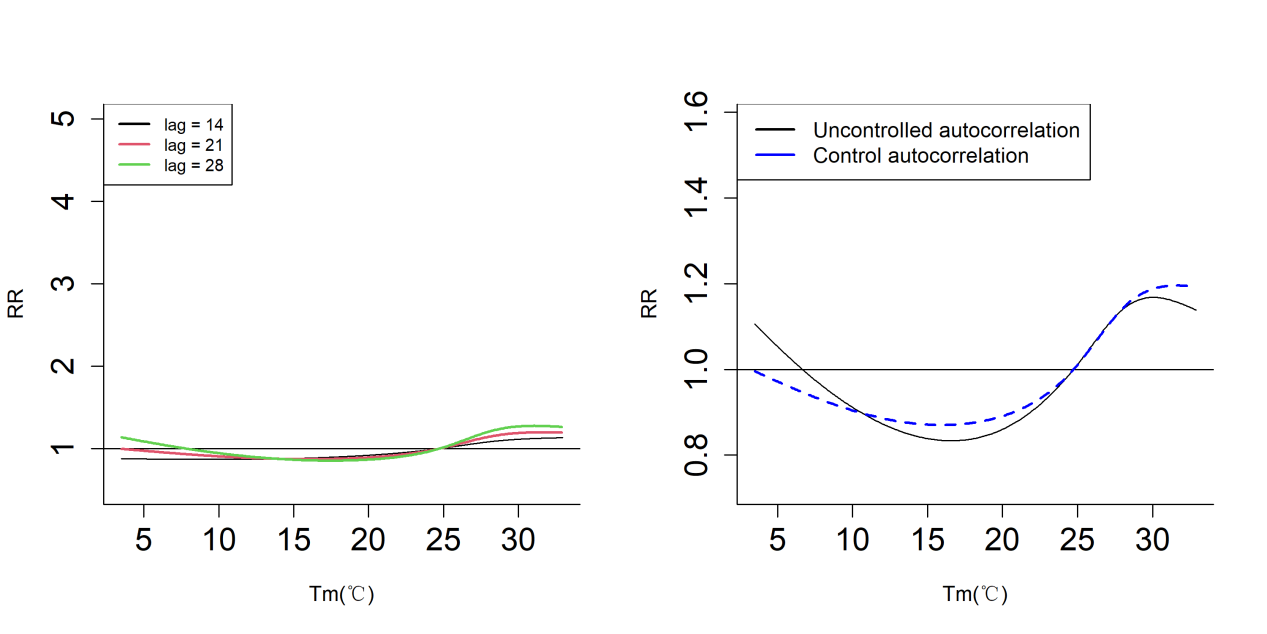


Fig 7. The overall effect of temperature on HFMD at different lag period, and with or without autocorrelation control
