# Supplementary material for: Temperature-humidity synergistic effects on predominant intestinal infectious diseases in Shenzhen, China: A predictive modeling framework for epidemiological early warning systems
Source: PLoS One. 2025 Dec 5;20(12):e0337929. doi: 10.1371/journal.pone.0337929 (PMC12680148; doi:10.1371/journal.pone.0337929)
Supplement: S1 File — S1 Table. Descriptive result of correlation analysis between three kinds of intestinal infectious diseases and meteorological factors. S2 Table. Result of model analysis. S3 Fig. Epidemic profiles and model fitting. (ZIP) [file pone.0337929.s001.zip › S1 Table.docx]

**Supplementary material**

**Descriptive result**

| **Table S1 Correlation analysis between three kinds of intestinal infectious diseases and meteorological factors** | | | | | |
| --- | --- | --- | --- | --- | --- |
|  | Tm | Um | Pa(kPa) | Fm(m/s) | RRRa |
| HFMD | 0.621 | 0.163 | -0.553 | -0.002 | 0.151 |
| DV | -0.345 | -0.175 | 0.372 | -0.042 | -0.183 |
| DN | 0.231 | -0.053 | -0.14 | -0.13 | 0.04 |
| * *p*<0.01. Tm,Um,Pa,Fm and RRRa represents temperature(℃), humidity(%), air pressure(kPa), wind speed(m/s), and rainfall(mm/d). | | | | | |
